# Evolution and main causes of the rising legionellosis incidence in the United States

**DOI:** 10.1371/journal.pgph.0007077

**Published:** 2026-09-09

**Authors:** Xiang Y. Han

**Affiliations:** Department of Laboratory Medicine, The University of Texas MD Anderson Cancer Center, Houston, Texas, United States of America; Tohoku University Graduate School of Engineering School of Engineering: Tohoku Daigaku Daigakuin Kogaku Kenkyuka Kogakubu, JAPAN

## Abstract

Legionellosis is an acute respiratory infection caused by the environmental bacteria *Legionella pneumophila* and other *Legionella* species upon inhalation of aerosolized bacteria. The legionellosis incidence in the United States has risen rapidly in the past two decades. This study examined the ecological evolution and main causes of the rising legionellosis incidence in the US from 1999 to 2021. Publicly available data on the annual and monthly infection rates, temperature and precipitation, motor vehicle miles driven, and solar ultraviolet were used for fitting analyses, linear regressions, seasonal correlations, and state-wise year-to-year comparisons. The annual and monthly mean temperature and precipitation in the US from 1895 to 2021 were examined first to show significant climate changes in the recent 4 decades. During 1999–2019 before the COVID-19 pandemic, the age-adjusted legionellosis incidence rates rose exponentially by 6.6-fold from 0.40/100,000 in 1999 to 2.37/100,000 in 2019 with a peak of 2.69/100,000 in 2018. During COVID-19, however, the rates dropped to 1.67/100,000 in 2020 and 2.21/100,000 in 2021, which paralleled the reduction of motor vehicle miles for COVID containment. In regression, the annual incidence rates during all 23 years correlated strongly with cumulative temperature anomaly, annual precipitation amount, and motor vehicle mileage. Analyses of the monthly incidence rates during 1999–2019 revealed emergence of a seasonal legionellosis incidence plateau in 2018 and 2019 that spanned from June to October. Interactions between monthly precipitation and motor vehicle traffic were most notable during COVID, supporting the notion of road exposure to soil-derived *Legionella* spp. In state-wise analyses, the above effects were seen consistently across diverse landscapes and climates. Together, four decades of warming temperature have driven the rapidly rising legionellosis incidence in the US. Road exposure to soil-derived bacteria likely constitutes the main exposure route upon interactions of motor vehicle traffic, precipitation, and attenuation or lack of sunlight.

## Introduction

Bacteria are vegetative organisms that require adequate nutrients, temperature, and water activity for proliferation. For environmental bacteria, their growth and abundance are dictated by a combination of habitats, climate, seasons, and sunlight. The aquatic and soil bacteria *Legionella pneumophila* and other *Legionella* spp. cause legionellosis in humans, an acute respiratory infection [1,2]. The infection is acquired through inhalation of bacteria-bearing aerosols upon an incubation period of 2–19 days [3,4]. Outbreaks as well as sporadic infections may occur under the influence of many human and natural factors.

Legionellosis outbreak occurs after cluster exposure to aerosols that are emitted from a single contaminated water source, such as hot tubs, cooling towers, or fountains [3–6]. Excavated soil may also generate aerosols or air-borne dust to cause outbreaks [7,8]. Most outbreaks occur in warm seasons, in reflection of the abundance of *Legionella* bacteria that proliferate optimally at 25–42°C [2,9]. Rare outbreaks in the winter may arise from the release of aerosols from warm cooling towers and tend to spread to a distance [5]. It is realized that the survival and distant dispersal of bacteria-laden aerosols in the winter may be aided by weak sunshine, cold temperature (refrigeration), and presence of fog or high humidity [10,11]. Outbreak investigation may inform the source of bacteria, generation and dynamics of aerosols, exposure opportunities, climate effects, and host predispositions.

Unlike outbreaks, sporadic infections occur individually without well-defined common denominators in time and space; in fact, the exposure and source of bacteria are rarely identified, making such infections incidental and almost random events. Yet, studies have also shown strong effects of climate and seasonal factors on the occurrence of sporadic cases in a region [12–15]. For instance, legionellosis cases may follow surplus rainfalls by days to two weeks in the mid-Atlantic states in the summer to hint exposure during rainfalls [12,13]. Drought, on the other hand, reduces sporadic cases, as seen in 2012 in the United States and in 2017 and 2018 in California [11]. Significantly, sporadic infections account for most legionellosis cases, from 75-89% in earlier decades [2,16] to 95% in the recent decade [17]. In outbreak as well as sporadic cases, vulnerable host factors include older age (≥ 65 years), existing broncho-pulmonary illness, immune defects or suppression, cancer, and others [1–3,18].

The legionellosis incidence in the US has risen rapidly in the past two decades along with a wide regional variation [11,19,20]. Recent studies on incidence changes, using data from 1999 to 2018, have attributed the rise and variation to climate changes, mixed solar and climate effects, rising road exposure, and aging population [11,19]. In brief, the incidence rise is exponential and parallels cumulative effects of above-norm warmer temperature since 1997 and precipitation surplus since 2008; warm temperature and precipitation proliferate *Legionella* spp. in soil and natural water to promote the incidence whereas strong sunlight suppresses it, and these factors interact in a mixed and dynamic fashion leading to incidence variation in seasons and across geographic regions; precipitation events may deposit soil bacteria to road surfaces, attenuate germicidal solar ultraviolet to extend bacterial viability, and generate aerosols during motor vehicle traffic to expose drivers and passengers; increasingly warmer and wetter climate has likely escalated bacterial density through accumulation, and along with rising traffic volume, led to more road exposure; and aging population increases the pool of vulnerable hosts, particularly those ≥ 65 years of age.

The realization of road exposure to soil *Legionella* likely uncovers a dominant and long-sought exposure route in the acquisition of infection, particularly for sporadic cases in the US, in view of daily driving for most Americans. The vehicle mileage driven in the US is vast and increasing, having reached 3240 billion miles or 9882 miles (15904 km) per capita in 2018 [11]. Moving motor vehicles spin up large quantities of aerosols on wet roads, some of which may harbor soil-derived bacteria. Many earlier and recent studies have demonstrated the ubiquity of *Legionella* spp. in soil [21–27], particularly wet soil [21], isolation of viable bacteria from rainwater puddles on road surfaces [27,28], and ventilation intake of road aerosols and bacteria by motor vehicles [29,30], thereby closing the loop of road exposure. Experimentally, exposure to sunlight or ultraviolet kills *L. pneumophila* [31,32], which accord with the above-mentioned solar control on legionellosis. Exposure to the soil *Legionella* spp. also likely underlies the risks noted previously with construction, excavation, transportation, or travel [33–38].

During the COVID-19 pandemic, the driving patterns in the US changed greatly. Since the start of the pandemic in early 2020, containment efforts have reduced driving drastically, to a nearly complete halt during lockdowns – in March-April in most states, and slow recovery thereafter. This sudden change presented an unprecedented opportunity, though very unfortunate, to examine the role of reduced traffic on legionellosis incidence.

In this study we examine the US legionellosis incidence rates in 1999–2021, on extension of the refined datasets in 1999–2018, to model the incidence trend and effects of climate changes and road exposure before and during the COVID-19 pandemic. Monthly data from the 23 years are also analyzed to characterize the seasonal changes in legionellosis in a year, the evolution of the rising monthly incidence in the decades before COVID, and the timing of reduced motor traffic during COVID. Extensive state-wise data in representative years are used to showcase the main causes of the year-to-year incidence changes among the states with diverse geography and climate. Fitting trends, seasonal patterns and correlations, and linear regression models are the main analytic approaches.

## Materials and Methods

### Data sources, definitions, and derived parameters

The legionellosis case data from 1999 to 2021 were from the annual and weekly reports of notifiable diseases from the Centers for Disease Control and Prevention (CDC) [39]. The weekly reports listed provisional number of cases from the current week and corresponding refined cases from the prior year; this study used refined case numbers. The weekly cases were also converted to monthly cases according to the calendar. If a week spanned two months, average daily cases of the week were used for calendar integration. The diagnosis of legionellosis in the US requires a specific test for *Legionella* spp., such as cultivation, urine antigen test, and/or a 4-fold increase of serologic titers, for distinction from diverse etiologic agents of pulmonary infection. These tests have been developed and used since the early 1980s, along with reporting to CDC, and from 1980 to 1998, the legionellosis incidence remained stable in the US with ~1,200 cases annually [2,20].

The population data were from the US Census publications or datasets [40] using projections 1995–2050 for the 1999 population with correction from the 2000 census, projections 2008 and 2009 (averaged) for 2000–2011, projection 2012 for 2012 and 2013, projection 2014 for 2014 and 2015, projection 2017 for 2016–2019, and national population characteristics of 2020–2024 for 2020 and 2021. Annual incidence rates in cases per 100,000 population were calculated according to age groups of 0–39 years, 40–64 years, ≥ 65 years, and all ages. Age-adjusted incidence rates were also used, based on the census age brackets in 2000, to correct the bias of aging population. Monthly incidence rates were calculated as: (monthly cases ÷ annual cases) x age-adjusted annual incidence rate.

The temperature and precipitation data for the contiguous US (since 1895) were from the National Centers for Environmental Information [41]. Annual, monthly, and seasonal data were extracted directly from the source. Seasonal data included warm months (May to October) and cold months (November to April). Long-term 100-year mean values from 1895 to 1994 were set as benchmark norms to derive yearly, monthly, or seasonal anomalies during 1995–2021. Yearly temperature anomaly values, when summed up, yielded cumulative anomalies. These temperature and precipitation data were continuous from 1895 to 2021.

The solar ultraviolet B (UVB) data was from the UVB Monitoring and Research Program at Colorado State University [42] using web-based searches [43]. The program measures daily UVB at wavelengths of 280–320 nm, the main germicidal part of solar radiation, in kilojoules per square meter per day/hour (KJ/m^2^ day/hr.), at 32 sites in the continental US and one site in Canada (Ontario). The coordinates range from 25.39 to 48.31°N in latitude and from 68.04 to 121.78°W in longitude. Due to a change in data presentation in 2020, the annual data for 2020 and 2021 were incongruent with our previous dataset from 1999 to 2018 that was derived from the source [11]. Thus, this study opted to use the new data format from 2018 to 2021 for monthly analysis only. Daily readings at each site were averaged to monthly data first. Then, the monthly readings from all sites were integrated into a multiple linear regression equation with coordinates. To derive a monthly UVB in the US, the coordinates of 39°N and 95°W were used for the equation.

The annual and state-wise motor vehicle mileage data from 1999 to 2021 were from the Bureau of Transportation Statistics [44]. Monthly vehicle mileage data from 2018 to 2021 were from Federal Reserve Bank of St. Louis [45]. These data were continuous.

### Data quality and improvement

Due to the long timespan and US nationwide data reporting and collection, rare instances of typographic errors, reporting delays, or lack of reporting in certain weeks or years and/or in some states were noted in the CDC data source. Efforts were made to identify and correct these data quality issues prior to analysis. Below are some examples and correction methods.

During the 23 study years with 98,212 legionellosis cases, there were 1,331 cases (1.36%) without age data. The age brackets of these cases were prorated according to the known ones each year. Two states, West Virginia in 1999–2002 and Oregon in 1999–2001, did not report legionellosis cases. These states with relevant annual data in population, motor vehicle mileage, precipitation, and temperature were excluded from analyses. These corrected data were supplemented (S1 Table). Thus, the annual US incidence rates and related data from 1999 to 2002 were refined from the previous dataset [11].

In 2018, Washington lacked weekly reports from weeks 22–51 until the final report in week 52. To mitigate the reporting delay that involved 34 cases, the case numbers in these weeks were estimated according to the weekly patterns in 2013 and 2014 that were deemed reasonably accurate and averaged. Similar 2018 weekly reporting delays also occurred to Oklahoma that involved annual 63 cases and to Georgia that involved 49 cases in weeks 12–22. These delays were similarly corrected using the most recent patterns.

In 2019, six states showed reporting delays or apparent inaccuracies: Oklahoma that involved annual 52 cases; New Jersey that involved 196 cases in weeks 23–38; Indiana that involved 89 cases in weeks 1–28; Texas that involved 41 cases in week 32 and weeks 45–52; Washington that involved 25 cases in weeks 39–52; and Ohio that involved 10 cases in week 49. All these were corrected according to usual weekly patterns or best estimates in these states.

In 2020, due to the disruption of COVID-19, twelve states showed reporting delays of 834 cases among a total of 6310 cases. These delays were corrected according to the best estimates from the pattern in pre-COVID years. In 2021, Michigan, Ohio, Oklahoma, and Texas showed delays in various weeks, resulting in correction of 462 cases. Together, the adjustments rectified delays and improved accuracy in the weekly US cases in 2018 by 1.5% (146 of all 9933 cases), in 2019 by 4.6% (413 of 8890), in 2020 by 13.2% (834 of 6310), and in 2021 by 5.5% (462 of 8442). These corrected data were supplemented (S1 Table).

Weekly data from 1999 to 2017 in some states and/or weeks were similarly corrected for errors and/or reporting delays. The corrections of weekly cases did not affect the annual data but improved weekly and monthly data for related analyses.

### Data management and analysis

Data handling and analyses used mainly Excel version 2016 [46], including plots for trend, fitting models, correlations, and linear regressions between various parameters (explanatory variables) and incidence rates or cases (outcome variables). Explanatory variables included temperature, precipitation, UVB, and interactive precipitation x motor vehicle miles. While the inclusion of temperature and precipitation were based on their well-known effects, the UVB and motor vehicle miles were extended from our previous studies [11,19].

In view of the exponential rise of the incidence rates, the linear regressions were performed upon logarithmic transformation of the incidence rates. Simple Log-linear regressions were applied to each of the climate and vehicle mileage parameters first, followed by multiple Log-linear regressions with two to all parameters to assess the promotive and/or inhibitive role of each parameter. For regressions with 2018–2021 monthly data, the explanatory variables were adjusted ahead for 14 days using prior month data to account for the incubation period of legionellosis. In view of the time series nature of the datasets, proper interpretation of significant correlations required biological relevance for support. Cautions were taken during multiple regressions to recognize and avoid spurious results due to data comparability or inherent relatedness, known as multicollinearity [47].

It was noted that the annual incidence rates, with decimal points, were unsuitable for Poisson regression analysis that requires integers. Instead, they were taken as a normal approximation to Poisson distribution for linear regression analysis, a practice in epidemiology for large scale studies [48], such as the recent US legionellosis studies [11,19]. The use of Log linear regressions was also close to Poisson regressions (with natural Log scale). Data in Poisson distribution approximate normal distribution when their mean value is large (λ > 50).

For the analyses of 2018–2021 monthly data, in addition to monthly age-adjusted incidence rates, monthly cases were also used to satisfy the integer requirement of Poisson distribution. SPSS (version 29) was used for Poisson regressions and negative binomial regressions. The normality of variables was assessed with Kolmogorov-Smirnov test.

## Results

### Long-term climate changes since 1895

Warm temperature is required for the proliferation of environmental bacteria *Legionella* spp. To further characterize the climate effects on the evolution of legionellosis incidence from 1999 to 2021, particularly 2020 and 2021 during the COVID-19 pandemic, the long-term trends of temperature and precipitation in the US from 1895 to 2021 were examined first in considerable detail.

**Fig 1:**
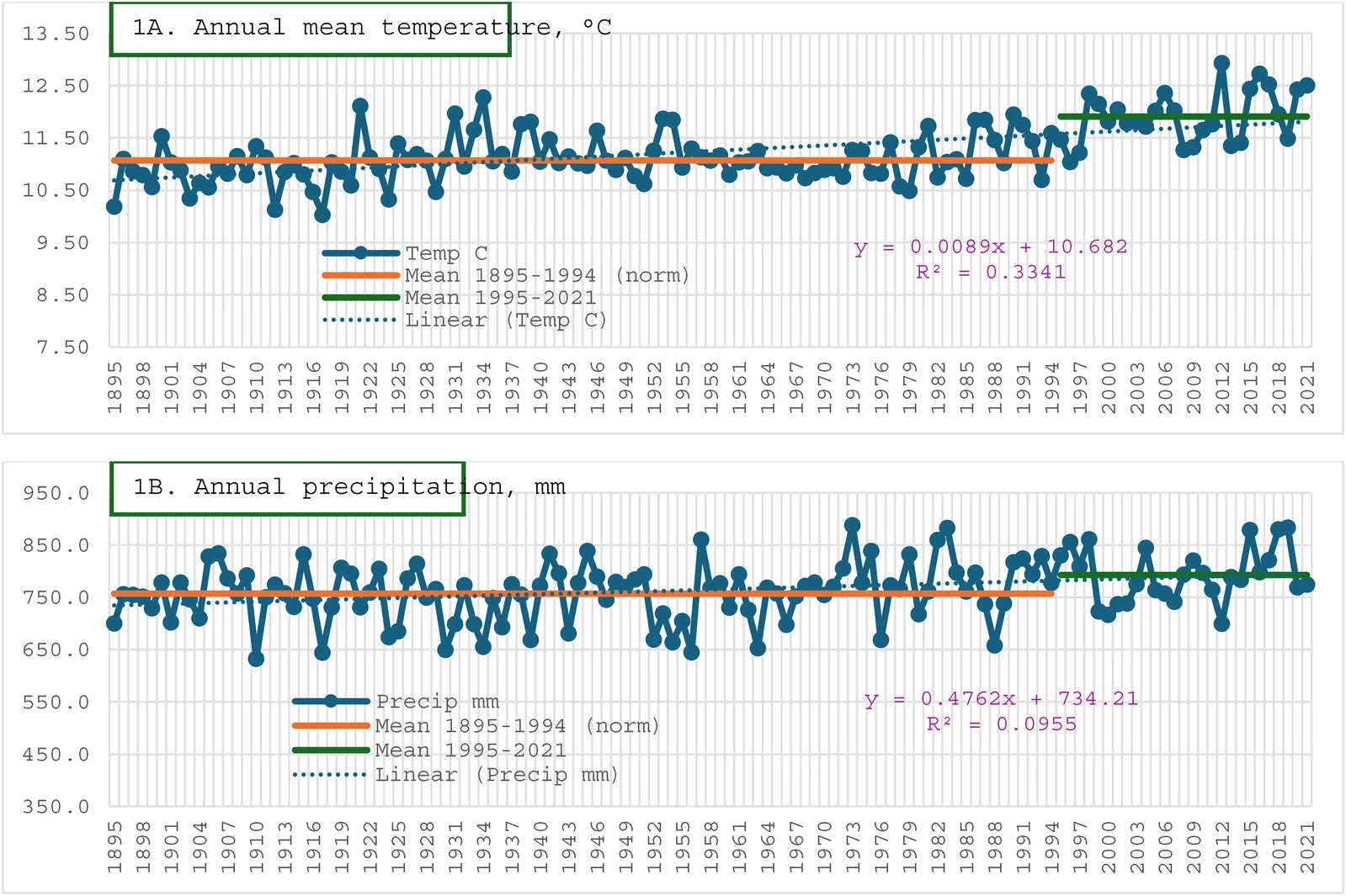
Trend of annual mean temperature (1A) and annual precipitation (1B) in the continental United States, 1895-2021. Y axis = parameter; X axis = year.

Since the start of US climate records in 1895, the annual mean temperatures and annual precipitation levels have undergone changes gradually despite fluctuations. As shown in Fig 1 and Table 1, both temperature and precipitation trended upward during 127 years, with R^2^ of 0.3341 and 0.0955, respectively. The temperature trend became more consistent since 1982 (Table 1 footnote). With means of the first 100-year data (1895–1994) serving as benchmarks, the temperatures and precipitation levels from 1995 to 2021 have risen significantly by an average of +7.6% (*P =* 2.6E-14) and +4.7% (*P =* 0.003), respectively. In particular, the annual mean temperatures have been above norm consecutively since 1997 for 25 years. The latest decade of 2012–2021 marked the warmest and wettest decade, with average temperature above norm by +10.0% (*P =* 2.7E-11) and precipitation by +6.7% (*P =* 0.008). The decade included 6 warmest years on records, in descending order of 2012, 2016, 2017, 2021, 2015, and 2020, that were above the norm by 1.86 to 1.36^o^C (+16.8 to 12.3%).

**Table 1:** Long-term trends of annual and seasonal mean temperature and precipitation in the continental United States, 1895-2021.

Years or months	No. of years	Mean temperature, °C			Precipitation mm		
		Mean ± SD	Rise (%)	*P* value	Mean ± SD	Rise (%)	*P* value
Annual							
1895–1994 (norm)	100	11.07 ± 0.4348	Baseline	--	757.1 ± 55.79	Baseline	--
1995-2021	27	11.91 ± 0.5050	0.84 (7.6)	2.6E-14	792.8 ± 51.93	35.7 (4.7)	0.003
Latest 2012–2021	10	12.18 ± 0.5813	1.11 (10.0)	2.7E-11	807.6 ± 59.22	50.5 (6.7)	0.008
Trending 1895–2021	127	R^2^ = 0.3341, y = 0.0089x + 10.68			R^2^ = 0.0955, y = 0.476x + 734.2		
Warm May to October							
1895–1994 (norm)	100	18.63 ± 0.4329	Baseline	--	401.7 ± 35.35	Baseline	--
1995-2021	27	19.24 ± 0.4235	0.61 (3.3)	1.7E-09	429.2 ± 34.63	27.5 (6.8)	4.5E-04
Latest 2012–2021	10	19.51 ± 0.3388	0.89 (4.8)	7.3E-09	443.2 ± 37.64	41.4 (10.3)	6.5E-04
Trending 1895–2021	127	R^2^ = 0.2051, y = 0.0061x + 18.37			R^2^ = 0.1035, y = 0.3219x + 387.0		
Cold November to April							
1895–1994 (norm)	100	3.53 ± 0.6596	Baseline	--	355.9 ± 36.64	Baseline	--
1995 Nov.-2022 Apr.	27	4.58 ± 0.8135	1.05 (29.6)	1.9E-10	361.3 ± 38.81	5.5 (1.5)	0.50
Trending 1895–2021	127	R^2^ = 0.2634, y = 0.0114x + 3.03			R^2^ = 0.0183, y = 0.1359x + 348.3		

Annual mean temperature trending 1982–2021, R^2^ = 0.3488, y = 0.0282x + 11.14; all parameters increased from the annual trending 1895–2021, suggesting more consistent rise during 1982–2021.

Warm May to October mean temperature trending 1981–2021, R^2^ = 0.4822, y = 0.0273x + 18.47; all parameters increased from the trending 1895–2021 of these months, suggesting more consistent rise during 1981–2021.

Cold November to April mean temperature trending 1981–2021, R^2^ = 0.1482, y = 0.0254x + 3.85; both the slope and intercept increased from the trending 1895–2021 of these months, suggesting faster rise during 1981–2021.

Across the US, there were considerable regional variations in the upward trend of anomalous temperature. For instance, the coastal states, such as the Atlantic New Jersey and Pacific California, have experienced steady temperature rise since around 1980, ~ 15 years ahead of those in central states, such as Illinois and Missouri (S1 Fig and S2 Table). Among several representative states, the highest R^2^ of 0.4972 and highest slope of 0.0166 were noted in New Jersey. The slope of 0.0166 meant a temperature rise of 0.0166^o^C per successive year, or 1.66^o^C in 100 years. By contrast, the slope of 0.007 in Illinois meant a rise of 0.007^o^C per year or 0.7^o^C in 100 years. Cold northern states, such as Wisconsin, have also shown relatively higher temperature rise, relative to norm, than warm southern states.

Seasonally, during 1995–2021, the mean temperatures in the cold months of November to April rose the most, from norm 3.53^o^C to 4.58^o^C (up 1.05^o^C, + 29.6%), while the precipitations in the warm months of May to October showed most surplus by +6.8% (from norm 401.7 to 429.2 mm) (Table 1). In fact, the mean November-April temperature started to rise in 1980, which resulted in an average of 4.40^o^C during 1980–2021, above the 1895–1979 average of 3.44^o^C by 0.96^o^C (+27.9%) (t = 7.48, *P =* 1.1E-11).

Examined by months in the 127 years, all monthly mean temperatures trended upward despite considerable year-to-year fluctuations (S3 Table). The cold months of December, January, and March showed most significant rises, in degrees and percentages, all above the prior century norms by ≥ 1.06^o^C during 1995–2021 and by ≥ 1.71^o^C (≥ + 35%) during 2012–2021. For the warm months, June and September recorded most rises, particularly during the decade of 2012–2021, both above the norm by ≥ 1.22^o^C (≥ + 6%). Accordingly, the number of warm days with daily mean temperature of ≥ 20^o^C (68^o^F) in a year rose steadily after 1995. For instance, these warm days averaged 80 days during 1895–1994 (from ~ June 14 to September 2) but 91 days during 1995–2021 (from ~ June 10 to September 9) and 98 days during 2012–2021 (from ~ June 6 to September 12) (both *P* < 0.001). More warm days would proliferate more *Legionella* spp. in the environment, like an incubator. In the ensuing cold months, although the bacterial density was diminished, warmer winter would raise the baseline to start a new annual cycle.

### Annual legionellosis incidence from 1999 to 2021 before and during COVID-19

**Fig 2:**
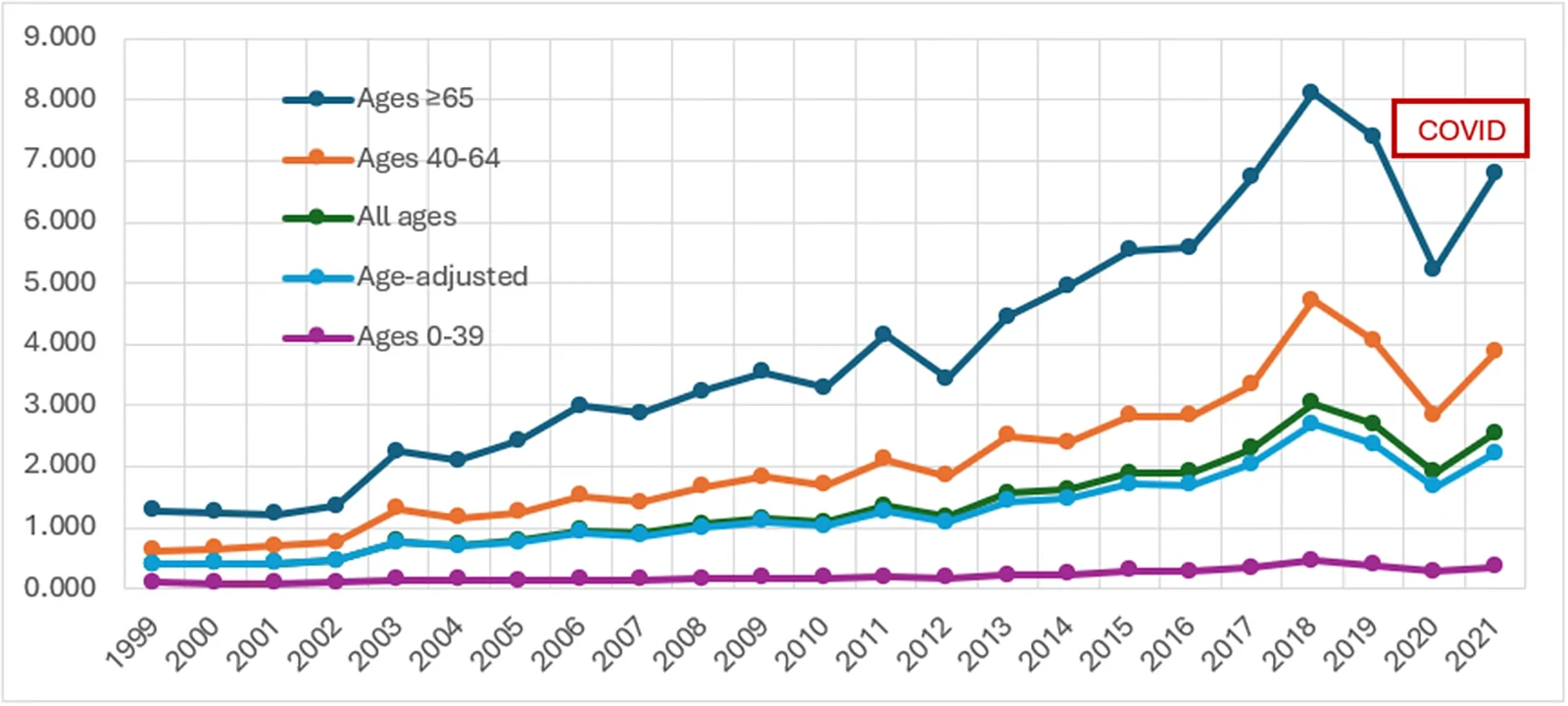
Annual age-specific, all ages, and age-adjusted legionellosis incidence rates before and during COVID-19 in the United States, 1999-2021. Y axis = Legionellosis incidence rate, cases/100,000; X axis = year.

Before the COVID-19 pandemic, the age-specific, all ages, and age-adjusted legionellosis incidence rates in 2019 continued the general upward trend from 1999 to 2018 in spite of being slightly lower than the 2018 peaks (Fig 2). During COVID-19 in 2020 and 2021, these rates all decreased, particularly prominent in 2020.

In fitting analyses, the incidence rates before COVID during 1999–2019 fitted exponentially as well as linearly (Table 2). By exponential fitting, the age-adjusted incidence rate rose by 6.6-fold during the 21 years, or annually 1.09-fold. The fitting trends would predict rising incidence rates in 2020 and 2021 or at least remain at peak levels like 2018 and 2019. However, with rampant COVID-19, the actual legionellosis incidence rates in 2020 dropped to 59.2 to 71.5% of the exponentially fitted rates for the age groups or overall, 61.5% for the age-adjusted incidence rate (Table 2). The 2021 actual incidence rates recovered to 74.8% of the predicted age-adjusted rate. In both years, the seniors of ≥ 65 years showed the most drop, to 59.2% in 2020 and 70.0% in 2021, a sign of least exposure to the *Legionella* bacteria in this population who are most vulnerable to both the bacteria and SARS-COV virus. By linear fitting, the 2020 age-adjusted incidence rate was at 74.2% of the projection whereas the 2021 rate reached 94.3%. Therefore, by either fitting, the legionellosis incidence rates in 2020 dropped remarkably during the peak of COVID-19. This finding prompted a quest for the cause.

**Table 2:** Fitting analyses of the legionellosis incidence rates in 1999-2019 (cases per 100,000 population) and comparison with those during COVID-19 in 2020-2021 in the United States.

Parameter	Age group (years)				Age adjusted
	0-39	40-64	≥ 65	All ages	
Exponential fitting, 1999–2019 trend, n = 21 years, before COVID					
Fitting R^2^	0.9079	0.94	0.9635	0.9591	0.9525
Equation Y1 =	0.0828e^0.0713x^	0.6121e^0.0917x^	1.1528e^0.0925x^	0.3702e^0.0965x^	0.3757e^0.0897x^
Yearly increase in fold*	1.074	1.096	1.097	1.101	1.094
21-year increase in fold	4.47	6.86	6.98	7.59	6.58
Linear fitting, 1999–2019 trend, n = 21 years, before COVID					
Fitting R^2^	0.8239	0.878	0.9209	0.8906	0.8946
Equation Y2 =	0.0146x + 0.0406	0.1675x + 0.1174	0.3145x + 0.2561	0.1137x + 0.0164	0.098x + 0.0905
Yearly increment	0.0146	0.1675	0.3145	0.1137	0.098
2020 incidence rates, during COVID, cases/100,000					
Actual	0.284	2.838	5.226	1.903	1.668
Y1 projected, x = 22	0.397	4.602	8.822	3.093	2.703
% actual ÷ Y1 projected	71.5%	61.7%	59.2%	61.5%	61.7%
Y2 projected, x = 22	0.362	3.802	7.175	2.518	2.247
% actual ÷ Y2 projected	78.5%	74.6%	72.8%	75.6%	74.2%
2021 incidence rates, during COVID, cases/100,000					
Actual	0.359	3.861	6.776	2.542	2.212
Y1 projected, x = 23	0.427	5.044	9.676	3.407	2.957
% actual ÷ Y1 projected	84.1%	76.5%	70.0%	74.6%	74.8%
Y2 projected, x = 23	0.376	3.97	7.49	2.632	2.345
% actual ÷ Y2 projected	95.5%	97.3%	90.5%	96.6%	94.3%

*Calculation examples:

Yearly increase = Exp (coefficient) in Excel.

21-year increase = Exp (21*coefficient) in Excel.

Y1 projection for the 0-39 age group in 2020 = 0.0828e^0.0713 * 22^ = 0.397.

Y2 projection for the 0-39 age group in 2020 = 0.0146 * 22 + 0.0406 =0.362.

### Effect of reduced motor traffic during COVID-19 on the legionellosis incidence

The trend and changes in legionellosis incidence before and during the COVID-19 pandemic were examined in Log-linear regression models with explanatory variables (Table 3, data in S4 Table). Before COVID, the 1999–2019 age-adjusted legionellosis incidence rates correlated best with cumulative temperature anomalies in simple regressions (R^2^ = 0.9508, *P =* 6.9E-14), suggesting accumulation of the promotive effect from decades of warmer temperature. Similar promotive effects were also found with vehicle miles driven, annual precipitation, and the multiplication of both variables – all pertaining to road exposure. By contrast, annual mean temperature showed no correlation at all (reasons to be delineated later). In multiple regressions, robust correlations were obtained to show synergistic effects of cumulative temperature anomaly and road exposure in terms of the interactive annual precipitation x vehicle miles (R^2^ = 0.9576).

**Table 3:** Linear regressions between Log-transformed annual age-adjusted legionellosis incidence rates (cases per 100,000 population) and concurrent temperature, precipitation, and vehicle miles driven in the United States, 1999-2021.

Regression and parameters	R^2^	Coefficient	*P* value	Interpretation
Before COVID, simple linear regression 1999–2019, n = 21 years				
Cumulative temperature anomaly, °C	0.9508	0.046	6.9E-14	Strongly promotive
Annual vehicle miles driven	0.8654	0.0014	1.0E-09	Strongly promotive
Annual precipitation x vehicle miles	0.7698	7.91E-07	1.8E-07	Strongly promotive
Annual precipitation, mm	0.5411	0.0033	1.4E-04	Promotive
Annual mean temperature, °C	0.0001	-0.0036	0.98	No correlation
Before COVID, multiple linear regressions 1999–2019, n = 21 years				
Adjusted R^2^	0.9576		1.7E-13	Robust correlation
Intercept		-0.9733	1.3E-04	
Cumulative temperature anomaly, °C		0.0413	3.6E-11	Strongly promotive
Annual precipitation, mm		6.5E-04	0.035	Promotive
Adjusted R^2^	0.9576		1.7E-13	Robust correlation
Intercept		-0.8410	1.7E-05	
Cumulative temperature anomaly, °C		0.0384	1.9E-08	Strongly promotive
Annual precipitation x vehicle miles		1.76E-07	0.035	Promotive
During and before COVID, simple linear regressions 1999–2021, n = 23 years				
Cumulative temperature anomaly, °C	0.9063	0.040	2.9E-12	Strongly promotive
Annual vehicle miles driven	0.7966	0.0014	1.0E-08	Strongly promotive
Annual precipitation x vehicle miles	0.6792	7.83E-07	1.3E-06	Strongly promotive
Annual precipitation, mm	0.4470	0.0032	4.9E-04	Promotive
During and before COVID, multiple linear regressions 1999–2021, n = 23 years				
Adjusted R^2^	0.9390		2.7E-13	Robust correlation
Intercept		-1.268	1.1E-05	
Cumulative temperature anomaly, °C		0.0349	1.9E-11	Strongly promotive
Annual precipitation, mm		0.0011	0.0014	Promotive
Adjusted R^2^	0.9511		3.0E-14	Robust correlation
Intercept		-1.044	6.6E-08	
Cumulative temperature anomaly, °C		0.0309	4.9E-10	Strongly promotive
Annual precipitation x vehicle miles		2.95E-07	1.3E-04	Strongly promotive

All data in S4 Table.

Strength of correlation: robust or strong = R^2^ value above 0.6 with p value at E-04 or less.

**Fig 3:**
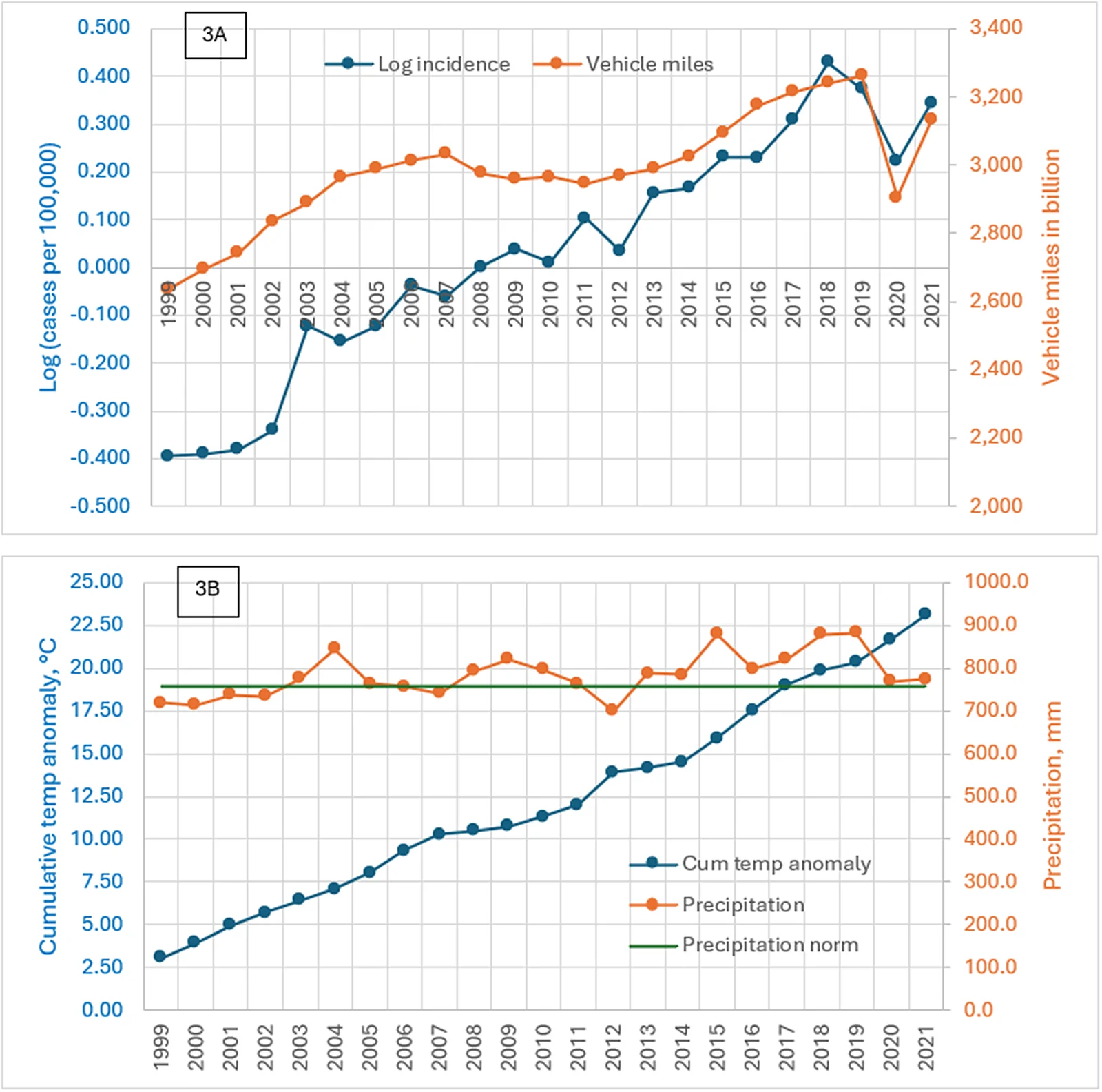
Log transformed annual age-adjusted legionellosis incidence rates in relation to annual vehicle miles driven (3A) and cumulative temperature anomaly and annual precipitation (3B) in the United States, 1999-2021. Y axis = parameter as shown; X axis = year.

During COVID-19 in 2020, enormous containment efforts reduced motor traffic to 2904 billion vehicle miles from the peak of 3262 billion miles in 2019, a setback to the 2003 level (Fig 3). The setback paralleled the fall in legionellosis incidence in 2020. As the road traffic recovered significantly in 2021, to 3132 billion miles driven, the legionellosis incidence rate rebounded accordingly. With the 1999–2021 data, the regressions remained robust, particularly notable in the multiple regressions that enhanced the fitting of road exposure (vehicle miles x precipitation) (R^2^ = 0.9511) (Table 3). The model predicted a mean difference of ~10% from the actual incidence rates or 90% accuracy (S5 Table).

Therefore, much of the legionellosis incidence drop in 2020 and 2021 could be attributed to less driving or road exposure. This conclusion also explained the most incidence drop among the seniors, as shown in Table 2, who would drive much less during COVID than the working population would. The incidence drops were also contributed by the near-normal precipitation levels in 2020 (769.1 mm, norm 757.1 mm) and 2021 (774.2 mm), far less than the near-record high levels in 2018 (880.1 mm) and 2019 (883.4 mm) (+16.2 to 16.7%).

The promotive effects of cumulative temperature anomaly and road exposure were best shown in New Jersey, previously during the pre-COVID years of 1999–2018 [11] and again with addition of the 2019–2021 data (S6 Table). In multiple regressions, the adjusted R^2^ values were 0.9540 during 1999–2019 and 0.9143 during 1999–2021 (both *P* ≤ 8.2E-12). New Jersey is a mid-Atlantic state with ample precipitation, moderate solar strength and sunshine hours, temperature at the US average, and legionellosis incidence rate slightly above the US average [11,19]. Remarkably, the state experienced a steady above-norm temperature run since 1979, earlier than the US national trend, which resulted in the century-highest rise of 1.36^o^C (+12.7% above the state norm) (S1 Fig and S2 Table). When the legionellosis incidence leaped in 2003 for the first time, the warmer temperature had run 25 years.

### Emergence of seasonal legionellosis incidence plateau in 2018 and 2019

**Fig 4:**
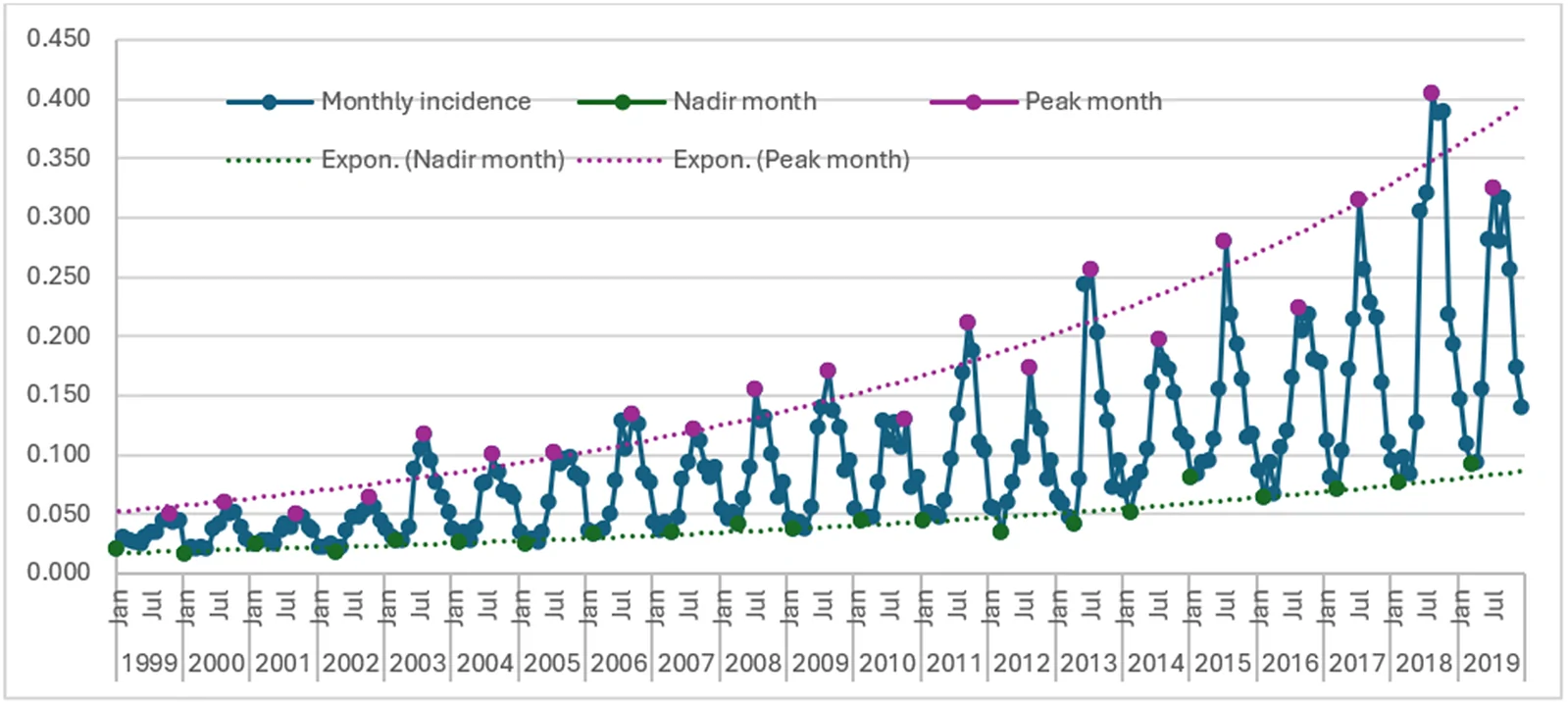
Evolution of the monthly legionellosis incidence rates before COVID-19 in the United States, 1999-2019. Y axis = Monthly incidence rate, cases/100,000; X axis = month and year.

The exponential rise of the annual legionellosis incidence rates from 1999 to 2019 before COVID-19 prompted a closer examination of the seasonal incidence evolution by months. As plotted in Fig 4, for all 21 years, monthly incidence rates followed a seasonal pattern every year and trended upward year after year. The rises were relatively steady in the cold months of November to April but faster in the warm months of May to October. They all fitted well exponentially (R^2^ = 0.9299 to 0.7952), with May being the best fitted month due to least fluctuations (Table 4). In the table, the monthly fitted coefficients also yielded incidence rises per year and for all 21 years. The rises in May to October averaged 7.7-fold overall (range 6.2- to 8.8-fold) while those in November to April averaged 5.0-fold (4.6- to 6.2-fold). The most rises occurred in May, June, and July, by 8.4, 8.5, and 8.8-fold, respectively, signifying early arrival of the infection.

**Table 4:** Data fitting analysis of the pre-COVID monthly legionellosis incidence rates in the United States, 1999-2019.

Month	Exponential fitting		Increase in fold	
	R^2^	Coefficient	Yearly	21-year
January	0.9133	0.0867	1.091	6.18
February	0.9078	0.0734	1.076	4.67
March	0.8933	0.0758	1.079	4.91
April	0.8673	0.0806	1.084	5.43
May	0.9299	0.1013	1.107	8.39
June	0.8121	0.1019	1.107	8.50
July	0.8444	0.1036	1.109	8.81
August	0.8716	0.0986	1.104	7.93
September	0.8525	0.0924	1.097	6.96
October	0.8025	0.0865	1.090	6.15
November	0.8510	0.0725	1.075	4.58
December	0.7952	0.0742	1.077	4.75
Annual	0.9524	0.0898	1.094	6.59
May-October	0.9209	0.0972	1.102	7.70
November-April	0.9575	0.0766	1.080	5.00
Nadir month	0.8952	0.0769	1.080	5.03
Peak month	0.9016	0.0962	1.101	7.54

Yearly increase = Exp(coefficient) in Excel.

21-year increase = Exp(21*coefficient) in Excel.

**Fig 5:**
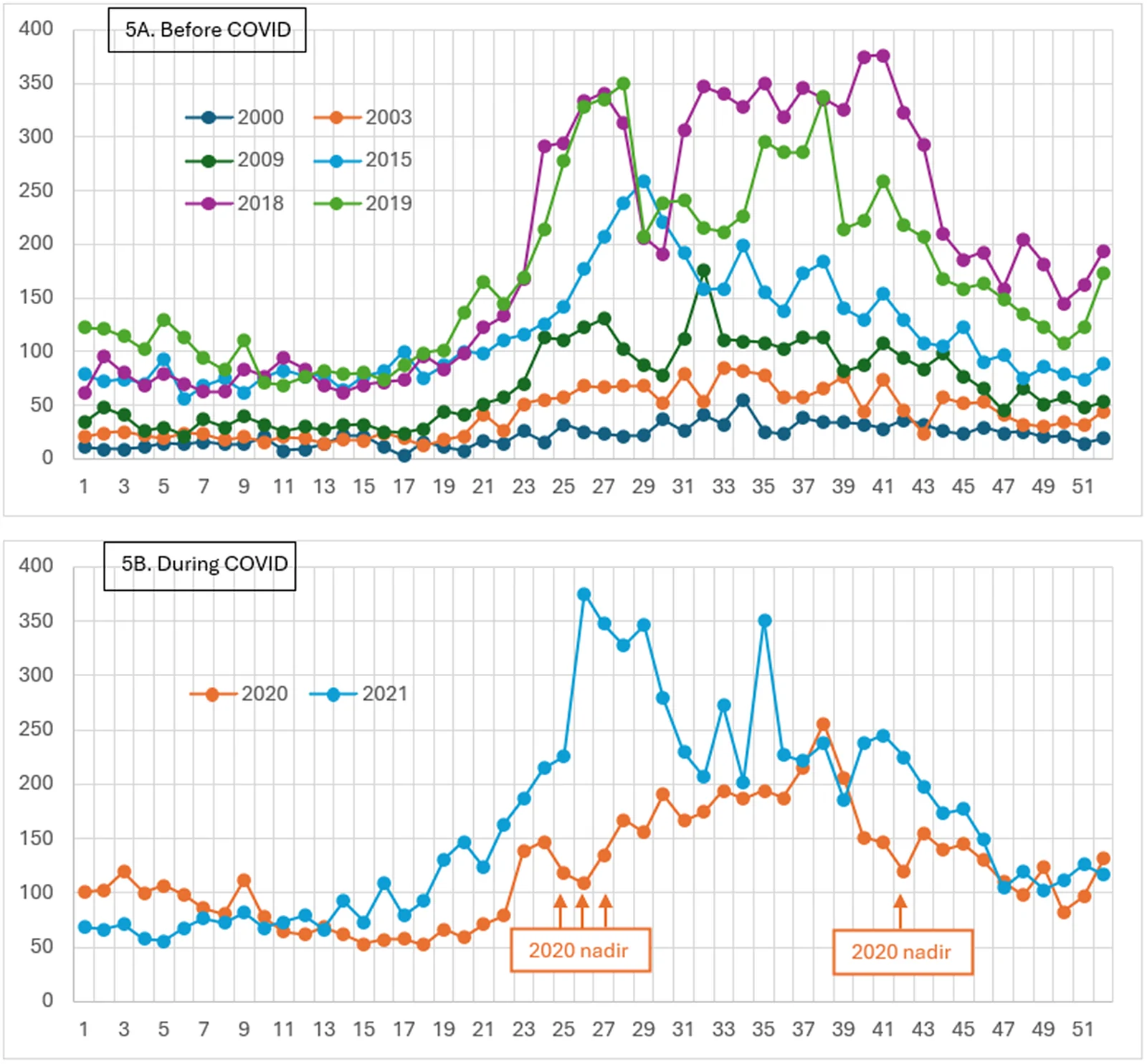
Weekly legionellosis cases in the United States showing the emergence of seasonal incidence plateau in 2018 and 2019 (5A) and drops during COVID in 2020 and 2021 (5B). Y axis = weekly cases; X axis = week.

The evolution of seasonal legionellosis incidence was further resolved by weekly cases in several representative years (Fig 5A). Compared to the low number of weekly cases in 2000 and 2003, an apparent weekly peak appeared in 2009 (week 32, August 9–15, with 175 cases), which turned into a monthly peak in 2015 (weeks 27–31, July 5 to August 8, weekly 192–259 cases). In 2018, the seasonal peak arrived early and subsided late to become a wide and tall incidence plateau, with weekly 192–376 cases, that spanned 140 days from weeks 24–43 or June 10 to October 27. In 2019, a similar plateau occurred during the same weeks, with weekly cases >200. Within each plateau, the weekly cases also varied, particularly the incidence drops in the weeks 29 and 30 in 2018 (July 15–28 with weekly 192–206 cases) and weeks 29–34 in 2019 (July 14 to August 24) and weeks 39–43 (September 22 to October 26, 2019). The weekly incidence rises and falls in 2018 and 2019 prompted regression analyses with the explanatory variables.

**Fig 6:**
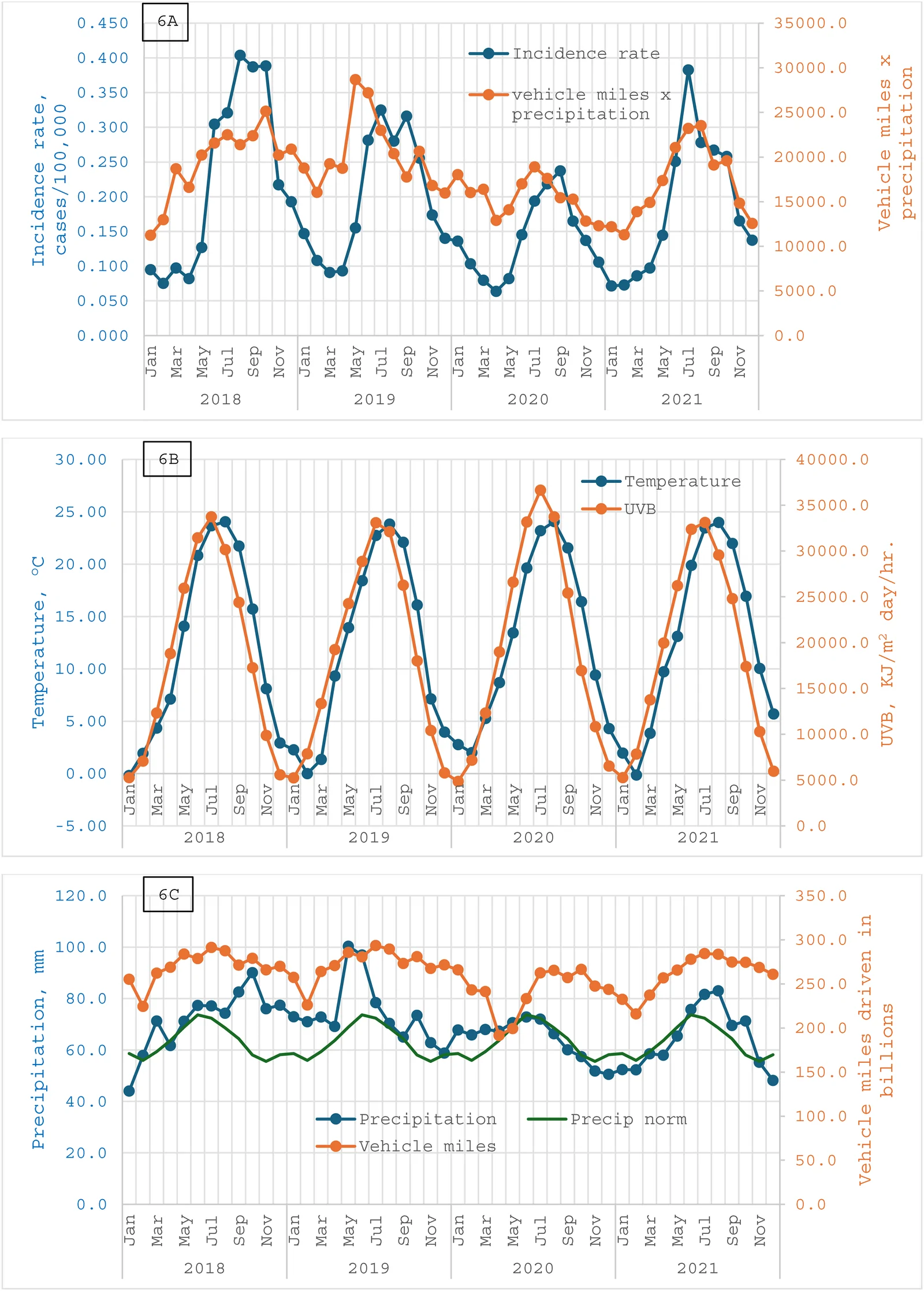
Monthly legionellosis incidence rates (cases/100,000) from 2018 to 2021 in relation to monthly vehicle miles driven x precipitation (6A), monthly mean temperature and ultraviolet B (6B), and monthly precipitation and vehicle miles driven (6C) in the United States. Y axis = parameter as shown; X axis = month and year.

The regression was performed between the Log-transformed monthly incidence rates and the available 14-days ahead adjusted monthly temperature, precipitation, vehicle miles, and UVB (Fig 6, Table 5, and S7 Table). In simple regressions, the temperature, precipitation, vehicle miles driven, and UVB each showed a promotive effect. In multiple regressions, robust correlations demonstrated strongly promotive effects of temperature, precipitation, and road exposure (the interactive precipitation x vehicle miles), but a strongly inhibitive effect of UVB. The mixed results of UVB were due to its multi-collinearity with temperature from solar effects. Thus, for the 2018 incidence plateau, the September and October cases were attributed to the large precipitation surplus and attenuation of the UVB. Conversely, the large precipitation surplus in May and June of 2019 was less effective due to the cool temperatures (13.9 to 18.4^o^C) but stronger UVB of the months. In the fall of 2019, less precipitation and stronger UVB also led to fewer cases. In both years, the intense UVB in July with near normal precipitation led to relatively fewer cases in July 2018 and August 2019. These monthly findings refined the annual data on the rise of incidence and main causes.

**Table 5:** Significant linear regressions between Log-transformed monthly legionellosis incidence rates (cases/100,000) and 14-day ahead adjusted monthly data of mean temperature, precipitation, ultraviolet B radiation, and vehicle miles driven in the United States, 2018-2021.

Regression and parameters	R^2^	Coefficient	*P* value	Interpretation
Before COVID, simple linear regressions, n = 24 months in 2018–2019				
Monthly mean temperature, °C	0.7075	0.023	2.6E-07	Strongly promotive
Monthly vehicle miles driven	0.4228	0.009	5.8E-04	Promotive
Monthly precipitation x vehicle miles	0.3826	3.8E-05	0.001	Promotive
Monthly precipitation, mm	0.2686	0.010	0.009	Promotive
*Monthly UVB radiation, KJ/m^2^ day/hr.	0.4012	1.5E-05	8.9E-04	Promotive
Before COVID, multiple linear regressions, n = 24 months in 2018–2019				
Adjusted R^2^ value	0.8899	--	2.3E-10	Robust correlation
Intercept		-1.124	1.3E-09	
Monthly mean temperature, °C		0.050	6.6E-09	Strongly promotive
Monthly precipitation, mm		0.0042	0.014	Promotive
Monthly UVB radiation, KJ/m^2^ day/hr.		-2.70E-05	6.5E-06	Strongly inhibitive
Adjusted R^2^ value	0.8893	--	2.4E-10	Robust correlation
Intercept		-1.092	2.4E-10	
Monthly mean temperature, °C		0.0492	1.1E-08	Strongly promotive
Monthly precipitation x vehicle miles		1.44E-05	0.015	Promotive
Monthly UVB radiation, KJ/m^2^ day/hr.		-2.79E-05	5.8E-06	Strongly inhibitive
During and before COVID, simple linear regressions, n = 48 months in 2018–2021				
Monthly mean temperature, °C	0.6418	0.022	8.0E-12	Strongly promotive
Monthly vehicle miles driven	0.5664	0.008	6.9E-10	Promotive
Monthly precipitation x vehicle miles	0.5065	4.08E-05	1.4E-08	Promotive
Monthly precipitation, mm	0.3117	0.011	3.7E-05	Promotive
*Monthly UVB radiation, KJ/m^2^ day/hr.	0.3454	1.35E-05	1.1E-05	Promotive
During and before COVID, multiple linear regressions, n = 48 months in 2018–2021				
Adjusted R^2^ value	0.8342		7.9E-18	Robust correlation
Intercept		-1.332	1.8E-19	
Monthly mean temperature, °C		0.0443	5.4E-14	Strongly promotive
Monthly precipitation		0.0065	3.1E-05	Strongly promotive
Monthly UVB radiation, KJ/m^2^ day/hr.		-2.4E-05	1.7E-08	Strongly inhibitive
Adjusted R^2^ value	0.8717		2.9E-20	Robust correlation
Intercept		-1.295	2.5E-25	
Monthly mean temperature, °C		0.0398	5.7E-14	Strongly promotive
Monthly precipitation x vehicle miles		2.35E-05	9.2E-08	Strongly promotive
Monthly UVB radiation, KJ/m^2^ day/hr.		-2.16E-05	4.4E-09	Strongly inhibitive

Explanatory parameters were adjusted for 14 days ahead in consideration of the disease incubation.

All data in S7 Table.

Additional regressions of the 48 months data using monthly cases were shown in S7 Table footnotes, including linear regressions, Poisson regressions, and negative binomial regressions. Normality tests for the explanatory variables were also performed and shown in the footnotes.

Strength of correlation: same as Table 3.

*In simple regression, monthly UVB showed a promotive effect due to closeness to temperature from the sunlight, contrasting to the strongly inhibitive effect in the multiple regressions. Temperature and UVB are inherently related, known as multi-collinearity [47]. UVB is minor radiation from the Sun and is better analyzed in conjunction with temperature.

Together, the emergence of seasonal incidence plateau was driven by the warmer winters since 1980 and more yearly warm days of ≥ 20^o^C during 1995–2021, particularly 2012–2021 (mean of 98 vs. norm 80 days, S3 Table). The immediate promotors were 7 successive years of precipitation surplus in 2013–2019 (+3.5 to 16.7% above norm, S4 Table) and 3 years of near record high temperature in 2015–2017 (+12.4 to 15.0% above norm, S4 Table). Warmer cold months every year raised the baseline bacterial density in the environment, which was further expanded in the ensuing warm months, with the latter becoming increasingly warmer and rainy to accelerate the process. Such yearly cycles led to cumulative effects. The vast vehicle miles driven in the US, averaging daily 8.2 billion in the cold January to 9.7 billion in the warm July (S7 Table), formed a constant basis for potential road exposure to the soil bacteria. The latter was influenced by the generation of aerosols on wet roads during or following precipitation, soil bacterial density, bacteria viability affected by the presence or absence of sunlight and its strength, and host vulnerability.

### Analyses of weekly and monthly legionellosis during COVID-19 in 2020 and 2021

The analyses of weekly and monthly legionellosis data during COVID-19 were also revealing.

The year 2020 started similarly to those in 2018 and 2019 (Fig 5B). The number of cases in the first 10 weeks (January 1 to March 7) represented cases prior to the COVID-19 lockdowns that started in most US states in mid-March, on consideration of ~2 weeks of incubation period of legionellosis. These weeks totaled 985 cases, 109% of the 2018 and 2019 average of 901 cases during the period. From week 11 on, the case numbers in the remaining 42 weeks were below those in 2018 and 2019 consistently, at 62.6% level on average, following the COVID-19 containment efforts – particularly the low vehicle miles during lockdowns and slow recovery thereafter. The seasonal incidence plateau was reduced to 58.7% of the average in the weeks 24–43 in 2018 and 2019, being lowest in weeks 25–27 (June 14 to July 4) with 362 cases (37.9% of 956) and week 42 (October 11–17) with 119 cases (44.1% of 270).

With the persistence of COVID-19 in 2021, partial containments continued, such as working from home, conscious restriction of non-essential travels, quarantine, etc. Thus, the cases in the first 13 weeks remained low. Thereafter, the weekly cases started to rise, along with return of the seasonal incidence plateau that resembled the one in 2019 in cases and contour. In 2021, the age group of ≥ 65 years made up 16.8% of the population but 44.9% of the legionellosis cases (3787 of 8442). By contrast, in 2000, this age group comprised 12.4% of the population and 38.3% of the cases (432 of 1127). The aging population thus contributed to more cases in recent years.

The weekly cases in 2020 and 2021 were also converted to monthly incidence rates and analyzed in regressions, together with the 2018 and 2019 monthly data. The regressions of the 48 monthly data replicated the robust correlations on the promotive effects of temperature and road exposure as well as the inhibitive effect of UVB (Fig 6, Table 5, and S7 Table). The role of road exposure became stronger, like the annual data. In addition to the monthly incidence rates, monthly cases were also subjected to Log-linear regressions, Poisson regressions, and negative binomial regressions. Nearly identical results were obtained, in reflection of normal distribution of the explanatory variables (S7 Table footnotes). These models predicted a mean difference of ~15% from the actual monthly cases or 85% accuracy (S8 Table).

Therefore, fewer vehicle miles and near normal precipitation during COVID in 2020, particularly in warm June to October, were the main causes of the drop of legionellosis. The recovery of vehicle miles and precipitation surplus in July to October 2021 contributed to the return of seasonal incidence plateau.

### State-wise analysis of the first incidence leap in 2003

The legionellosis incidence in the US rose steeply in 2003, from 0.46/100,000 in 2002 (1321 cases) to 0.75/100,000 (2232 cases) by 1.65-fold, to start the upward trend (Fig 3). The leap was noted first in several mid-Atlantic states and attributed to a large precipitation surplus in this region [13]. By the US average, however, the precipitation rose modestly from 2002 to 2003 by +5% (737.9 to 775.0 mm, norm 757.1 mm), after 4 successive years in deficit, while the annual mean temperatures were similar (11.78 vs 11.81^o^C, norm 11.07^o^C) (S4 Table). Prior to the leap, the temperature had been trending up for two decades (Table 1 footnote), yet with relatively stable annual cases of ~1200 [2,20]. In view of the wide incidence variation and diverse climate and geography across the US, state-wise analyses of the 2002–2003 changes in incidence, precipitation, and temperature were performed.

As shown in Table 6, the mid-Atlantic states all showed marked rises of annual cases (+84.2 to 223%, mean +130%) and precipitation (+23.0 to 77.1%, median +32.9%). The south and north Atlantic states, except cold Maine and Vermont, also experienced rises of cases (+7.6 to 81.8%, mean +27.1%) and precipitation (+0.5 to 29.2%, median +14.0%). Notably, the incidence leap in warm Florida (+72.9%) paced with the precipitation surplus in January to October (1236 mm in 2002 vs 1373 mm in 2003, + 11.1%), above the slight rise of annual precipitation (+0.5%). Together, 15 of the 17 Atlantic coastal states and jurisdictions (New York City and District of Columbia) showed parallel rises of precipitation and legionellosis incidence.

**Table 6:** State-wise analysis of the legionellosis cases in 2002 and 2003 with corresponding annual mean temperature and annual precipitation in the United States.

US region and State	Temperature ^o^ C				Precipitation mm				Legionellosis cases			
	2002	2003	+/-	%	2002	2003	+/-	%	2002	2003	+/-	%
Eastern Mid-Atlantic states												
Pennsylvania	10.2	8.9	-1.3	-12.6	1106	1361	255	23.0	158	291	133	84.2
New Jersey	12.4	11.0	-1.4	-11.2	1166	1435	268	23.0	35	94	59	168.6
Maryland	13.5	12.2	-1.3	-9.9	1053	1543	489	46.5	56	134	78	139.3
Delaware	14.0	12.6	-1.4	-10.3	1180	1469	288	24.4	6	19	13	216.7
Virginia	13.7	12.8	-0.9	-6.5	1083	1572	489	45.2	35	109	74	211.4
District of Columbia	15.3	13.9	-1.4	-9.1	873	1546	673	77.1	10	31	21	210.0
North Carolina	15.6	14.8	-0.7	-4.6	1207	1604	397	32.9	13	42	29	223.1
Eastern north and south Atlantic states												
New Hampshire	6.9	5.8	-1.1	-16.0	1140	1280	139	12.2	7	9	2	28.6
Massachusetts	9.6	8.4	-1.2	-12.7	1200	1354	154	12.8	45	57	12	26.7
Connecticut	10.3	9.0	-1.3	-12.4	1204	1425	221	18.3	19	28	9	47.4
Rhode Island	10.7	9.4	-1.2	-11.5	1205	1345	140	11.6	11	20	9	81.8
New York City	13.6	11.9	-1.7	-12.3	1149	1485	335	29.2	66	71	5	7.6
Georgia	18.0	17.4	-0.6	-3.4	1263	1454	191	15.1	19	34	15	78.9
Florida*	21.8	21.6	-0.2	-1.0	1486	1493	7	0.5	85	147	62	72.9
South Carolina	17.6	16.9	-0.7	-4.1	1182	1402	220	18.6	10	11	1	10.0
Vermont**	6.4	5.3	-1.2	-18.1	1200	1265	65	5.4	7	6	-1	-14.3
Maine	5.4	4.7	-0.7	-12.4	1041	1169	128	12.3	6	2	-4	-66.7
Other eastern states												
Ohio	11.4	10.1	-1.3	-11.2	980	1179	199	20.3	123	226	103	83.7
New York upstate	8.2	6.9	-1.3	-15.5	1107	1220	113	10.2	118	176	58	49.2
Alabama	17.6	17.1	-0.5	-2.8	1451	1618	167	11.5	8	20	12	150.0
Indiana	11.7	10.6	-1.1	-9.5	1072	1179	107	10.0	22	34	12	54.5
Michigan***	7.6	6.7	-0.9	-12.4	785	789	5	0.6	85	131	46	54.1
Illinois***	11.8	10.9	-0.8	-7.1	992	959	-33	-3.3	28	50	22	78.6
Missouri***	12.9	12.5	-0.4	-3.4	1021	1017	-5	-0.4	19	37	18	94.7
Kentucky***	13.8	12.9	-0.9	-6.5	1359	1422	63	4.6	22	46	24	109.1
Tennessee***	14.8	14.2	-0.7	-4.5	1504	1569	65	4.3	20	37	17	85.0
Arkansas***	15.9	15.7	-0.2	-1.0	1342	1139	-203	-15.1	0	2	2	>100
Louisiana	19.2	19.1	-0.1	-0.6	1632	1320	-313	-19.2	4	1	-3	-75.0
Minnesota	5.6	5.3	-0.3	-5.0	726	564	-162	-22.4	18	5	-13	-72.2
Wisconsin	7.0	6.2	-0.8	-11.1	891	712	-179	-20.1	38	18	-20	-52.6
Iowa	9.6	8.8	-0.7	-7.6	749	729	-20	-2.7	13	12	-1	-7.7
Mississippi	17.8	17.4	-0.4	-2.2	1678	1498	-180	-10.7	0	5	5	>100
West Virginia	11.8	10.9	-0.9	-7.5	1233	1509	276	22.4	0	26	26	Note
Subtotal of eastern states				-9.1				11.6	1134	1931	797	70.3
Western states												
California	14.9	15.2	0.3	1.9	474	549	75	15.8	60	77	17	28.3
Utah	9.3	10.3	1.0	10.7	230	293	63	27.5	16	27	11	68.8
Arizona	16.2	16.6	0.4	2.4	177	267	90	50.8	15	21	6	40.0
Oregon	8.7	9.4	0.7	8.3	666	814	149	22.3	5	17	12	240.0
Idaho	6.1	7.4	1.3	21.8	482	573	91	18.9	3	7	4	133.3
Nevada	10.4	11.2	0.8	7.4	158	249	91	57.4	6	12	6	100.0
Washington	8.3	9.0	0.7	8.7	927	1104	177	19.1	8	14	6	75.0
Colorado	7.8	8.3	0.5	6.4	301	418	117	38.7	9	12	3	33.3
Kansas	12.9	12.8	-0.1	-0.9	570	638	68	11.9	0	11	11	>100
Texas	18.3	18.6	0.3	1.5	779	615	-164	-21.0	28	71	43	153.6
Oklahoma	15.2	15.5	0.3	1.8	889	708	-181	-20.4	5	10	5	100.0
New Mexico	12.4	13.1	0.6	4.9	312	230	-82	-26.2	2	5	3	150.0
Montana	5.2	6.3	1.1	20.2	436	442	6	1.3	4	4	0	0.0
Wyoming	5.2	6.2	0.9	18.1	304	384	80	26.4	2	2	0	0.0
North Dakota	5.0	4.9	-0.1	-2.2	402	412	10	2.6	1	1	0	0.0
South Dakota	7.9	7.8	-0.1	-0.7	363	435	72	19.8	4	2	-2	-50.0
Nebraska	10.0	9.9	-0.1	-1.1	398	507	110	27.7	16	7	-9	-56.3
Subtotal of western states				4.9				19.1	184	300	116	63.0
US total	11.78	11.81	0.03	0.2	738	774	37	5.0	1318	2231	913	69.3
US cases/100,000 and change									0.46	0.75	0.29	65.0

*Florida showed significant precipitation surplus from January to October: 1236 mm in 2002 vs 1373 mm in 2003, up by 11.1%.

**A large outbreak of 28 cases occurred in Vermont in July 2002, according to CDC NORS [49]. These cases were not counted towards the state-wise analysis but towards US total and eastern states total to compensate for the lack of legionellosis report in West Virginia in 2002.

Note: West Virginia did not report legionellosis cases in 2002 and earlier, precluding accurate assessment.

***The states of Michigan, Illinois, Missouri, Kentucky, Tennessee, and Arkansas showed significant precipitation surplus in the months of June to November, up by 11.9% (420 mm in 2002 to 469 mm in 2003), 29.6% (433 to 561 mm), 39.2% (409 to 570 mm), 19.6% (624 to 746 mm), 7.9% (678 to 731 mm), and 20.3% (497 to 598 mm), respectively. In these states, most legionellosis cases occurred in the summer and fall.

The cases and populations of Alaska and Hawaii were only included in the final US cases/100,000 and change.

Calculations of change and %: change = (2003 value – 2002 value); % = change ÷ 2002 value x 100.

Color coding for easy recognition of the consistency or discrepancy in changes across rows and columns: green represented significant increase in temperature (> 4%), precipitation (> 7%), or cases (> 8% and > 1 case); black represented minor variation in temperature (within ± 4%), precipitation (within ± 7%), or cases (within ± 8% or 1 case); red represented significant decrease in temperature (< -4%), precipitation (< -7%), or cases (< -8% and < -1 case). The cutoff percentages were set around the coefficient of variation (CV) of the annual 1995-2021 values in Table 1. For example, the temperature CV = standard deviation ÷ mean x 100 = 0.505 ÷ 11.91 x 100 = 4.2%, the precipitation CV = 51.92 ÷ 792.8 x 100 = 6.6%.

Among the other 16 eastern states, four states showed parallel leaps of cases and annual precipitation (+10.0 to 20.3%) while four others (Minnesota, Wisconsin, Louisiana, and Iowa) exhibited parallel decreases of cases and precipitation. The marked decreases in cold Minnesota and Wisconsin were also aided by the decreases in temperature. Six states (Michigan, Illinois, Missouri, Kentucky, Tennessee, and Arkansas) also experienced rises of incidence and seasonal rainfall in the summer and fall (June to November, + 7.9 to 39.2%), despite slight variations or decrease in annual precipitation (+4.6 to –15.1%) (Table 6 footnotes). Together, fourteen of the 16 states showed parallel rises or decreases in cases and precipitation. This consistency could not be assessed in West Virginia due to the lack of legionellosis data in 2002 and earlier. Only Mississippi exhibited precipitation deficit but rose in cases from none to 5.

Together, in most eastern states and jurisdictions, marked precipitation surplus in 2003 brought up the legionellosis incidence, from 1134 cases in 2002 to 1931 cases in 2003 (+70.3% or 1.70-fold). The 1931 cases comprised 86.6% of the cases in the US in 2003. The precipitation surplus also damped the annual temperature in all 33 eastern states and jurisdictions, by -0.1 to -1.7^o^C (-0.6 to -18.1%, median -9.1%), which meant lack of correlation between the incidence and annual temperature, as also found in the regression analyses above (Table 3).

Among the 17 western states, twelve also showed rises in incidence whereas 5 states showed either no change (cold Montana, Wyoming, and North Dakota) or decrease (South Dakota and Nebraska). Nine of the 12 states exhibited parallel rises of incidence (+28.3 to 240%, mean +62.3%) and precipitation (+11.9 to 57.4%, median +22.3%). In addition, the temperature rose in 8 of the 9 states (+1.9 to 21.8%, median +7.8%), in contribution to the incidence rises. Only in 5 states (Texas, Oklahoma, New Mexico, South Dakota, and Nebraska), the changes in incidence and precipitation mismatched. Unlike the temperature drop in eastern states, thirteen western states showed temperature rises by +0.3 to 1.3^o^C (+1.5 to 21.8%), which resulted in similar annual temperatures in 2002 and 2003 in the US. Together, the number of legionellosis cases in the western states rose from 184 cases in 2002 to 300 cases in 2003 (+63.0%).

Therefore, by the state-wise analyses, the leap of the US legionellosis incidence in 2003 was attributed mainly to marked precipitation surplus in most states. The leap was led by the Atlantic coastal states, such as New Jersey, that had experienced two decades of warming temperature (Table 1 footnotes and S2 Table). In view of the strongly promotive effect of cumulative temperature anomaly (Table 3), the warmer temperature had likely raised the level of environmental bacteria to prime the leap. The massive vehicle mileage of 2890 billion miles in 2003, + 1.9% from 2836 billion miles in 2002 (Fig 3, S4 Table), also contributed to the leap in providing the traffic force to generate aerosols on rained road surfaces.

### State-wise analysis of the drought effect in 2012

Corresponding to the precipitation surplus in 8 of the 9 years from 2003 to 2011, the rising trend of the legionellosis incidence continued with only minor year-to-year variations. In 2012, the US experienced a severe drought (699.3 mm, -7.6% below norm, least since 1989) along with record high temperature (+16.8% above norm, since 1895). Accordingly, the incidence also dropped, from 1.27/100,000 (4202 cases) in 2011 to 1.09/100,000 (3688 cases) in 2012.

To examine the extent of drought effect across the US, state-wise analyses were performed (Table 7). All states showed temperature rises in 2012, by +0.1 to 2.5^o^C (+0.5 to 40.7%) above those in 2011, with the US mean + 1.2^o^C (+9.9%). The precipitation levels decreased in 35 states but increased in 14 states.

**Table 7:** State-wise analysis of the legionellosis cases in 2011 and 2012 with corresponding annual mean temperature and annual precipitation in the United States.

US region and State	Temperature ^o^ C				Precipitation mm				Legionellosis cases			
	2011	2012	+/-	%	2011	2012	+/-	%	2011	2012	+/-	%
Eastern states												
Pennsylvania	10.2	11.0	0.8	7.6	1549	1105	-445	-28.7	502	300	-202	-40.2
Ohio	11.3	12.2	0.9	8.4	1421	946	-475	-33.4	386	288	-98	-25.4
New York upstate	8.4	9.3	0.9	11.3	1415	1005	-410	-29.0	400	325	-75	-18.8
New Jersey	12.7	13.3	0.6	4.4	1624	1067	-558	-34.3	235	173	-62	-26.4
New York City	13.6	14.1	0.5	3.7	1850	979	-871	-47.1	216	177	-39	-18.1
Maryland	13.6	14.2	0.6	4.1	1298	1026	-272	-21.0	143	123	-20	-14.0
Massachusetts	9.8	10.7	0.9	9.0	1545	1061	-484	-31.3	240	173	-67	-27.9
Connecticut	10.5	11.4	0.9	8.5	1618	1001	-617	-38.2	81	55	-26	-32.1
New Hampshire	7.2	8.1	0.9	13.2	1460	1148	-313	-21.4	26	19	-7	-26.9
Illinois*	11.8	13.2	1.4	12.3	1171	765	-406	-34.7	151	112*	-39	-25.8
Indiana	11.7	12.8	1.1	9.5	1402	859	-543	-38.7	71	54	-17	-23.9
Delaware	14.1	14.7	0.6	4.3	1132	999	-133	-11.8	24	17	-7	-29.2
Tennessee	14.9	15.7	0.8	5.2	1590	1191	-399	-25.1	84	57	-27	-32.1
Kentucky	13.8	14.7	0.8	6.0	1634	1098	-537	-32.9	53	43	-10	-18.9
Virginia	13.9	14.2	0.3	2.4	1227	1017	-210	-17.1	93	76	-17	-18.3
Michigan	7.6	9.1	1.6	20.6	935	787	-148	-15.8	187	178	-9	-4.8
North Carolina	15.8	15.9	0.2	1.1	1168	1184	16	1.4	83	65	-18	-21.7
Alabama	17.7	18.3	0.6	3.5	1288	1356	68	5.3	29	20	-9	-31.0
Florida	22.1	22.2	0.1	0.5	1188	1399	211	17.8	185	213	28	15.1
Louisiana	19.8	20.4	0.6	2.8	1092	1597	505	46.2	25	29	4	16.0
Mississippi	18.1	18.7	0.7	3.7	1342	1503	161	12.0	14	17	3	21.4
Minnesota	5.6	7.3	1.8	32.0	618	665	46	7.5	29	51	22	75.9
Wisconsin	6.7	8.6	1.8	27.3	788	732	-57	-7.2	69	101	32	46.4
West Virginia	11.9	12.4	0.4	3.7	1400	1133	-266	-19.0	32	37	5	15.6
Iowa	9.1	11.2	2.1	22.6	808	650	-158	-19.6	11	13	2	18.2
Missouri	13.1	14.8	1.7	12.7	1161	788	-373	-32.1	55	68	13	23.6
Arkansas	16.6	17.6	1.0	6.0	1418	1011	-407	-28.7	14	20	6	42.9
South Carolina	17.8	18.1	0.2	1.2	971	1112	141	14.6	25	26	1	4.0
Georgia	18.1	18.6	0.5	2.8	1019	1067	49	4.8	55	56	1	1.8
Rhode Island	10.9	11.6	0.7	6.6	1461	1045	-417	-28.5	29	31	2	6.9
Maine	6.0	6.6	0.6	10.2	1271	1203	-68	-5.3	18	18	0	0.0
Vermont	6.8	7.7	0.9	13.0	1479	1076	-403	-27.3	12	12	0	0.0
Subtotal of eastern states				6.6				-21.2	3577	2947	-630	-17.6
Western states												
Texas	19.6	19.9	0.3	1.4	373	612	239	63.9	111	158	47	42.3
Oklahoma	16.4	17.3	0.9	5.4	666	653	-14	-2.1	15	22	7	46.7
Nevada	9.8	11.3	1.6	15.9	219	226	7	3.1	16	18	2	12.5
Utah	8.7	10.5	1.8	21.2	374	272	-102	-27.3	18	27	9	50.0
Kansas	12.9	14.6	1.7	12.9	614	490	-124	-20.2	14	16	2	14.3
Nebraska	9.3	11.5	2.2	23.2	656	339	-316	-48.2	8	11	3	37.5
South Dakota	7.1	9.6	2.5	35.2	562	370	-192	-34.1	2	9	7	350
Montana	5.3	6.9	1.6	30.2	580	427	-153	-26.4	1	4	3	300
Wyoming	5.1	7.1	2.1	40.7	471	278	-193	-40.9	4	4	0	0.0
North Dakota	4.8	6.7	1.9	39.1	545	383	-161	-29.6	3	3	0	0.0
Oregon	7.9	8.9	0.9	11.9	779	1018	239	30.6	22	22	0	0.0
Arizona	15.7	16.7	1.0	6.4	245	243	-2	-0.7	46	44	-2	-4.3
Idaho	5.8	7.3	1.5	26.0	627	653	26	4.1	9	5	-4	-44.4
California	14.1	15.3	1.2	8.3	477	596	118	24.8	261	219	-42	-16.1
Washington	7.5	8.4	0.9	11.9	1113	1343	230	20.7	37	27	-10	-27.0
Colorado	7.4	9.1	1.6	21.6	448	313	-135	-30.1	41	24	-17	-41.5
New Mexico	12.6	13.3	0.8	6.2	249	214	-35	-14.2	12	9	-3	-25.0
Subtotal of western states				15.9				-14.2	620	622	2	0.3
US total	11.76	12.93	1.17	9.9	764	699	-65	-8.5	4197	3683	-514	-12.2
US cases/100,000 and change									1.27	1.09	-0.18	-14.2

*A major outbreak of 114 cases occurred in Illinois in 2012 due to the contamination of a hotel fountain [6], which were not counted in the analysis of Illinois or eastern states but included in the US total.

The cases and populations of Alaska and Hawaii were only included in the final US cases/100,000 and change.

The District of Columbia did not report cases in 2011 or 2012.

Calculations of change and %: like Table 6.

Color coding: same as Table 6.

In the 32 eastern states and jurisdiction (New Your City), sixteen showed parallel drops of cases (-4.8 to -40.2%, mean -25.0%) and precipitation (-11.8 to -47.1%, median -30.2%). Two states (North Carolina and Alabama) also showed drops of incidence whereas the precipitation levels varied slightly. Four states (Florida, Louisiana, Mississippi, and Minnesota) experienced parallel rises of incidence and precipitation; in particular, the dual rises of precipitation (+7.5%) and temperature (+32.0%) contributed to the incidence hike in cold Minnesota (+75.9%). Similarly, in the nearby cold Wisconsin, the incidence hike (+46.4%) could be explained by the rise of temperature (+27.3%) despite the decrease of precipitation (-7.2%). The remaining 9 eastern states, all with relatively low incidences, exhibited slight to considerable rises of cases in 7 states and no change in 2 states, along with mixed changes of precipitation and temperature. Together, in most eastern states, precipitation deficits led to the drop of legionellosis incidence, from 3577 cases in 2011 to 2947 cases in 2012 (-17.6%).

Unlike the eastern states, the incidence in the western states varied little overall (+0.3%), being mixed with increase in 8 states, no change in 3 states, and decrease in 6 states. Significant parallel changes were seen in only three states, i.e., drops of incidence and precipitation in Colorado and New Mexico but rises of both in Texas. Notably, Texas experienced a record drought in 2011 [49,50]; with the return of precipitation in 2012 (+63.9%), the incidence rebounded (+42.3%). Most other states showed mixed changes. Therefore, the incidence drop in 2012 in the US was mainly due to the severe widespread drought, not record high temperature.

### State-wise analysis of the peak incidence in 2018

The incidence peaked in 2018 upon 16 years of remarkable rises from 2003. To examine the consistency and variation across the US, state-wise analyses were again performed (Table 8). Among the 33 eastern states and jurisdictions, 28 showed rises of incidence with parallel precipitation surplus in 27 states except Michigan. The 27 states averaged rises of cases by +43.2% (+8.7 to 163%) and precipitation by +23.9% (in median, + 0.3 to 87.3%). Only five states experienced decreases in incidence, despite precipitation surpluses. The rises of precipitation also led to drops of temperature by -0.5 to –18.9% (median –3.2%) in 31 states, except slight increases in 2 states. Together, the eastern states suffered +41.4% incidence hike, from 5996 cases in 2017 to 8479 cases in 2018, the highest proportion since the 2003 leap of +70.3%. In most states, the parallel rises of incidence and precipitation in 2018 and 2003 were similar.

**Table 8:** State-wise analysis of the legionellosis cases in 2017 and 2018 with corresponding annual mean temperature and annual precipitation in the United States.

US region and State	Temperature ^o^ C				Precipitation mm				Legionellosis cases			
	2017	2018	+/-	%	2017	2018	+/-	%	2017	2018	+/-	%
Eastern states												
Ohio	11.8	11.2	-0.6	-4.7	1156	1294	138	11.9	601	930	329	54.7
Pennsylvania	10.3	9.9	-0.4	-4.3	1184	1627	443	37.4	500	638	138	27.6
New York City	13.6	13.3	-0.3	-2.0	1145	1665	520	45.5	435	654	219	50.3
New Jersey	12.6	12.3	-0.2	-1.8	1141	1645	504	44.2	249	369	120	48.2
Maryland	13.7	13.3	-0.4	-3.2	1047	1641	595	56.8	187	361	174	93.0
Massachusetts	9.8	9.7	-0.1	-0.6	1189	1550	361	30.4	201	368	167	83.1
Connecticut	10.5	10.4	-0.1	-0.5	1120	1579	459	41.0	120	198	78	65.0
Rhode Island	10.8	10.9	0.1	0.5	1247	1605	357	28.6	50	73	23	46.0
West Virginia	12.2	11.8	-0.4	-3.2	1194	1654	459	38.5	49	129	80	163.3
Delaware	14.2	13.9	-0.3	-2.0	1131	1525	393	34.8	34	46	12	35.3
Virginia	14.1	13.7	-0.4	-2.8	1056	1614	558	52.9	197	236	39	19.8
District of Columbia	16.0	13.4	-2.6	-16.0	905	1695	790	87.3	46	57	11	23.9
South Carolina	18.4	17.9	-0.4	-2.4	1183	1455	273	23.0	55	63	8	14.5
Georgia	18.8	18.4	-0.4	-2.1	1247	1544	298	23.9	159	181	22	13.8
Illinois	12.4	11.4	-1.1	-8.5	960	1163	203	21.1	332	509	177	53.3
Indiana	12.2	11.4	-0.8	-6.4	1158	1271	112	9.7	198	249	51	25.8
Iowa	10.0	8.6	-1.4	-13.9	822	1145	323	39.4	35	63	28	80.0
Tennessee*	15.6	15.1	-0.5	-3.2	1428	1705	277	19.4	115*	171	56	48.7
Kentucky	14.3	13.9	-0.4	-3.1	1277	1619	342	26.8	116	146	30	25.9
Minnesota**	5.9	4.8	-1.1	-18.9	719	764	45	6.3	98	152	54	55.1
Wisconsin**	7.1	6.2	-0.8	-11.8	954	1009	55	5.8	176	331	155	88.1
Missouri**	14.0	12.9	-1.1	-7.9	1053	1092	39	3.7	138	150	12	8.7
New Hampshire**	7.2	6.9	-0.3	-3.9	1249	1316	67	5.3	63	77	14	22.2
Maine**	5.8	5.4	-0.3	-5.8	1151	1180	29	2.5	16	34	18	112.5
New York upstate**	8.3	7.9	-0.4	-4.7	1203	1234	30	2.5	587	770	183	31.2
Florida	22.7	22.4	-0.3	-1.2	1489	1537	48	3.2	435	496	61	14.0
Alabama	18.3	18.6	0.2	1.2	1630	1636	5	0.3	66	76	10	15.2
Michigan	7.9	7.3	-0.7	-8.4	997	919	-79	-7.9	347	633	286	82.4
Louisiana	20.4	19.9	-0.5	-2.5	1627	1686	59	3.6	49	42	-7	-14.3
North Carolina	16.1	15.8	-0.3	-2.1	1218	1736	518	42.5	212	173	-39	-18.4
Mississippi	18.8	18.2	-0.6	-3.0	1497	1697	200	13.4	52	41	-11	-21.2
Arkansas	16.9	16.2	-0.7	-4.3	1277	1634	357	28.0	56	46	-10	-17.9
Vermont	6.6	6.2	-0.3	-5.1	1176	1196	20	1.7	22	17	-5	-22.7
Subtotal of eastern states				-3.2				32.0	5996	8479	2483	41.4
Western states												
Texas	19.6	18.9	-0.7	-3.7	769	856	87	11.4	327	415	88	26.9
Arizona	17.2	16.8	-0.4	-2.3	241	296	56	23.2	74	83	9	12.2
South Dakota	8.2	6.7	-1.5	-18.2	448	591	143	31.9	15	33	18	120.0
Nebraska	10.4	9.1	-1.3	-12.8	644	742	99	15.3	29	43	14	48.3
North Dakota	5.6	4.3	-1.2	-22.0	330	451	121	36.8	7	10	3	42.9
Kansas	13.6	12.6	-1.1	-7.8	737	809	72	9.7	34	41	7	20.6
Colorado	8.8	8.4	-0.4	-4.4	480	380	-100	-20.9	82	110	28	34.1
New Mexico	13.6	13.1	-0.5	-3.7	376	323	-53	-14.1	13	22	9	69.2
Idaho	6.8	6.9	0.1	1.6	732	556	-177	-24.1	12	15	3	25.0
Nevada	11.2	11.1	-0.1	-1.0	284	235	-48	-17.0	14	16	2	14.3
Utah	10.4	10.2	-0.2	-1.6	326	294	-32	-9.7	32	33	1	3.1
Oklahoma	16.6	15.7	-0.9	-5.4	981	1042	61	6.2	65	63	-2	-3.1
Washington	8.2	8.9	0.7	8.1	1238	1050	-188	-15.2	56	54	-2	-3.6
California	15.7	15.6	-0.1	-0.7	716	459	-256	-35.8	535	453	-82	-15.3
Oregon	8.9	9.1	0.2	2.5	920	645	-275	-29.8	37	31	-6	-16.2
Wyoming	6.2	5.8	-0.4	-7.1	474	409	-65	-13.6	7	2	-5	-71.4
Montana	6.2	5.2	-1.1	-17.0	443	507	65	14.6	17	10	-7	-41.2
Subtotal of western states				-3.7				-9.7	1356	1434	78	5.8
US total	12.52	11.96	-0.57	-4.5	820	880	60	7.3	7444	9913	2469	33.2
US cases/100,000 and change									2.04	2.69	0.65	31.9

*A major outbreak of 92 cases occurred in 2017 at a Tennessee hotel according to CDC NORS [51,52], which were not counted in the analysis of Tennessee or eastern states but included in the US total.

**The states of Minnesota, Wisconsin, Missouri, New Hampshire, Maine, New York upstate, also experienced significant precipitation surplus in the months of June to November, by 15.4% (486 to 561 mm), 26.5% (549 to 694 mm), 22.7% (472 to 579 mm), 23.6% (651 to 804 mm), 15.0% (560 to 644 mm), and 19.7% (617 to 738 mm), respectively. In these states, most legionellosis cases occurred in the summer and fall.

The cases and populations of Alaska and Hawaii were only included in the final US cases/100,000 and change.

Calculations of change and %: like Table 6.

Color coding: same as Table 6.

Among the 17 western states, the incidences rose in 11 states but decreased in 6, resulting in an overall increase of cases by +5.8%. Six states showed parallel rises of cases and precipitation whereas all other states did not. Decreases in temperature were seen in 14 states, by -0.7 to -22.0%. Therefore, the peaking of the US legionellosis incidence in 2018 was attributed to significant precipitation surplus in most states that averaged near record level in the US (880.1 mm, + 16.2% above norm). The vehicle mileage increased slightly from 2017 to 2018, by 0.9% (3212 to 3240 billion miles, S4 Table).

### State-wise analysis of the incidence variation in 2019

The incidence in 2019 dropped from the peak in 2018, despite similar precipitation level (883.4 mm, 16.7% above norm), which led to state-wise analyses (Table 9).

**Table 9:** State-wise analysis of the legionellosis cases in 2018 and 2019 with corresponding annual mean temperature and annual precipitation in the United States.

US region and State	Temperature ^o^ C				Precipitation mm				Legionellosis cases			
	2018	2019	+/-	%	2018	2019	+/-	%	2018	2019	+/-	%
Cold eastern states*												
Minnesota	4.8	4.1	-0.7	-15.1	764	906	142	18.6	152	118	-34	-22.4
Maine	5.4	4.8	-0.6	-11.2	1180	1270	90	7.6	34	30	-4	-11.8
Vermont	6.2	5.5	-0.7	-11.6	1196	1306	110	9.2	17	12	-5	-29.4
Wisconsin	6.2	5.6	-0.6	-9.8	1009	1126	117	11.6	331	232	-99	-29.9
New Hampshire	6.9	6.2	-0.7	-9.7	1316	1276	-39	-3.0	77	32	-45	-58.4
Michigan	7.3	6.6	-0.7	-9.2	919	1061	143	15.5	633	551	-82	-13.0
New York upstate	7.9	7.4	-0.5	-6.3	1234	1225	-9	-0.7	770	606	-164	-21.3
Warm eastern states												
Connecticut	10.4	9.9	-0.6	-5.3	1579	1369	-210	-13.3	198	119	-79	-39.9
Rhode Island	10.9	10.3	-0.6	-5.1	1605	1451	-154	-9.6	73	48	-25	-34.2
Massachusetts	9.7	9.2	-0.6	-5.7	1550	1339	-211	-13.6	368	247	-121	-32.9
West Virginia	11.8	12.3	0.5	4.2	1654	1225	-428	-25.9	129	82	-47	-36.4
New York City	13.3	13.1	-0.2	-1.3	1665	1348	-318	-19.1	654	446	-208	-31.8
District of Columbia	13.4	13.8	0.4	2.9	1695	1038	-658	-38.8	57	41	-16	-28.1
Maryland	13.3	13.7	0.4	2.9	1641	1085	-557	-33.9	361	273	-88	-24.4
Delaware	13.9	14.2	0.2	1.6	1525	1042	-483	-31.7	46	35	-11	-23.9
Virginia	13.7	14.2	0.5	3.7	1614	1168	-445	-27.6	236	191	-45	-19.1
Ohio	11.2	11.3	0.1	1.0	1294	1190	-103	-8.0	930	785	-145	-15.6
New Jersey	12.3	12.4	0.1	0.5	1645	1319	-326	-19.8	369	318	-51	-13.8
Florida	22.4	22.9	0.5	2.2	1537	1322	-215	-14.0	496	448	-48	-9.7
Pennsylvania	9.9	9.9	0.1	0.6	1627	1289	-338	-20.8	638	579	-59	-9.2
Kentucky**	13.9	14.2	0.3	2.0	1619	1558	-61	-3.8	146	121	-25	-17.1
Tennessee**	15.1	15.4	0.4	2.6	1705	1700	-6	-0.3	171	149	-22	-12.9
Georgia	18.4	19.0	0.6	3.3	1544	1216	-328	-21.2	181	171	-10	-5.5
Alabama	18.6	18.1	-0.5	-2.7	1636	1430	-206	-12.6	76	72	-4	-5.3
North Carolina***	15.8	16.3	0.5	3.2	1736	1299	-437	-25.2	173	169*	-4	-2.3
Indiana	11.4	11.3	-0.1	-1.0	1271	1258	-13	-1.0	249	263	14	5.6
South Carolina	17.9	18.3	0.4	2.2	1455	1169	-286	-19.7	63	67	4	6.3
Iowa	8.6	8.2	-0.4	-4.5	1145	1054	-91	-8.0	63	68	5	7.9
Louisiana	19.9	19.8	-0.1	-0.3	1686	1571	-115	-6.8	42	50	8	19.0
Mississippi**	18.2	18.4	0.2	1.2	1697	1729	32	1.9	41	47	6	14.6
Illinois	11.4	11.2	-0.2	-2.0	1163	1266	103	8.9	509	614	105	20.6
Missouri	12.9	12.8	-0.1	-0.9	1092	1366	274	25.1	150	183	33	22.0
Arkansas**	16.2	16.2	0.0	0.0	1634	1668	34	2.1	46	66	20	43.5
Subtotal of eastern states				-0.3				-8.0	8479	7233	-1246	-14.7
Western states												
Nevada	11.1	9.9	-1.2	-10.5	235	351	115	49.0	16	25	9	56.3
Utah	10.2	8.8	-1.4	-13.6	294	430	136	46.3	33	38	5	15.2
Arizona	16.8	15.7	-1.1	-6.6	296	373	77	25.9	83	93	10	12.0
Oregon	9.1	8.4	-0.7	-7.9	645	763	117	18.2	31	55	24	77.4
Kansas	12.6	12.1	-0.4	-3.5	809	915	106	13.1	41	66	25	61.0
Montana	5.2	4.4	-0.8	-15.1	507	544	36	7.2	10	14	4	40.0
Idaho	6.9	6.1	-0.8	-12.0	556	595	39	7.0	15	25	10	66.7
Nebraska	9.1	8.6	-0.4	-4.9	742	797	55	7.4	43	46	3	7.0
Wyoming	5.8	4.6	-1.2	-20.2	409	466	56	13.8	2	3	1	50.0
California	15.6	14.7	-0.9	-6.0	459	564	105	22.8	453	451	-2	-0.4
New Mexico	13.1	12.4	-0.6	-4.7	323	340	17	5.2	22	21	-1	-4.5
Texas	18.9	18.8	-0.1	-0.3	856	681	-175	-20.4	415	421	6	1.4
North Dakota	4.3	3.3	-1.0	-23.1	451	620	168	37.3	10	9	-1	-10.0
South Dakota	6.7	5.7	-1.0	-14.9	591	798	207	35.1	33	23	-10	-30.3
Colorado	8.4	7.4	-1.1	-12.5	380	480	100	26.4	110	90	-20	-18.2
Oklahoma	15.7	15.5	-0.2	-1.1	1042	1142	101	9.7	63	52	-11	-17.5
Washington	8.9	8.1	-0.8	-9.4	1050	840	-210	-20.0	54	76	22	40.7
Subtotal of western states				-9.4				13.8	1434	1508	74	5.2
US total	11.96	11.49	-0.47	-3.9	880	883	3	0.4	9913	8877	-10.5	-10.5
US cases/100,000 and change									2.69	2.37	-0.32	-11.9

*The states of Minnesota, Maine, Vermont, Wisconsin, New Hampshire, Michigan, and New York upstate all experienced temperature drops from 2016, the nearest peak year, to 2019 for 3 years, by –42.1% (7.0 to 4.1^o^C), –23.0% (6.3 to 4.8^o^C), –22.0% (7.1 to 5.5^o^C), –28.9% (7.9 to 5.6^o^C), –18.2% (7.6 to 6.2^o^C), –22.7% (8.6 to 6.6^o^C), and –15.3% (8.7 to 7.4^o^C), respectively.

**Kentucky and Tennessee showed precipitation deficits in May to October, -11.1% (793 to 706 mm) and -4.5% (746 to 713 mm), respectively.

** Mississippi and Arkansas showed precipitation surpluses in May to October, +31.7% (655 to 862 mm) and +29.3% (671 to 868 mm), respectively.

***A major outbreak of 136 cases occurred in 2019 in North Carolina due to contaminated hot tubs [4]; these cases were not counted in the analysis of this state but included in the US total.

The cases and populations of Alaska and Hawaii were only included in the final US cases/100,000 and change.

Calculations of change and %: like Table 6.

Color coding: same as Table 6.

Among the 33 eastern states and jurisdictions, 25 showed drops of incidence while 8 states showed rises. Eighteen of the 25 states exhibited usual parallel drops of incidence (mean -19.0%) and precipitation (median -19.4%); however, the other seven, all cold and northern, showed unusual parallel drops of incidence (mean -21.5%) and temperature (median -9.8%), despite precipitation surpluses or minor variation (median +9.2%). In these 7 states, the annual temperatures had declined from 2016, the nearest peak year, to 2019 for 3 years, in sum by -15.3 to –42.1% (median –22.7%) (Table 9 footnotes). For instance, in the coldest Minnesota, they decreased from 7.0^o^C in 2016, to 5.9^o^C in 2017, 4.8^o^C in 2018, and 4.1^o^C in 2019 (below norm of 4.4^o^C), together with -2.9^o^C (-42.1%). Therefore, in the cold eastern states, the annual temperature could also be influential on the legionellosis incidence.

Five eastern states, all in the mid- to lower Mississippi river valley, showed significant rises of incidence (mean +21.8%) with parallel precipitation surplus or minor variation (median +8.9%). The increase was most notable in Illinois, a central state with incidence peak in 2019, instead of 2018 in most other eastern states. Warming temperatures in Illinois trended up in 1997, ~ 15 years behind the coastal states (S2 Table and S1 Fig). During 1999–2019, 17 of the 21 years had precipitation surplus, including near record levels in 2009, 2008 and 2019 (1294.3 to 1266.4 mm, + 36.6 to 33.6% above norm) [41]. Consequently, the legionellosis cases in Illinois rose markedly during the 21 years, from 33 cases in 1999, to 135 in 2009, 332 in 2017, and 614 in 2019. Overall, the eastern states showed incidence decrease in 2019, by -14.7% below that in 2018, owing to less precipitation surplus and/or lower temperature in most states.

The western states, on the other hand, showed more precipitation surpluses in 2019 than in 2018 in 15 of the 17 states. As such, nine western states showed parallel rises of incidence. Despite less precipitation, Washington also saw more cases (+40.7%). The 7 states with less cases or minor variation did not show consistent correlation with precipitation. Together, the western states showed incidence increase in 2019, by +5.2% above that in 2018, coinciding with more precipitation in most states. The annual temperatures dropped across the western states, unlike many eastern states with minor variation.

Therefore, across the US, regional changes in precipitation level and/or temperature were responsible for much of the incidence variation in 2019. In warm states, the precipitation levels generally paralleled legionellosis incidence while, in cold states, the temperature effect was dominant. The vehicle miles increased slightly from 2018 to 2019, by +0.7% (3240 to 3262 billion miles, S4 Table).

### State-wise analyses of the motor vehicle traffic effects in 2020 and 2021 during COVID-19

The state-wise analyses above on the incidence rises or variations in representative pre-COVID years from 2003 to 2019 established the consistency of dominant effects of annual precipitation across the US, particularly the eastern states. The consistency facilitated state-wise analyses of the effects of motor vehicle traffic in 2020 and 2021 during COVID-19.

As shown in Table 10, the reduction of vehicle miles driven in 2020 (mean -11.0%) paralleled the incidence drops in most states (mean -29.1%). The most incidence drops were seen in 20 eastern states (mean -33.7%) that also experienced precipitation deficits (-1.8 to -31.6%, median -16.4%). The least drops occurred in 12 eastern states (mean -17.2%) with precipitation surpluses (+1.1 to 40.4%, median +25.0%). Thus, the effect of precipitation was significant (χ^2^ = 115.3, *P* < 0.001, Table 10 footnotes). The 17 western states showed consistent incidence drops (mean -21.3%) and precipitation deficits (median -36.9%, except Washington).

**Table 10:** State-wise analysis of the legionellosis cases in 2019 and 2020 with corresponding changes in annual mean temperature, annual precipitation, and vehicle miles traveled in the United States.

US region and State	Tm ^o^ C	Precipitation mm				Vehicle miles in billion				Legionellosis cases			
	+/- %	2019	2020	+/-	%	2019	2020	+/-	%	2019	2020	+/-	%
Eastern states with precipitation deficit													
Minnesota	42.5	906	620	-286	-31.6	60.7	51.6	-9.11	-15.0	118	94	-24	-20.3
Iowa	16.2	1054	744	-310	-29.4	33.5	29.8	-3.79	-11.3	68	34	-34	-50.0
Wisconsin*	26.7	1126	868	-258	-22.9	66.3	57.6	-8.75	-13.2	232	211	-21	-9.1
Rhode Island	11.8	1451	1131	-320	-22.0	7.6	6.9	-0.72	-9.5	48	36	-12	-25.0
Vermont	29.3	1306	1021	-284	-21.8	7.3	6.0	-1.34	-18.2	12	8	-4	-33.3
Massachusetts	14.5	1339	1077	-262	-19.6	64.9	54.1	-10.76	-16.6	247	139	-108	-43.7
Connecticut**	11.2	1369	1102	-268	-19.6	31.6	29.8	-1.76	-5.6	119	82	-37	-31.1
Maine	32.2	1270	1032	-238	-18.7	14.9	13.1	-1.79	-12.0	30	11	-19	-63.3
New York	18.8	1225	998	-227	-18.5	124.0	102.5	-21.51	-17.3	1052	693	-359	-34.1
New Hampshire	24.1	1276	1062	-215	-16.8	13.8	12.0	-1.87	-13.5	32	32	0	0.0
Illinois	6.0	1266	1065	-201	-15.9	107.5	94.1	-13.40	-12.5	614	329	-285	-46.4
Michigan	21.8	1061	897	-165	-15.5	102.2	86.5	-15.63	-15.3	551	378	-173	-31.4
Pennsylvania	7.3	1289	1099	-190	-14.7	102.9	88.0	-14.88	-14.5	579	351	-228	-39.4
Indiana	4.4	1258	1077	-181	-14.4	82.7	76.6	-6.11	-7.4	263	155	-108	-41.1
Missouri	3.5	1366	1177	-189	-13.8	79.2	72.8	-6.37	-8.0	183	108	-75	-41.0
Ohio	2.9	1190	1075	-116	-9.7	114.7	103.1	-11.58	-10.1	785	537	-248	-31.6
Kentucky	-1.6	1558	1476	-82	-5.2	49.4	46.5	-2.87	-5.8	121	74	-47	-38.8
New Jersey	5.4	1319	1266	-53	-4.0	78.2	66.3	-11.86	-15.2	318	241	-77	-24.2
Arkansas	0.0	1668	1621	-46	-2.8	37.1	33.9	-3.18	-8.6	66	39	-27	-40.9
Tennessee	-1.8	1700	1670	-30	-1.8	82.9	76.4	-6.50	-7.8	149	154	5	3.4
Subtotal	11.5				-16.4	1262	1108	-153.8	-12.2	5587	3706	-1881	-33.7
Eastern states with precipitation surplus													
Mississippi	0.9	1729	1749	20	1.1	41.1	39.7	-1.43	-3.5	47	33	-14	-29.8
Louisiana	1.4	1571	1701	129	8.2	51.4	48.4	-2.99	-5.8	50	52	2	4.0
Florida	0.0	1322	1473	151	11.4	226.5	208.1	-18.44	-8.1	448	428	-20	-4.5
West Virginia	-0.9	1225	1366	140	11.5	19.1	16.1	-3.02	-15.8	82	49	-33	-40.2
Alabama	1.2	1430	1744	314	22.0	71.7	67.9	-3.81	-5.3	72	61	-11	-15.3
Maryland	2.4	1085	1342	258	23.8	60.2	50.9	-9.33	-15.5	273	182	-91	-33.3
Georgia	-1.8	1216	1536	319	26.3	133.1	116.0	-17.16	-12.9	171	145	-26	-15.2
South Carolina	-1.2	1169	1521	352	30.1	57.9	54.0	-3.97	-6.8	67	57	-10	-14.9
North Carolina***	-1.7	1299	1690	391	30.1	122.5	106.3	-16.13	-13.2	169	130	-39	-23.1
Virginia	-0.4	1168	1560	391	33.5	85.4	76.1	-9.32	-10.9	191	205	14	7.3
Delaware	2.0	1042	1420	378	36.3	10.2	8.3	-1.90	-18.5	35	14	-21	-60.0
District of Columbia	1.6	1038	1457	420	40.4	3.8	3.0	-0.73	-19.3	41	7	-34	-82.9
Subtotal	0.5				25.0	883.0	794.7	-88.2	-10.0	1646	1363	-283	-17.2
Eastern states total	3.2				-7.5	2144	1902	-242.0	-11.3	7233	5069	-2164	-29.9
Western states													
Nevada	14.0	351	149	-202	-57.6	28.8	25.2	-3.56	-12.4	25	26	1	4.0
Utah	15.7	430	184	-246	-57.3	32.9	30.3	-2.66	-8.1	38	32	-6	-15.8
Arizona	8.1	373	167	-207	-55.3	70.3	65.8	-4.52	-6.4	93	90	-3	-3.2
South Dakota	43.7	798	411	-387	-48.5	9.9	9.7	-0.18	-1.8	23	10	-13	-56.5
North Dakota	71.7	620	328	-291	-47.0	9.8	8.8	-1.06	-10.8	9	6	-3	-33.3
California	8.0	564	307	-258	-45.7	340.8	299.8	-41.02	-12.0	451	380	-71	-15.7
Nebraska	20.0	797	463	-334	-41.9	21.2	19.4	-1.81	-8.5	46	16	-30	-65.2
New Mexico	7.1	340	212	-128	-37.6	27.8	23.8	-4.02	-14.5	21	18	-3	-14.3
Wyoming	30.1	466	298	-168	-36.1	10.2	9.8	-0.41	-4.0	3	5	2	66.7
Colorado	15.0	480	311	-170	-35.3	54.6	48.6	-5.99	-11.0	90	75	-15	-16.7
Kansas	8.3	915	657	-259	-28.3	31.8	27.9	-3.99	-12.5	66	50	-16	-24.2
Montana	43.0	544	429	-115	-21.1	12.9	12.1	-0.79	-6.1	14	7	-7	-50.0
Oklahoma	2.9	1142	996	-147	-12.8	44.6	42.0	-2.65	-5.9	52	26	-26	-50.0
Idaho	11.8	595	564	-31	-5.2	18.1	17.4	-0.65	-3.6	25	17	-8	-32.0
Oregon	11.3	763	731	-32	-4.2	35.8	32.3	-3.51	-9.8	55	52	-3	-5.5
Texas	2.9	681	665	-17	-2.5	288.2	260.6	-27.65	-9.6	421	317	-104	-24.7
Washington	11.0	840	1149	309	36.8	62.5	53.7	-8.87	-14.2	76	68	-8	-10.5
Western states total	11.8				-36.9	1100	987.1	-113.3	-10.3	1432	1127	-305	-21.3
US total	8.2	883	769	-114	-12.9	3262	2904	-358.2	-11.0	8877	6294	-2583	-29.1
US cases/100,000 and change										2.37	1.67	-0.70	-29.5

Tm ^o^ C = temperature ^o^ C. The temperature data were simplified to change %.

*A large outbreak of 30 cases occurred in August 2020 in Wisconsin in a factory [49]; these cases were not included in the analyses of this state, subtotal, or eastern total, but included in the US total.

**The data source [44] notes that the vehicle miles in Connecticut in 2020 used preliminary AADT data. The data might overestimate the miles in 2020, leading to decrease of -5.6% from the miles in 2019. In neighboring Massachusetts, the decrease of miles was -16.6% from 2019 to 2020.

***A major outbreak of 136 cases occurred in 2019 in North Carolina due to contaminated hot tubs [4]; these cases were not included in the analyses of this state, subtotal, or eastern total, but included in the US total.

The cases and populations of Alaska and Hawaii were only included in the final US cases/100,000 and change.

New York = New York upstate and New York City.

Calculations of change and %: like Table 6.

Color coding: same as Table 6.

Comparison of incidence drops in the eastern states with precipitation deficit vs surplus: states with deficit, actual = 1881, expected = 5587 x (2164 ÷ 7233) = 1671.5; states with surplus, actual = 283, expected = 1646 x (2164 ÷ 7233) = 492.5, χ ^2^ = 115.3, *P* < 0.001.

In 2021, the recovery of vehicle miles (mean +7.9%) prompted the incidence rises in most states (mean +33.9%) (Table 11). The most incidence rises occurred to 9 eastern states (mean +51.0%) that also showed precipitation surpluses (+3.5 to 29.3%, median +10.0%). These states included 5 states in the New England region. The incidence among the rest 23 eastern states with precipitation deficits (-1.0 to -33.5%, median -6.9%) averaged rises of +31.6%. The differential rises among the eastern states (χ^2^ = 107.6, *P* < 0.001, Table 11 footnotes) again suggested combined or interactive effects of motor traffic and precipitation, as shown in multiple regressions above (Tables 3 and 5). Among the western states with precipitation surpluses vs deficits, the incidence rises were similar (Table 11 footnotes), averaging +22.2%, given the dominance of rising motor traffic (+8.0%). Therefore, in the COVID years, the effects of motor vehicle traffic were dominant and consistent. The interaction with precipitation was also evident. When both motor traffic and precipitation rose or decreased, synergism occurred; when either one rose, the net outcome varied, depending on the dominant effector and/or scale.

**Table 11:** State-wise analysis of the legionellosis cases in 2020 and 2021 with corresponding changes in annual mean temperature, annual precipitation, and vehicle miles traveled in the United States.

US region and State	Tm ^o^ C	Precipitation mm				Vehicle miles in billion				Legionellosis cases			
	+/- %	2020	2021	+/-	%	2020	2021	+/-	%	2020	2021	+/-	%
Eastern states with precipitation deficit													
Virginia	-1.2	1560	1038	-522	-33.5	76.1	80.1	3.99	5.2	205	163	-42	-20.5
North Carolina	-2.1	1690	1209	-481	-28.5	106.3	117.7	11.39	10.7	130	164	34	26.2
Delaware	-2.3	1420	1039	-382	-26.9	8.3	10.2	1.81	21.7	14	24	10	71.4
District of Columbia	-1.6	1457	1120	-337	-23.1	3.0	3.2	0.22	7.2	7	3	-4	-57.1
Maryland	-1.6	1342	1056	-287	-21.3	50.9	56.6	5.72	11.2	182	213	31	17.0
Arkansas	0.7	1621	1276	-345	-21.3	33.9	38.4	4.51	13.3	39	45	6	15.4
West Virginia	-1.8	1366	1082	-284	-20.8	16.1	16.1	0.03	0.2	49	60	11	22.4
South Carolina	-2.8	1521	1206	-315	-20.7	54.0	57.5	3.52	6.5	57	87	30	52.6
Wisconsin*	7.8	868	759	-109	-12.6	57.6	65.0	7.38	12.8	211	225	14	6.6
Tennessee	-1.1	1670	1493	-177	-10.6	76.4	82.6	6.20	8.1	154	163	9	5.8
Kentucky	-0.4	1476	1356	-120	-8.1	46.5	48.1	1.58	3.4	74	116	42	56.8
Michigan	6.2	897	835	-62	-6.9	86.5	96.7	10.20	11.8	378	577	199	52.6
Georgia	-3.0	1536	1451	-84	-5.5	116.0	120.7	4.72	4.1	145	147	2	1.4
Alabama	-2.1	1744	1655	-89	-5.1	67.9	71.9	3.97	5.8	61	91	30	49.2
Missouri	2.1	1177	1118	-59	-5.0	72.8	79.8	6.99	9.6	108	167	59	54.6
Mississippi	-2.4	1749	1664	-85	-4.9	39.7	40.9	1.19	3.0	33	36	3	9.1
Florida	-2.4	1473	1407	-66	-4.5	208.1	217.6	9.49	4.6	428	502	74	17.3
New Jersey	-1.7	1266	1216	-50	-4.0	66.3	73.7	7.33	11.1	241	246	5	2.1
Minnesota	18.3	620	599	-21	-3.4	51.6	57.2	5.55	10.8	94	130	36	38.3
Illinois	2.3	1065	1042	-23	-2.2	94.1	97.5	3.41	3.6	329	523	194	59.0
Maine	6.1	1032	1010	-22	-2.2	13.1	14.6	1.47	11.3	11	39	28	254.5
Ohio	1.0	1075	1059	-16	-1.5	103.1	112.9	9.81	9.5	537	864	327	60.9
Louisiana	-1.9	1701	1684	-17	-1.0	48.4	54.7	6.35	13.1	52	71	19	36.5
Subtotal	-1.6				-6.9	1497	1614	116.8	7.8	3539	4656	1117	31.6
Eastern states with precipitation surplus													
Vermont	1.6	1021	1057	36	3.5	6.0	6.6	0.62	10.3	8	13	5	62.5
Iowa	4.7	744	783	40	5.3	29.8	33.0	3.29	11.1	34	44	10	29.4
Pennsylvania	0.0	1099	1171	72	6.6	88.0	102.7	14.70	16.7	351	454	103	29.3
Indiana	0.9	1077	1154	77	7.2	76.6	78.6	2.03	2.7	155	275	120	77.4
New Hampshire	1.4	1062	1168	106	10.0	12.0	13.1	1.17	9.8	32	58	26	81.3
Rhode Island	-1.4	1131	1266	135	12.0	6.9	7.5	0.66	9.6	36	87	51	141.7
New York	-0.6	998	1175	177	17.7	102.5	106.9	4.39	4.3	693	977	284	41.0
Connecticut**	-2.0	1102	1297	196	17.8	29.8	29.0	-0.86	-2.9	82	118	36	43.9
Massachusetts	-0.5	1077	1392	315	29.3	54.1	59.1	4.99	9.2	139	284	145	104.3
Subtotal	0.0				10.0	405.6	436.6	31.0	7.6	1530	2310	780	51.0
Eastern states total	-0.9				-4.7	1902	2050	147.8	7.8	5069	6966	1897	37.4
Western states with precipitation deficit													
Montana	6.2	429	363	-66	-15.3	12.1	13.5	1.38	11.4	7	15	8	114.3
Oklahoma	0.3	996	854	-142	-14.3	42.0	44.8	2.76	6.6	26	32	6	23.1
Idaho	8.9	564	534	-30	-5.3	17.4	19.3	1.90	10.9	17	20	3	17.6
Washington	1.2	1149	1114	-35	-3.0	53.7	57.8	4.14	7.7	68	85	17	25.0
Oregon	2.4	731	724	-6	-0.9	32.3	36.8	4.54	14.1	52	65	13	25.0
Subtotal	2.4				-5.3	157.5	172.2	14.7	9.3	170	217	47	27.6
Western states with precipitation surplus													
Kansas	2.1	657	692	36	5.4	27.9	31.7	3.84	13.8	50	47	-3	-6.0
Texas	-2.0	665	731	67	10.0	260.6	285.0	24.45	9.4	317	353	36	11.4
South Dakota	8.1	411	461	50	12.2	9.7	10.0	0.25	2.6	10	21	11	110.0
North Dakota	14.6	328	397	69	21.0	8.8	9.3	0.49	5.6	6	5	-1	-16.7
Wyoming	7.4	298	365	67	22.6	9.8	11.1	1.30	13.2	5	7	2	40.0
Nebraska	2.2	463	574	111	23.9	19.4	21.2	1.78	9.1	16	44	28	175.0
Colorado	1.3	311	434	124	39.8	48.6	53.8	5.20	10.7	75	108	33	44.0
New Mexico	-2.5	212	313	101	47.8	23.8	26.8	3.07	12.9	18	22	4	22.2
California	-0.4	307	481	174	56.8	299.8	310.8	11.01	3.7	380	460	80	21.1
Nevada	0.5	149	243	94	63.5	25.2	27.1	1.85	7.3	26	25	-1	-3.8
Utah	1.6	184	344	160	87.2	30.3	33.6	3.39	11.2	32	39	7	21.9
Arizona	-1.6	167	319	153	91.6	65.8	73.8	8.00	12.2	90	112	22	24.4
Subtotal	1.5				31.9	829.6	894.2	64.6	7.8	1025	1243	218	21.3
Western states total	1.6				21.0	987.1	1066	79.3	8.0	1195	1460	265	22.2
US total	0.6	769	774	5	0.7	2904	3132	228.8	7.9	6294	8426	2132	33.9
US cases/100,000 and change										1.67	2.21	0.54	32.6

Tm ^o^ C = temperature ^o^ C. The temperature data were simplified to change %.

*A large outbreak of 30 cases occurred in August 2020 in Wisconsin in a factory [49]; these cases were not included in the analyses of this state, subtotal, or eastern total, but included in the US total.

**The data source [44] notes that the vehicle miles in Connecticut in 2020 used preliminary AADT data. The data might overestimate the miles in 2020, leading to decrease of miles in 2021 by -2.9%, in contrast to the recovery across the US. In other New England states, the miles increased from 2020 to 2021 by 9 to 11%.

The cases and populations of Alaska and Hawaii were only included in the final US cases/100,000 and change.

New York = New York upstate and New York City.

Calculations of change and %: like Table 6.

Color coding: same as Table 6.

Comparison of incidence rises in the eastern states with precipitation deficit vs surplus: states with deficit, actual = 1117, expected = 3539 x (1897 ÷ 5069) = 1324.4, states with surplus, actual = 780, expected = 1530 x (1897 ÷ 5069) = 572.6, χ ^2^ = 107.6, *P* < 0.001.

Comparison of incidence rises in the western states with precipitation deficit vs surplus: states with deficit, actual = 47, expected = 170 x (265 ÷ 1195) = 37.7, states with surplus, actual = 218, expected = 1025 x (265 ÷ 1195) = 227.3, χ ^2^ = 2.68, *P =* 0.10.

In summary of the extensive state-wise analyses above, consistent effects of annual precipitation, temperature, and motor vehicle traffic on the legionellosis incidence were noted across diverse landscapes and climates, particularly among the eastern states. The year-to-year incidence change tended to parallel the rise or fall of precipitation level in the pre-COVID years. In most states, precipitation surplus cooled the annual mean temperature whereas its deficit raised the temperature. Thus, the annual mean temperature infrequently correlated with incidence, which also explained the lack of significant regressions (Table 3).

## Discussion

In this study we have examined the legionellosis incidence rates in the US before and during the COVID-19 pandemic. Before COVID, the age-adjusted legionellosis incidence rates rose exponentially from 1999 to 2019, by an annualized increment of 109.4% or 6.6-fold for the 21 years. In 2020, however, the incidence dropped markedly to 61.7% of the best prediction from the trend, which mirrored the drop of motor vehicle traffic due to COVID containment. The recovery of motor traffic in 2021 also accompanied the return of legionellosis incidence. Robust multiple regressions with the 1999–2021 data not only replicated the strong promotive effect of cumulative temperature anomaly but also showed an enhanced role of road exposure. Analyses of the monthly incidence rates during 1999–2019 before COVID revealed emergence of a seasonal legionellosis incidence plateau in 2018 and 2019. The plateau spanned from June to October in reflection of the combined promotive effects of warmer winters, rising warm days of ≥ 20^o^C, precipitation surplus, and attenuation of solar ultraviolet in the fall. In 2020 and 2021 during COVID, the interactive effects of monthly precipitation and motor vehicle miles were prominent. In particular, the low monthly vehicle miles during COVID in 2020 nearly halved the usual seasonal legionellosis plateau in June-October. These findings thus assigned much of the legionellosis incidence drop in 2020 to less driving or road exposure.

The containment efforts against COVID-19 have also prompted social distancing, restriction on social events from large conventions to house hood parties, and mask wearing. The practice of social distancing should not affect exposure to the soil and aquatic *Legionella* spp., unlike the interpersonal dissemination of COVID. The restriction on social gatherings did reduce outbreaks: the CDC National Outbreak Reporting System (NORS) [51] recorded 41 legionellosis outbreaks with 164 cases (2.6% of all 6310 cases) in 2020, compared to 261 outbreaks with 1398 cases during 2017–2019 (5.3% of 26281) (χ^2^ = 87.8, *P* < 0.001) (S4 Table footnote). Mask wearing was uncommon during driving and inconsistent in the early months during COVID. Thus, these measures had limited effects on the sporadic legionellosis cases in 2020.

Most legionellosis cases manifest pneumonia or Legionnaires’ disease that may lead to hospital visits and/or admission. During COVID, hospitals in the US remained open, even during lockdowns in March and April 2020 before the start of legionellosis seasons. Every patient then required a COVID test to rule out COVID pneumonia. A negative COVID test would prompt other diagnostic tests for diverse etiologic agents to target anti-microbial treatment, as routine medical practice in the US and elsewhere. These considerations thus argue for continued healthcare access and similar diagnosis of legionellosis during COVID.

Therefore, based on the current and recent studies [11,19], the rapidly rising legionellosis incidence in the US can be attributed mostly to climate changes, road exposure, and aging population. The climate changes with 4 decades of warmer temperature and rising precipitation surplus have likely elevated the density of *Legionella* spp. in soil and natural water. The ubiquitous soil bacteria are brought onto paved roads by precipitation and aerosolized by busy motor traffic to expose drivers, passengers, and possibly pedestrians nearby. The massive motor traffic in the US, averaging daily 8–10 billion miles, presents a constant exposure risk. The aging population has also increased the pool of vulnerable hosts for more infections. In 2021, the population of ≥65 years make up 16.8% of all, compared to 12.4% in 2000.

Road exposure is a dynamic and highly variable process involving many interactive events in cascades. Warm temperatures proliferate bacteria in natural water and moist soil. Precipitation raises soil moisture and may deposit soil bacteria onto wet road surfaces. Motor vehicle traffic generates aerosols that may carry soil bacteria to cause exposure. While strong sunlight raises temperature to spur bacterial growth in the soil in long run, it also kills bacteria in the air and dries road surfaces to eliminate aerosols. Conversely, weak sunlight or its absence extends bacterial viability for exposure. The effects of precipitation are also dual: whereas more rainfalls in warm months raise soil moisture to proliferate bacteria and generate road aerosols to aid exposure, the cool temperature after rainfalls dampens bacterial growth, particularly in cold regions. The nature and timing of precipitation, in terms of rainfall or snow, day or night, magnitude, and duration affect road conditions and deposition of bacteria. The traffic speed and density influence aerosolization. Driving with open windows or motorcycles causes expose readily. Incidental events, such as spills of garden sprinklers, roadside constructions, etc., may also deposit bacteria. Overall, road exposure occurs most on wet roads during or after rainfall, in heavy rush hour traffic in urban areas before sunrise or after sunset, in warm months with high bacterial density, particularly in autumn with weakening sunlight, in regions with most conducive soil to bacterial proliferation, and/or with open windows during driving. Finally, an exposed individual may or may not be infected depending on host vulnerability. Therefore, numerous climatic, geographic, and human factors influence the occurrence of legionellosis.

The presence of *Legionella* spp. in natural fresh water requires removal and control of these bacteria in public water systems [53,54]. Faulty water treatment may lead to legionellosis outbreak, such as the ones in Michigan in 2014 and 2015 [55]. However, the infrequency of such outbreaks, as indicated by the CDC NORS database [51], implies general safety of the vast public water systems in the US. During COVID in 2020, more people were restricted to homes, which led to increased use of public water in house hoods. The increase mismatched the drop of legionellosis cases in 2020, which in turn argues for the safety of public water and against a major role of house hood exposure in sporadic cases. During lockdowns, water systems in office buildings, hotels, and resorts were closed or underutilized, causing concerns on the over-growth of *Legionella* spp. in the stagnate water and potential outbreaks. The concern was addressed by the National Lodging Organization who stepped up the monitoring of such water facilities and advocated flushing of outlet faucets as a decontamination measure [56]. It remains to be seen whether this measure also contributed to the reduction of outbreaks in 2020.

The use of public water hardly affects road exposure during or after rainfall or the germicidal activity of solar ultraviolet, shown amply in this study. For example, building plumbing pipes, new or aged, are inside buildings and/or underground without exposure to rainfall or sunlight. The same is true for home shower heads and water tanks, whether it rains or shines matters little to the generation or inhalation of aerosols during shower or bath. Together, it is unlikely that the use of public water contributes much to the overall or sporadic legionellosis cases. By contrast, the soil *Legionella* spp. are free-living (within amoeba host), with density depending on temperature, soil moisture, and other conditions. Aerosols derived from soil or natural water are naturally bioaerosols. Given the rare use or contact of untreated natural water in the US, it is far more common for Americans to expose to bacteria from soil than from natural water bodies.

Soil microbes mainly dwell in rhizosphere in close interactions with plant roots for nutrients and water, ~ 6 inches to 2 feet below surface [57]. The niche protects them from detrimental solar radiation. In the case of ubiquitous soil *Legionella* spp., the depth also limits the spread or contamination of these bacteria unless the soil is disturbed or displaced, such as by excavation, soil work, or rainfall. Excavation has been found to be a risk in sporadic legionellosis [35] as well as the likely cause of outbreaks [7,8]. Construction also digs soil to confer risk to the workers and/or residents nearby [33–35]. Surplus rainfalls may saturate the soil to bring bacterial proliferation to the topsoil, as evidenced by ample diverse *Legionella* spp. in wet soil [21]. From topsoil the bacteria can be readily spread, aerosolized, and/or exposed to sunlight. This reasoning is supported by a recent study with the finding that elevated soil moisture and humidity were linked to increased legionellosis incidence [58].

Travel is a known risk for legionellosis outbreaks as well as sporadic cases [33–37]. Yet, it is infrequent to locate the source of exposure, such as contaminated water at the destination lodging (hotel, resort, etc.) [36]. This fact thus raises the likelihood of road exposure to soil bacteria during transportation, particularly for those who travel only or mainly by driving. Given the incubation period of up to 19 days, road exposure may occur before, during, or after travel. Without travel, daily driving builds mileage quickly to incur the risk. Therefore, road exposure may offer an alternative explanation for travel-associated legionellosis.

Earlier serologic studies around 1980 showed that 10–30% of residents in Michigan and Ohio carried antibodies to *L. pneumophila*, from teenager to senior and more frequently in the summer than in the winter [59–61]. These findings suggest substantial exposure even then to the environmental bacteria. With the US legionellosis incidence being relatively stable in the 1980s-1990s, ~ 1200 annual cases or ~0.50/100,000 [2,20], the ratio of exposure to infection can be estimated on the order of 20,000 to 1. This ratio is far less than those in outbreak settings, such as 10,000 to 23 and 10,000 to 8 in two large outbreaks due to the contamination of indoor whirlpool spas [3,4]. After 4 decades of warming temperature in the US, elevation of the environmental bacteria would lead to more exposure to account for the 6.6-fold rise of the age-adjusted incidence rate. In this regard, a new survey on the prevalence of antibody may be needed.

A recent study from Australia, with a national legionellosis incidence rate of ~2/100,000, showed an antibody prevalence of 32% among blood donors [62,63]. Thus, even considering antibody dynamics and the sensitivity of detection method, exposure to *Legionella* spp. should be far more common than infection, likely around 1000-fold. This ratio speaks for the importance of host vulnerability in developing the infection. It is reasonable to say that >99% of the exposed remain subclinical.

Sporadic cases account for ~95% of all legionellosis cases in the recent two decades [17,51], more than 89% in the 1980s and 1990s [2], which means faster rise of sporadic cases than the rise of outbreak cases. Judged from the robust Log-linear models with predictive accuracy of 85–90% (S5 Table and S8 Table), road exposure can be assumed in most of the sporadic cases (~85%) due to the daily driving of most Americans. This would approximate 8020 of the 9933 cases in 2018 or 5400 of the 6661 cases during the incidence plateau in June-October (S4 Table). With 1408 billion vehicle miles driven in the 5 months, every billion vehicle miles would lead to 4 legionellosis cases in the US. Across the country, Ohio suffers from the highest incidence rate, with 930 cases in 2018 (7.96/100,000) or 682 cases in June-October with corresponding 52.4 billion vehicle miles [11]. Accordingly, every billion vehicle miles would lead to 10–12 cases in the warm months in Ohio.

Worldwide, the legionellosis incidence has also risen drastically in the past two decades, such as in Europe and Japan [64–66], in line with global warming. The isolation of *L. pneumophila* in rainwater on roads in Japan also implies possible road exposure [28]. During COVID in 2020, significant decrease of legionellosis has been seen in Switzerland and Italy [64,65]; whether this was also related to reduced motor traffic may require data and analysis.

In summary, four decades of warming temperature have driven the rapidly rising legionellosis incidence and elevated the density of environmental *Legionella* spp. in the US. The rise is characterized by recent emergence of a seasonal incidence plateau from June to October. Road exposure to soil-derived bacteria likely constitutes the main route in the acquisition of the infection, sporadic cases in particular. The exposure occurs most on wet roads during heavy motor vehicle traffic with attenuated or no sunlight. These findings answer a hanging question on the source of aerosols and exposure in the sporadic infection. This knowledge may be useful in the prevention of this infection, particularly for those vulnerable individuals.

## Supporting information

S1 Table Excel sheets with subtractions of the annual variables from West Virginia (1999–2002) and Oregon (1999–2001) and corrections of the weekly legionellosis cases in some states from 2018 to 2021. (XLSX)

S2 Table Trend of annual mean temperature in representative states in the United States, 1895–2021. (DOCX)

S3 Table Trend of monthly mean temperature (°C) and annual mean ≥ 20°C days in the United States, 1895–2021. (DOCX)

S4 Table Annual legionellosis cases, age-adjusted incidence rates (cases per 100,000 population), mean temperature, precipitation, and vehicle miles driven in the United States, 1999–2021. (DOCX)

S5 Table Age-adjusted annual legionellosis incidence rates and fitting analyses from Log-linear regression model in the United States, 1999–2021. (DOCX)

S6 Table Multiple linear regressions between annual legionellosis incidence rates (cases per 100,000 population, all ages) and concurrent temperature, precipitation, and vehicle miles driven in New Jersey, 1999–2021. (DOCX)

S7 Table Monthly legionellosis cases, incidence rates (cases/100,000), and 14-day ahead-adjusted monthly mean temperature, precipitation, ultraviolet B radiation, and vehicle miles driven in the United States, 2018–2021. (DOCX)

S8 Table Monthly legionellosis cases and fitting analyses from Log-linear regression model and Poisson regression model in the United States, 2018–2021. (DOCX)

S1 Fig Trend of annual mean temperatures (°C, Y axis) in selected US states, 1895–2021 (X axis). A: California (CA) and New Jersey (NJ); B: Ohio (OH), Illinois (IL), and Wisconsin (WI); C: Florida (FL), Massachusetts (MA), and Missouri (MO).
(DOCX)
