# Supplementary material for: Evolution and main causes of the rising legionellosis incidence in the United States
Source: PLOS Glob Public Health. 2026 Sep 9;6(9):e0007077. doi: 10.1371/journal.pgph.0007077 (PMC13557416; doi:10.1371/journal.pgph.0007077)
Supplement: Supplementary file 1 [file pgph.0007077.s002.docx]

S2_Table.docx. Trend of annual mean temperature in representative states in the United States, 1895-2021.

| Location and State | Trend, 1895-2021 | | | Annual mean temperature, °C | | | |
| --- | --- | --- | --- | --- | --- | --- | --- |
|  | Slope | Intercept | R^2^ | 1895-1994 | 1995-2021 | Rise (%) | *P* value |
| Atlantic coast |  |  |  |  |  |  |  |
| Massachusetts, north | 0.0145 | 7.556 | 0.428 | 8.23 | 9.43 | 1.20 (14.6) | 6.6E-14 |
| New Jersey, mid | 0.0166 | 9.955 | 0.4972 | 10.73 | 12.09 | 1.36 (12.7) | 3.2E-16 |
| Florida, south, peninsula | 0.009 | 20.74 | 0.314 | 21.15 | 21.95 | 0.81 (3.8) | 6.6E-12 |
| Central |  | | |  | | |  |
| Wisconsin, north | 0.01 | 5.279 | 0.1535 | 5.71 | 6.70 | 0.99 (17.4) | 3.1E-07 |
| Ohio, mid-east | 0.0074 | 9.906 | 0.127 | 10.21 | 11.00 | 0.79 (7.7) | 7.0E-07 |
| Illinois, mid-west | 0.007 | 10.562 | 0.1034 | 10.87 | 11.53 | 0.66 (6.1) | 9.0E-05 |
| Missouri, central | 0.0043 | 12.311 | 0.045 | 12.48 | 12.98 | 0.51 (4.1) | 0.001 |
| Pacific coast |  | | |  | | |  |
| California | 0.0125 | 13.464 | 0.4774 | 14.04 | 15.08 | 1.04 (7.4) | 5.3E-16 |
| Continental US | 0.0089 | 10.68 | 0.3341 | 11.07 | 11.91 | 0.84 (7.6) | 2.6E-14 |

Interpretation: higher R^2^ value means more consistent correlation or less fluctuation; higher slope means more up trending. For example, the highest R^2^ of 0.4972 and highest slope of 0.0166 were noted in New Jersey. The slope of 0.0166 means a temperature rise of 0.0166^o^C in each successive year, or 1.66^o^C rise in 100 years. By contrast, the slope of 0.0043 in Missouri means a rise of 0.0043^o^C per year or 0.43^o^C per 100 years.
