# Supplementary material for: Evolution and main causes of the rising legionellosis incidence in the United States
Source: PLOS Glob Public Health. 2026 Sep 9;6(9):e0007077. doi: 10.1371/journal.pgph.0007077 (PMC13557416; doi:10.1371/journal.pgph.0007077)
Supplement: Supplementary file 2 [file pgph.0007077.s003.docx]

S3_ Table.docx. Trend of monthly mean temperature (°C) and annual mean ≥ 20°C days in the United States, 1895-2021.

| Month or feature | 1895-2021 | | | 1895-1994 | 1995-2021 | | | Latest 2012-2021 | | |
| --- | --- | --- | --- | --- | --- | --- | --- | --- | --- | --- |
|  | 127 years, trending | | | Century norm | 27 years vs century norm | | | 10 years vs century norm | | |
|  | Slope | Intercept | R^2^ | Mean ± SD | Mean ± SD | Rise (%) | P value | Mean ± SD | Rise (%) | P value |
| January | 0.0115 | – 1.5444 | 0.0614 | - 1.13 ± 1.6879 | 0.37 ± 1.2267 | 1.50 (∞) | 3.3E-05 | 0.70 ± 0.9508 | 1.83 (∞) | 0.001 |
| February | 0.0163 | – 0.0172 | 0.107 | 0.79 ± 1.8378 | 1.92 ± 1.5734 | 1.14 (144) | 0.004 | 1.79 ± 1.9185 | 1.00 (127) | 0.11 |
| March | 0.0146 | 4.5085 | 0.1063 | 5.16 ± 1.5675 | 6.47 ± 1.5489 | 1.30 (25.3) | 1.9E-04 | 6.97 ± 1.8587 | 1.81 (35.0) | 8.9E-04 |
| April | 0.0044 | 8.2515 | 0.0408 | 8.49 ± 0.8038 | 8.82 ± 0.7681 | 0.32 (3.8) | 0.062 | 8.93 ± 0.8056 | 0.43 (5.1) | 0.11 |
| May | 0.0053 | 15.43 | 0.0501 | 15.67 ± 0.8396 | 16.12 ± 0.8856 | 0.45 (2.9) | 0.016 | 16.31 ± 0.9906 | 0.64 (4.1) | 0.026 |
| June | 0.0074 | 19.96 | 0.117 | 20.29 ± 0.7574 | 20.96 ± 0.7175 | 0.68 (3.3) | 5.7E-05 | 21.51 ± 0.6265 | 1.22 (6.0) | 3.0E-06 |
| July | 0.0065 | 22.8 | 0.1262 | 23.08 ± 0.6298 | 23.72 ± 0.6154 | 0.64 (2.8) | 6.5E-06 | 23.90 ± 0.5655 | 0.82 (3.6) | 1.4E-04 |
| August | 0.0065 | 21.98 | 0.1287 | 22.24 ± 0.5923 | 22.95 ± 0.6226 | 0.71 (3.2) | 2.4E-07 | 22.97 ± 0.4519 | 0.73 (3.3) | 2.5E-04 |
| September | 0.0062 | 18.01 | 0.0806 | 18.24 ± 0.7346 | 19.03 ± 0.7788 | 0.80 (4.4) | 2.5E-06 | 19.50 ± 0.5263 | 1.27 (6.9) | 5.9E-07 |
| October | 0.0047 | 12.02 | 0.0317 | 12.24 ± 0.9684 | 12.62 ± 0.9780 | 0.38 (3.1) | 0.076 | 12.88 ± 1.0905 | 0.64 (5.2) | 0.051 |
| November | 0.0096 | 4.9355 | 0.083 | 5.33 ± 1.0546 | 6.36 ± 1.4620 | 1.03 (19.4) | 6.3E-05 | 6.39 ± 1.5903 | 1.06 (19.9) | 0.005 |
| December | 0.0124 | – 0.241 | 0.0962 | 0.33 ± 1.4280 | 1.39 ± 1.3749 | 1.06 (321) | 7.8E-04 | 2.04 ± 1.3725 | 1.71 (517) | 4.6E-04 |
| Annual | 0.0089 | 10.68 | 0.3341 | 11.07 ± 0.4348 | 11.91 ± 0.5050 | 0.84 (7.6) | 2.6E-14 | 12.18 ± 0.5813 | 1.11 (10.0) | 2.7E-11 |
| Days of ≥20°C per year* | Not applicable | | | 80.1 ± 8.23 | 91.1 ± 8.08 | 10.9 (13.6) | 9.8E-09 | 97.6 ± 6.60 | 17.5 (21.8) | 2.7E-09 |
| Start - end dates ≥20°C | Not applicable | | | ≈ Jun. 14 – Sept. 2 | ≈ Jun. 10 – Sept. 9 | | | ≈ Jun. 6 – Sept. 12 | | |

*Estimation by proration from the mean temperatures between May and June and between August and September each year. For example, in 1896, the mean temperatures in May and June were 16.92°C and 21.05°C respectively, making daily temperature increment by 0.1332°C from May to June, and by proration, June 8 as likely start date of 20°C. Similarly, the mean temperatures in August and September 1896 were 22.37°C and 17.19°C respectively, making daily temperature drop by 0.1672°C from August to September, and by proration, August 30 as likely end date of 20°C. From June 8 to August 30, 1896, there were 83 days with temperature ≥20°C.
