# Supplementary material for: Evolution and main causes of the rising legionellosis incidence in the United States
Source: PLOS Glob Public Health. 2026 Sep 9;6(9):e0007077. doi: 10.1371/journal.pgph.0007077 (PMC13557416; doi:10.1371/journal.pgph.0007077)
Supplement: Supplementary file 3 [file pgph.0007077.s004.docx]

S4_Table.docx. Annual legionellosis cases, age-adjusted incidence rates (cases per 100,000 population), mean temperature, precipitation, and vehicle miles driven in the United States, 1999-2021.

| Year | Cases | Age-adjusted cases per  100,000 | Mean temperature, °C | | | Precipitation, mm | | Vehicle miles driven in billions |
| --- | --- | --- | --- | --- | --- | --- | --- | --- |
|  |  |  | **°C** | **+/-** | **Cumulative +/-** | **mm** | **+/-** |  |
| 1895-1994 norm | |  | 11.07 | 0 | 0 | 757.1 | 0 |  |
| 1999 | 1108 | 0.402 | 12.29 | 1.22 | 3.02* | 718.3 | -38.8 | 2636 |
| 2000 | 1127 | 0.407 | 11.93 | 0.86 | 3.89 | 714.1 | -43.0 | 2694 |
| 2001 | 1168 | 0.415 | 12.16 | 1.09 | 4.98 | 736.7 | -20.4 | 2742 |
| 2002 | 1321 | 0.457 | 11.78 | 0.71 | 5.69 | 734.0 | -23.1 | 2836 |
| 2003 | 2232 | 0.754 | 11.81 | 0.74 | 6.43 | 775.0 | 17.9 | 2890 |
| 2004 | 2093 | 0.697 | 11.72 | 0.65 | 7.08 | 844.6 | 87.4 | 2965 |
| 2005 | 2301 | 0.755 | 12.02 | 0.95 | 8.03 | 764.0 | 6.9 | 2989 |
| 2006 | 2834 | 0.917 | 12.36 | 1.29 | 9.33 | 757.4 | 0.3 | 3014 |
| 2007 | 2716 | 0.867 | 12.03 | 0.96 | 10.28 | 741.2 | -15.9 | 3031 |
| 2008 | 3181 | 1.000 | 11.27 | 0.20 | 10.49 | 793.5 | 36.4 | 2977 |
| 2009 | 3522 | 1.091 | 11.33 | 0.26 | 10.74 | 820.4 | 63.3 | 2957 |
| 2010 | 3346 | 1.023 | 11.66 | 0.59 | 11.33 | 796.8 | 39.7 | 2967 |
| 2011 | 4202 | 1.266 | 11.77 | 0.70 | 12.03 | 764.5 | 7.4 | 2946 |
| 2012 | 3688 | 1.086 | 12.93 | 1.86 | 13.89 | 699.3 | -57.8 | 2969 |
| 2013 | 4954 | 1.433 | 11.35 | 0.28 | 14.17 | 788.9 | 31.8 | 2988 |
| 2014 | 5166 | 1.469 | 11.41 | 0.34 | 14.51 | 783.6 | 26.5 | 3026 |
| 2015 | 6079 | 1.705 | 12.44 | 1.37 | 15.88 | 878.6 | 121.5 | 3095 |
| 2016 | 6141 | 1.700 | 12.73 | 1.66 | 17.55 | 798.1 | 41.0 | 3174 |
| 2017 | 7458 | 2.035 | 12.53 | 1.46 | 19.01 | 820.7 | 63.6 | 3212 |
| 2018 | 9933 | 2.691 | 11.96 | 0.89 | 19.89 | 880.1 | 123.0 | 3240 |
| 2019 | 8890 | 2.366 | 11.49 | 0.42 | 20.31 | 883.4 | 126.3 | 3262 |
| 2020 | 6310 | 1.668 | 12.43 | 1.36 | 21.67 | 769.1 | 12.0 | 2904 |
| 2021 | 8442 | 2.212 | 12.51 | 1.43 | 23.11 | 774.2 | 17.1 | 3132 |

*Accumulation of the temperature anomaly started in 1995.

Analysis of legionellosis outbreaks vs all cases from 2017 to 2020: from 2017 to 2019 before COVID, there were 261 outbreaks with 1398 cases, 5.3% of all 26281 cases in these years; in 2020, there were 41 outbreaks with 164 cases, 2.6% of all 6310 cases; the expected outbreak cases in 2020 = 6310 * (1398/26281) = 335.7, χ^2^ = 87.8, *P* < 0.001. The data on outbreaks were from [49].
