# Supplementary material for: Evolution and main causes of the rising legionellosis incidence in the United States
Source: PLOS Glob Public Health. 2026 Sep 9;6(9):e0007077. doi: 10.1371/journal.pgph.0007077 (PMC13557416; doi:10.1371/journal.pgph.0007077)
Supplement: Supplementary file 4 [file pgph.0007077.s005.docx]

S5_Table.docx. Age-adjusted annual legionellosis incidence rates and fitting analyses from Log-linear regression model in the United States, 1999-2021.

|  |  | Log linear model equation Y = 10 ^ (- 1.0442 + 0.0309*temp + 2.9468*v-miles*precip/10,000,000) | | | | | | |
| --- | --- | --- | --- | --- | --- | --- | --- | --- |
|  |  |  |  |  |  |  |  |  |
| Year | **Cases** | **Age-adjusted cases per 100,000** | **Cumulative temp anomaly, °C** | **Precipitation, mm** | **Vehicle miles in billions** | **Predicted incidence rate** | **% of the actual** | **% difference** |
| 1999 | 1108 | 0.402 | 3.02 | 718.3 | 2636 | 0.405 | 100.6 | 0.63 |
| 2000 | 1127 | 0.407 | 3.89 | 714.1 | 2694 | 0.439 | 108.0 | 7.98 |
| 2001 | 1168 | 0.415 | 4.98 | 736.7 | 2742 | 0.507 | 122.1 | 22.10 |
| 2002 | 1321 | 0.457 | 5.69 | 734.0 | 2836 | 0.556 | 121.7 | 21.72 |
| 2003 | 2232 | 0.754 | 6.43 | 775.0 | 2890 | 0.652 | 86.5 | -13.50 |
| 2004 | 2093 | 0.697 | 7.08 | 844.6 | 2965 | 0.818 | 117.2 | 17.24 |
| 2005 | 2301 | 0.755 | 8.03 | 764.0 | 2989 | 0.753 | 99.8 | -0.23 |
| 2006 | 2834 | 0.917 | 9.33 | 757.4 | 3014 | 0.826 | 90.0 | -9.97 |
| 2007 | 2716 | 0.867 | 10.28 | 741.2 | 3031 | 0.862 | 99.4 | -0.59 |
| 2008 | 3181 | 1.000 | 10.49 | 793.5 | 2977 | 0.946 | 94.6 | -5.41 |
| 2009 | 3522 | 1.091 | 10.74 | 820.4 | 2957 | 1.006 | 92.2 | -7.78 |
| 2010 | 3346 | 1.023 | 11.33 | 796.8 | 2967 | 1.006 | 98.4 | -1.61 |
| 2011 | 4202 | 1.266 | 12.03 | 764.5 | 2946 | 0.980 | 77.4 | -22.58 |
| 2012 | 3688 | 1.086 | 13.89 | 699.3 | 2969 | 0.993 | 91.5 | -8.54 |
| 2013 | 4954 | 1.433 | 14.17 | 788.9 | 2988 | 1.226 | 85.5 | -14.45 |
| 2014 | 5166 | 1.469 | 14.51 | 783.6 | 3026 | 1.268 | 86.3 | -13.70 |
| 2015 | 6079 | 1.705 | 15.88 | 878.6 | 3095 | 1.770 | 103.8 | 3.83 |
| 2016 | 6141 | 1.700 | 17.55 | 798.1 | 3174 | 1.756 | 103.3 | 3.31 |
| 2017 | 7458 | 2.035 | 19.01 | 820.7 | 3212 | 2.089 | 102.7 | 2.67 |
| 2018 | 9933 | 2.691 | 19.89 | 880.1 | 3240 | 2.576 | 95.7 | -4.27 |
| 2019 | 8890 | 2.366 | 20.31 | 883.4 | 3262 | 2.709 | 114.5 | 14.50 |
| 2020 | 6310 | 1.668 | 21.67 | 769.1 | 2904 | 1.922 | 115.2 | 15.24 |
| 2021 | 8442 | 2.212 | 23.11 | 774.2 | 3132 | 2.425 | 109.6 | 9.61 |
| \|Mean\| |  |  |  |  |  |  |  | \|9.63\| |

|Mean|= mean value of all |% difference|.
