# Supplementary material for: Evolution and main causes of the rising legionellosis incidence in the United States
Source: PLOS Glob Public Health. 2026 Sep 9;6(9):e0007077. doi: 10.1371/journal.pgph.0007077 (PMC13557416; doi:10.1371/journal.pgph.0007077)
Supplement: Supplementary file 5 [file pgph.0007077.s006.docx]

S6_Table.docx. Multiple linear regressions between annual legionellosis incidence rates (cases per 100,000 population, all ages) and concurrent temperature, precipitation, and vehicle miles driven in New Jersey, 1999-2021.

| Regression and parameters | R^2^ | Coefficient | *P* value | Interpretation |
| --- | --- | --- | --- | --- |
| Incidence rates, 1999-2019, n = 21, before COVID | | | | |
| Adjusted R^2^ | 0.9540 |  | 3.5E-13 | Robust correlation |
| Intercept |  | -1.864 | 2.3E-05 |  |
| Cumulative temperature anomaly, °C |  | 0.099 | 1.6E-11 | Strongly promotive |
| Annual precipitation x vehicle miles |  | 2.61E-05 | 2.8E-06 | Strongly promotive |
|  |  |  |  |  |
| Incidence rates, 1999-2021, n = 23, before and during COVID* | | | | |
| Adjusted R^2^ | 0.9143 |  | 8.2E-12 | Robust correlation |
| Intercept |  | -2.112 | 8.5E-05 |  |
| Cumulative temperature anomaly, °C |  | 0.0824 | 2.9E-10 | Strongly promotive |
| Annual precipitation x vehicle miles |  | 3.07E-05 | 4.6E-06 | Strongly promotive |

For the current analyses, the data were as follows: the 1999-2018 dataset was from ref. 11; the incidence rates were 3.58/100,000 in 2019, 2.60/100,000 in 2020, and 2.65/100,000 in 2021; the annual mean temperatures were 12.39°C in 2019, 13.06°C in 2020, and 12.89°C in 2021, with anomalies of 1.61°C, 2.28°C, and 2.11°C, respectively, making the cumulative temperature anomalies 27.95°C by 2019, 30.24°C by 2020, and 32.35°C by 2021; the annual precipitations were 1319.0 mm in 2019, 1265.7 mm in 2020, and 1215.9 mm in 2021; the vehicle miles driven were 78.2 billion miles in 2019, 66.3 billion miles in 2020, and 73.7 billion miles in 2021.
