# Supplementary material for: Evolution and main causes of the rising legionellosis incidence in the United States
Source: PLOS Glob Public Health. 2026 Sep 9;6(9):e0007077. doi: 10.1371/journal.pgph.0007077 (PMC13557416; doi:10.1371/journal.pgph.0007077)
Supplement: Supplementary file 6 [file pgph.0007077.s007.docx]

S7_Table.docx. Monthly legionellosis cases, incidence rates (cases/100,000), and 14-day ahead-adjusted monthly mean temperature, precipitation, ultraviolet B radiation, and vehicle miles driven in the United States, 2018-2021.

| Year | Month | Cases | /100,000 | Temperature, °C | Precipitation, mm | UVB, kJ/m^2^day/hr. | Vehicle miles, billions |
| --- | --- | --- | --- | --- | --- | --- | --- |
| 2018 | Jan | 350 | 0.0949 | -0.18 | 44.0 | 5271.8 | 255.2 |
|  | Feb | 278 | 0.0753 | 1.94 | 57.9 | 7071.9 | 224.4 |
|  | Mar | 359 | 0.0972 | 4.33 | 71.2 | 12336.5 | 262.3 |
|  | Apr | 303 | 0.0822 | 7.11 | 61.8 | 18817.4 | 269.0 |
|  | May | 469 | 0.1270 | 14.08 | 71.2 | 25945.5 | 284.0 |
|  | Jun | 1125 | 0.3047 | 20.83 | 77.3 | 31447.7 | 278.9 |
|  | Jul | 1184 | 0.3207 | 23.67 | 77.2 | 33731.4 | 291.5 |
|  | Aug | 1490 | 0.4036 | 24.06 | 74.3 | 30148.6 | 287.7 |
|  | Sep | 1429 | 0.3870 | 21.73 | 82.5 | 24404.6 | 271.3 |
|  | Oct | 1433 | 0.3883 | 15.71 | 90.1 | 17260.3 | 279.1 |
|  | Nov | 802 | 0.2173 | 8.10 | 76.1 | 9889.7 | 266.0 |
|  | Dec | 712 | 0.1928 | 2.93 | 77.4 | 5585.3 | 269.8 |
| 2019 | Jan | 551 | 0.1468 | 2.28 | 73.0 | 5233.4 | 257.3 |
|  | Feb | 406 | 0.1082 | -0.01 | 71.0 | 7875.3 | 226.0 |
|  | Mar | 341 | 0.0908 | 1.35 | 72.9 | 13359.2 | 264.1 |
|  | Apr | 350 | 0.0932 | 9.32 | 69.2 | 19256.5 | 270.8 |
|  | May | 582 | 0.1550 | 13.94 | 100.3 | 24277.0 | 285.8 |
|  | Jun | 1057 | 0.2814 | 18.42 | 96.9 | 28863.0 | 280.7 |
|  | Jul | 1221 | 0.3250 | 22.75 | 78.4 | 33083.0 | 293.4 |
|  | Aug | 1053 | 0.2801 | 23.83 | 70.4 | 32125.5 | 289.5 |
|  | Sep | 1187 | 0.3159 | 22.08 | 65.1 | 26263.1 | 273.1 |
|  | Oct | 961 | 0.2558 | 16.10 | 73.5 | 18016.9 | 280.9 |
|  | Nov | 653 | 0.1737 | 7.14 | 62.9 | 10432.0 | 267.6 |
|  | Dec | 526 | 0.1401 | 3.97 | 58.8 | 5777.3 | 271.5 |
| 2020 | Jan | 515 | 0.1361 | 2.77 | 67.8 | 4865.1 | 265.9 |
|  | Feb | 392 | 0.1036 | 2.01 | 65.9 | 7182.3 | 243.3 |
|  | Mar | 301 | 0.0796 | 5.27 | 67.9 | 12334.1 | 241.4 |
|  | Apr | 241 | 0.0637 | 8.70 | 67.4 | 18996.8 | 191.7 |
|  | May | 311 | 0.0822 | 13.45 | 70.7 | 26596.9 | 199.4 |
|  | Jun | 550 | 0.1454 | 19.64 | 72.9 | 33171.4 | 233.3 |
|  | Jul | 733 | 0.1938 | 23.19 | 72.0 | 36657.6 | 262.5 |
|  | Aug | 827 | 0.2186 | 24.06 | 66.3 | 33724.9 | 265.3 |
|  | Sep | 898 | 0.2374 | 21.54 | 60.1 | 25427.1 | 257.1 |
|  | Oct | 624 | 0.1649 | 16.42 | 57.4 | 16949.2 | 266.4 |
|  | Nov | 518 | 0.1369 | 9.41 | 51.9 | 10823.6 | 247.5 |
|  | Dec | 400 | 0.1057 | 4.30 | 50.5 | 6539.8 | 243.6 |
| 2021 | Jan | 273 | 0.0715 | 1.95 | 52.4 | 5276.8 | 232.4 |
|  | Feb | 278 | 0.0728 | -0.15 | 52.3 | 7842.7 | 216.1 |
|  | Mar | 329 | 0.0862 | 3.86 | 58.5 | 13759.5 | 237.3 |
|  | Apr | 371 | 0.0972 | 9.73 | 58.0 | 19963.1 | 256.8 |
|  | May | 552 | 0.1446 | 13.09 | 65.4 | 26210.5 | 265.6 |
|  | Jun | 958 | 0.2510 | 19.88 | 75.8 | 32371.3 | 278.0 |
|  | Jul | 1460 | 0.3826 | 23.47 | 81.7 | 33108.4 | 284.3 |
|  | Aug | 1061 | 0.2780 | 23.98 | 83.0 | 29558.9 | 283.6 |
|  | Sep | 1020 | 0.2673 | 21.99 | 69.6 | 24812.2 | 274.7 |
|  | Oct | 984 | 0.2578 | 16.93 | 71.3 | 17406.4 | 274.5 |
|  | Nov | 631 | 0.1653 | 10.03 | 55.2 | 10283.9 | 268.6 |
|  | Dec | 525 | 0.1376 | 5.70 | 48.2 | 5962.6 | 260.8 |

The monthly case numbers differed slightly from the CDC listed numbers due to minor corrections.

Additional regression models with the 48 monthly data:

Multiple linear regressions with Log-transformed monthly cases by using Excel and SPSS.

| Parameter | R^2^ | Coefficient | *P* value | Interpretation |
| --- | --- | --- | --- | --- |
| Adjusted R^2^ value | 0.8733 |  | 2.2E-20 | Robust correlation |
| Intercept |  | 2.289 | 2.5E-36 |  |
| Monthly mean temperature, °C |  | 0.040 | 3.7E-14 | Strongly promotive |
| Monthly precipitation x vehicle miles |  | 2.288E-05 | 1.3E-07 | Strongly promotive |
| Monthly UVB radiation, KJ/m^2^ day/hr. |  | -2.154E-05 | 3.6E-09 | Strongly inhibitive |

Poisson regressions by using SPSS (with use of natural Log).

| Parameter | B (Coefficient) | Std. Error | 95% Wald Confidence Interval | | Hypothesis Test | | | Exp(B) | 95% Wald CI for Exp(B) | |
| --- | --- | --- | --- | --- | --- | --- | --- | --- | --- | --- |
|  |  |  | Lower | Upper | Wald χ^2^ | df | Sig. |  | Lower | Upper |
| (Intercept) | 5.269 | .0276 | 5.215 | 5.323 | 36467.0 | 1 | <.001 | 194.2 | 184.0 | 205.0 |
| Temperature | .089 | .0017 | .086 | .092 | 2807.6 | 1 | <.001 | 1.093 | 1.089 | 1.097 |
| UVB | -4.506E-5 | 1.349E-6 | -4.77E-5 | -4.24E-5 | 1115.8 | 1 | <.001 | 1.000 | 1.000 | 1.000 |
| Vehicle miles x precipitation | 5.092E-5 | 1.596E-6 | 4.78E-5 | 5.40E-5 | 1018.0 | 1 | <.001 | 1.000 | 1.000 | 1.000 |
| (Scale) | 1^a^ |  |  |  |  |  |  |  |  |  |
| Dependent Variable: Cases  Model: (Intercept), Temperature, UVB, Vehicle miles x precipitation | | | | | | | | | | |
| a. Fixed at the displayed value. | | | | | | | | | | |

Negative binomial regressions by using SPSS (with use of natural Log).

| Parameter | B (Coefficient) | Std. Error | 95% Wald Confidence Interval | | Hypothesis Test | | | Exp(B) | 95% Wald CI for Exp(B) | |
| --- | --- | --- | --- | --- | --- | --- | --- | --- | --- | --- |
|  |  |  | Lower | Upper | Wald χ^2^ | df | Sig. |  | Lower | Upper |
| (Intercept) | 5.256 | .1308 | 4.999 | 5.512 | 1614.0 | 1 | <.001 | 191.7 | 148.3 | 247.7 |
| Temperature | .090 | .0080 | .074 | .106 | 126.1 | 1 | <.001 | 1.094 | 1.077 | 1.112 |
| UVB | -4.832E-5 | 6.387E-6 | -6.08E-5 | -3.58E-5 | 57.2 | 1 | <.001 | 1.000 | 1.000 | 1.000 |
| Vehicle miles x precipitation | 5.423E-5 | 8.163E-6 | 3.82E-5 | 7.02E-5 | 44.1 | 1 | <.001 | 1.000 | 1.000 | 1.000 |
| (Scale) | 1^a^ |  |  |  |  |  |  |  |  |  |
| (Negative binomial) | .031 | .0066 | .020 | .047 |  |  |  |  |  |  |
| Dependent Variable: Cases  Model: (Intercept), Temperature, UVB, Vehicle miles x precipitation | | | | | | | | | | |
| a. Fixed at the displayed value. | | | | | | | | | | |

Kolmogorov-Smirnov normality test by using SPSS.

|  | | | Temp | Precipitation | UVB | Vehicle miles x precipitation | Log incidence rate | Log cases |
| --- | --- | --- | --- | --- | --- | --- | --- | --- |
| N | | | 48 | 48 | 48 | 48 | 48 | 48 |
| Normal Parameters^a,b^ | Mean | | 12.22 | 68.66 | 18881.2 | 18074.1 | -.79144 | 2.78 |
|  | Std. Deviation | | 8.431 | 11.76 | 10273.4 | 4107.1 | .235556 | .235 |
| Most Extreme Differences | Absolute | | .123 | .083 | .117 | .058 | .104 | .112 |
|  | Positive | | .117 | .083 | .117 | .058 | .104 | .105 |
|  | Negative | | -.123 | -.068 | -.117 | -.048 | -.104 | -.112 |
| Test Statistic | | | .123 | .083 | .117 | .058 | .104 | .112 |
| Asymp. Sig. (2-tailed)^c^ | | | .066 | .200^e^ | .098 | .200^e^ | .200^e^ | .170 |
| Monte Carlo Sig. (2-tailed)^d^ | Sig. | | .066 | .548 | .099 | .954 | .214 | .132 |
|  | 99% CI | Lower | .060 | .535 | .092 | .949 | .203 | .123 |
|  |  | Upper | .073 | .561 | .107 | .959 | .224 | .141 |
