# Supplementary material for: Evolution and main causes of the rising legionellosis incidence in the United States
Source: PLOS Glob Public Health. 2026 Sep 9;6(9):e0007077. doi: 10.1371/journal.pgph.0007077 (PMC13557416; doi:10.1371/journal.pgph.0007077)
Supplement: Supplementary file 7 [file pgph.0007077.s008.docx]

S8_Table.docx. Monthly legionellosis cases and fitting analyses from Log-linear regression model and Poisson regression model in the United States, 2018-2021.

|  | | Equation | | | | | | |
| --- | --- | --- | --- | --- | --- | --- | --- | --- |
| Log linear | | Y = 10 ^ (2.289 + 0.040*temp + 2.288*v-miles*precip/100000 - 2.154*UVB/100000) | | | | | | |
| Poisson | | Y = Exp (5.269 + 0.089*temp + 5.092*v-miles*precip/100000 - 4.506*UVB/100000) | | | | | | |
|  |  |  |  | |  | |  | |
|  |  |  | **Model predicted cases** | | **% of model/actual** | | **% difference** | |
| Year | **Month** | **Cases** | **Log linear** | **Poisson** | **Log linear** | **Poisson** | **Log linear** | **Poisson** |
| 2018 | Jan | 350 | 266.2 | 267.1 | 76.0 | 76.3 | -24.0 | -23.7 |
|  | Feb | 278 | 324.5 | 325.2 | 116.8 | 117.0 | 16.8 | 17.0 |
|  | Mar | 359 | 420.5 | 424.3 | 117.2 | 118.2 | 17.2 | 18.2 |
|  | Apr | 303 | 352.8 | 364.9 | 116.3 | 120.2 | 16.3 | 20.2 |
|  | May | 469 | 568.9 | 591.5 | 121.4 | 126.2 | 21.4 | 26.2 |
|  | Jun | 1125 | 864.7 | 901.2 | 76.9 | 80.1 | -23.1 | -19.9 |
|  | Jul | 1184 | 1053.6 | 1098.2 | 89.0 | 92.8 | -11.0 | -7.2 |
|  | Aug | 1490 | 1229.1 | 1261.5 | 82.5 | 84.7 | -17.5 | -15.3 |
|  | Sep | 1429 | 1392.3 | 1399.5 | 97.5 | 98.0 | -2.5 | -2.0 |
|  | Oct | 1433 | 1318.6 | 1300.1 | 92.0 | 90.7 | -8.0 | -9.3 |
|  | Nov | 802 | 728.1 | 716.5 | 90.8 | 89.3 | -9.2 | -10.7 |
|  | Dec | 712 | 579.6 | 567.4 | 81.5 | 79.8 | -18.5 | -20.2 |
| 2019 | Jan | 551 | 497.2 | 488.8 | 90.2 | 88.6 | -9.8 | -11.4 |
|  | Feb | 406 | 306.2 | 308.1 | 75.3 | 75.8 | -24.7 | -24.2 |
|  | Mar | 341 | 312.8 | 319.7 | 91.7 | 93.7 | -8.3 | -6.3 |
|  | Apr | 350 | 473.1 | 485.3 | 135.0 | 138.5 | 35.0 | 38.5 |
|  | May | 582 | 952.0 | 968.3 | 163.5 | 166.3 | 63.5 | 66.3 |
|  | Jun | 1057 | 1060.6 | 1089.5 | 100.3 | 103.1 | 0.3 | 3.1 |
|  | Jul | 1221 | 1025.8 | 1068.1 | 84.0 | 87.5 | -16.0 | -12.5 |
|  | Aug | 1053 | 1035.5 | 1075.0 | 98.4 | 102.1 | -1.6 | 2.1 |
|  | Sep | 1187 | 1028.3 | 1049.7 | 86.6 | 88.4 | -13.4 | -11.6 |
|  | Oct | 961 | 1037.8 | 1033.7 | 108.0 | 107.6 | 8.0 | 7.6 |
|  | Nov | 653 | 542.6 | 540.0 | 83.1 | 82.7 | -16.9 | -17.3 |
|  | Dec | 526 | 487.7 | 480.4 | 92.6 | 91.3 | -7.4 | -8.7 |
| 2020 | Jan | 515 | 509.5 | 499.9 | 98.9 | 97.1 | -1.1 | -2.9 |
|  | Feb | 392 | 381.3 | 380.2 | 97.3 | 97.0 | -2.7 | -3.0 |
|  | Mar | 301 | 406.4 | 410.5 | 135.0 | 136.4 | 35.0 | 36.4 |
|  | Apr | 241 | 333.0 | 345.4 | 138.2 | 143.3 | 38.2 | 43.3 |
|  | May | 311 | 376.3 | 397.4 | 121.0 | 127.8 | 21.0 | 27.8 |
|  | Jun | 550 | 559.8 | 594.8 | 101.8 | 108.1 | 1.8 | 8.1 |
|  | Jul | 733 | 721.1 | 767.6 | 98.4 | 104.7 | -1.6 | 4.7 |
|  | Aug | 827 | 843.9 | 886.2 | 102.0 | 107.2 | 2.0 | 7.2 |
|  | Sep | 898 | 902.0 | 922.5 | 100.4 | 102.7 | 0.4 | 2.7 |
|  | Oct | 624 | 850.6 | 850.3 | 136.3 | 136.3 | 36.3 | 36.3 |
|  | Nov | 518 | 531.3 | 529.8 | 102.6 | 102.3 | 2.6 | 2.3 |
|  | Dec | 400 | 399.4 | 397.0 | 99.9 | 99.3 | -0.1 | -0.7 |
| 2021 | Jan | 273 | 340.1 | 338.6 | 124.6 | 124.0 | 24.6 | 24.0 |
|  | Feb | 278 | 235.9 | 239.4 | 84.8 | 86.1 | -15.2 | -13.9 |
|  | Mar | 329 | 291.2 | 298.7 | 88.5 | 90.8 | -11.5 | -9.2 |
|  | Apr | 371 | 387.8 | 401.2 | 104.5 | 108.1 | 4.5 | 8.1 |
|  | May | 552 | 441.2 | 462.9 | 79.9 | 83.9 | -20.1 | -16.1 |
|  | Jun | 958 | 737.9 | 775.1 | 77.0 | 80.9 | -23.0 | -19.1 |
|  | Jul | 1460 | 1108.1 | 1151.0 | 75.9 | 78.8 | -24.1 | -21.2 |
|  | Aug | 1061 | 1408.6 | 1436.7 | 132.8 | 135.4 | 32.8 | 35.4 |
|  | Sep | 1020 | 1175.0 | 1189.2 | 115.2 | 116.6 | 15.2 | 16.6 |
|  | Oct | 984 | 1092.3 | 1084.3 | 111.0 | 110.2 | 11.0 | 10.2 |
|  | Nov | 631 | 641.7 | 634.9 | 101.7 | 100.6 | 1.7 | 0.6 |
|  | Dec | 525 | 473.7 | 467.5 | 90.2 | 89.0 | -9.8 | -11.0 |
| \|Mean\| |  |  |  |  |  |  | \|15.1\| | \|15.8\| |

|Mean|= mean value of all |% difference|.
