# Supplementary material for: Evolution and main causes of the rising legionellosis incidence in the United States
Source: PLOS Glob Public Health. 2026 Sep 9;6(9):e0007077. doi: 10.1371/journal.pgph.0007077 (PMC13557416; doi:10.1371/journal.pgph.0007077)
Supplement: Supplementary file 8 [file pgph.0007077.s009.docx]

S1_Fig.docx. Trend of annual mean temperatures (°C, Y axis) in selected US states, 1895-2021 (X axis). A: California (CA) and New Jersey (NJ); B: Ohio (OH), Illinois (IL), and Wisconsin (WI); C: Florida (FL), Massachusetts (MA), and Missouri (MO).

|  | CA tmp | NJ tmp | CA norm | NJ norm |
| --- | --- | --- | --- | --- |
| 1895 | 13.6111 | 9.88889 | 14.04 | 10.73 |
| 1896 | 14.1667 | 10.2778 | 14.04 | 10.73 |
| 1897 | 13.6111 | 10.2778 | 14.04 | 10.73 |
| 1898 | 13.7222 | 10.8889 | 14.04 | 10.73 |
| 1899 | 13.6667 | 10.1667 | 14.04 | 10.73 |
| 1900 | 14.1667 | 11.0556 | 14.04 | 10.73 |
| 1901 | 14 | 9.94444 | 14.04 | 10.73 |
| 1902 | 13.4444 | 10.3333 | 14.04 | 10.73 |
| 1903 | 13.4444 | 10.1667 | 14.04 | 10.73 |
| 1904 | 14.1111 | 8.77778 | 14.04 | 10.73 |
| 1905 | 13.9444 | 9.88889 | 14.04 | 10.73 |
| 1906 | 14 | 10.8333 | 14.04 | 10.73 |
| 1907 | 13.6111 | 9.33333 | 14.04 | 10.73 |
| 1908 | 13.4444 | 10.7222 | 14.04 | 10.73 |
| 1909 | 13.4444 | 10.3333 | 14.04 | 10.73 |
| 1910 | 14.2778 | 10.2222 | 14.04 | 10.73 |
| 1911 | 13 | 10.7222 | 14.04 | 10.73 |
| 1912 | 13.0556 | 10 | 14.04 | 10.73 |
| 1913 | 13.5556 | 11.5556 | 14.04 | 10.73 |
| 1914 | 14.1111 | 9.88889 | 14.04 | 10.73 |
| 1915 | 13.7222 | 10.5556 | 14.04 | 10.73 |
| 1916 | 13.1111 | 9.88889 | 14.04 | 10.73 |
| 1917 | 13.6667 | 9.05556 | 14.04 | 10.73 |
| 1918 | 13.7778 | 10.2222 | 14.04 | 10.73 |
| 1919 | 13.5 | 10.8889 | 14.04 | 10.73 |
| 1920 | 13.3889 | 10.0556 | 14.04 | 10.73 |
| 1921 | 14.0556 | 11.6667 | 14.04 | 10.73 |
| 1922 | 13.3889 | 10.7778 | 14.04 | 10.73 |
| 1923 | 13.5556 | 10.3333 | 14.04 | 10.73 |
| 1924 | 13.9444 | 9.66667 | 14.04 | 10.73 |
| 1925 | 13.9444 | 10.6111 | 14.04 | 10.73 |
| 1926 | 14.8889 | 9.38889 | 14.04 | 10.73 |
| 1927 | 13.8333 | 10.5 | 14.04 | 10.73 |
| 1928 | 14.2778 | 10.4444 | 14.04 | 10.73 |
| 1929 | 14 | 10.7222 | 14.04 | 10.73 |
| 1930 | 13.8333 | 10.9444 | 14.04 | 10.73 |
| 1931 | 14.6111 | 11.8333 | 14.04 | 10.73 |
| 1932 | 13.8333 | 11.2222 | 14.04 | 10.73 |
| 1933 | 13.7222 | 11.0556 | 14.04 | 10.73 |
| 1934 | 15.3889 | 10.2778 | 14.04 | 10.73 |
| 1935 | 13.7778 | 10.2778 | 14.04 | 10.73 |
| 1936 | 14.7778 | 10.5556 | 14.04 | 10.73 |
| 1937 | 14 | 10.9444 | 14.04 | 10.73 |
| 1938 | 14 | 11.2222 | 14.04 | 10.73 |
| 1939 | 14.6111 | 11 | 14.04 | 10.73 |
| 1940 | 14.8889 | 9.38889 | 14.04 | 10.73 |
| 1941 | 13.9444 | 11.0556 | 14.04 | 10.73 |
| 1942 | 13.8889 | 10.8333 | 14.04 | 10.73 |
| 1943 | 14.3333 | 10.5 | 14.04 | 10.73 |
| 1944 | 13.4444 | 10.8333 | 14.04 | 10.73 |
| 1945 | 13.9444 | 10.8333 | 14.04 | 10.73 |
| 1946 | 13.7222 | 11.4444 | 14.04 | 10.73 |
| 1947 | 14.2222 | 10.6667 | 14.04 | 10.73 |
| 1948 | 13.1111 | 10.7222 | 14.04 | 10.73 |
| 1949 | 13.4444 | 12.1667 | 14.04 | 10.73 |
| 1950 | 14.5 | 10.6111 | 14.04 | 10.73 |
| 1951 | 14 | 11.1667 | 14.04 | 10.73 |
| 1952 | 13.7778 | 11.3889 | 14.04 | 10.73 |
| 1953 | 14 | 11.9444 | 14.04 | 10.73 |
| 1954 | 14.2222 | 11.2222 | 14.04 | 10.73 |
| 1955 | 13.4444 | 11.1667 | 14.04 | 10.73 |
| 1956 | 13.8889 | 10.7222 | 14.04 | 10.73 |
| 1957 | 13.9444 | 11.3889 | 14.04 | 10.73 |
| 1958 | 14.9444 | 10 | 14.04 | 10.73 |
| 1959 | 15 | 11.6111 | 14.04 | 10.73 |
| 1960 | 14.4444 | 10.6111 | 14.04 | 10.73 |
| 1961 | 14.3333 | 10.8333 | 14.04 | 10.73 |
| 1962 | 14.1111 | 10.1111 | 14.04 | 10.73 |
| 1963 | 13.9444 | 10.2778 | 14.04 | 10.73 |
| 1964 | 13.6667 | 10.8889 | 14.04 | 10.73 |
| 1965 | 13.7222 | 10.5556 | 14.04 | 10.73 |
| 1966 | 14.4444 | 10.6111 | 14.04 | 10.73 |
| 1967 | 14.1667 | 10.1667 | 14.04 | 10.73 |
| 1968 | 14.2222 | 10.7222 | 14.04 | 10.73 |
| 1969 | 14.1667 | 10.6667 | 14.04 | 10.73 |
| 1970 | 14.3333 | 10.7778 | 14.04 | 10.73 |
| 1971 | 13.4444 | 11.1111 | 14.04 | 10.73 |
| 1972 | 14.0556 | 10.5556 | 14.04 | 10.73 |
| 1973 | 14.0556 | 11.8889 | 14.04 | 10.73 |
| 1974 | 14.2222 | 11.1667 | 14.04 | 10.73 |
| 1975 | 13.5 | 11.5556 | 14.04 | 10.73 |
| 1976 | 14.1667 | 10.6111 | 14.04 | 10.73 |
| 1977 | 14.4444 | 11 | 14.04 | 10.73 |
| 1978 | 14.2222 | 10.1111 | 14.04 | 10.73 |
| 1979 | 14.2778 | 10.8889 | 14.04 | 10.73 |
| 1980 | 14.5 | 10.8889 | 14.04 | 10.73 |
| 1981 | 15.1667 | 10.8333 | 14.04 | 10.73 |
| 1982 | 13.5 | 10.7778 | 14.04 | 10.73 |
| 1983 | 14.2222 | 11.4444 | 14.04 | 10.73 |
| 1984 | 14.5556 | 11.2222 | 14.04 | 10.73 |
| 1985 | 14.0556 | 11.3889 | 14.04 | 10.73 |
| 1986 | 14.9444 | 11.2778 | 14.04 | 10.73 |
| 1987 | 14.6111 | 11.3333 | 14.04 | 10.73 |
| 1988 | 14.8889 | 10.8889 | 14.04 | 10.73 |
| 1989 | 14.5 | 10.8333 | 14.04 | 10.73 |
| 1990 | 14.4444 | 12.4444 | 14.04 | 10.73 |
| 1991 | 14.5 | 12.4444 | 14.04 | 10.73 |
| 1992 | 15.1667 | 10.6667 | 14.04 | 10.73 |
| 1993 | 14.2778 | 11.2222 | 14.04 | 10.73 |
| 1994 | 14.4444 | 11.2222 | 14.04 | 10.73 |
| 1995 | 14.9444 | 11.3889 | 14.04 | 10.73 |
| 1996 | 15.3333 | 10.8333 | 14.04 | 10.73 |
| 1997 | 15.0556 | 11.1111 | 14.04 | 10.73 |
| 1998 | 13.7222 | 12.8889 | 14.04 | 10.73 |
| 1999 | 14.4444 | 12.2778 | 14.04 | 10.73 |
| 2000 | 14.8889 | 11.2222 | 14.04 | 10.73 |
| 2001 | 15.0556 | 12 | 14.04 | 10.73 |
| 2002 | 14.8889 | 12.3889 | 14.04 | 10.73 |
| 2003 | 15.2222 | 11 | 14.04 | 10.73 |
| 2004 | 14.9444 | 11.6667 | 14.04 | 10.73 |
| 2005 | 14.7778 | 11.8889 | 14.04 | 10.73 |
| 2006 | 14.7778 | 12.7778 | 14.04 | 10.73 |
| 2007 | 14.9444 | 12 | 14.04 | 10.73 |
| 2008 | 14.9444 | 12 | 14.04 | 10.73 |
| 2009 | 14.8889 | 11.5 | 14.04 | 10.73 |
| 2010 | 14.2778 | 12.6111 | 14.04 | 10.73 |
| 2011 | 14.1111 | 12.7222 | 14.04 | 10.73 |
| 2012 | 15.2778 | 13.2778 | 14.04 | 10.73 |
| 2013 | 15.1667 | 11.7778 | 14.04 | 10.73 |
| 2014 | 16.3333 | 11.0556 | 14.04 | 10.73 |
| 2015 | 16 | 12.1667 | 14.04 | 10.73 |
| 2016 | 15.6111 | 12.7222 | 14.04 | 10.73 |
| 2017 | 15.7222 | 12.5556 | 14.04 | 10.73 |
| 2018 | 15.6111 | 12.3333 | 14.04 | 10.73 |
| 2019 | 14.6667 | 12.3889 | 14.04 | 10.73 |
| 2020 | 15.8333 | 13.0556 | 14.04 | 10.73 |
| 2021 | 15.7778 | 12.8889 | 14.04 | 10.73 |

|  | OH tmp | IL tmp | WI tmp |
| --- | --- | --- | --- |
| 1895 | 9.44444 | 9.83333 | 5.16667 |
| 1896 | 10.5 | 11.2222 | 5.77778 |
| 1897 | 10.3333 | 10.7778 | 5.5 |
| 1898 | 10.7778 | 10.7222 | 6.05556 |
| 1899 | 10.4444 | 10.4444 | 5.27778 |
| 1900 | 10.7778 | 11.1667 | 6.33333 |
| 1901 | 9.72222 | 10.4444 | 5.77778 |
| 1902 | 10.0556 | 10.3333 | 5.88889 |
| 1903 | 9.88889 | 10.1111 | 5.11111 |
| 1904 | 8.83333 | 9.33333 | 4.22222 |
| 1905 | 9.61111 | 10.1667 | 5.11111 |
| 1906 | 10.5 | 10.7778 | 5.88889 |
| 1907 | 9.38889 | 10.2778 | 4.77778 |
| 1908 | 10.7222 | 11.2778 | 6.44444 |
| 1909 | 10.1111 | 10.5 | 5.44444 |
| 1910 | 9.77778 | 10.4444 | 6.05556 |
| 1911 | 11 | 11.5 | 6.22222 |
| 1912 | 9.33333 | 9.66667 | 4.44444 |
| 1913 | 10.8333 | 11.5556 | 6.05556 |
| 1914 | 9.94444 | 11.1111 | 5.66667 |
| 1915 | 9.88889 | 10.5 | 5.61111 |
| 1916 | 9.94444 | 10.6111 | 4.88889 |
| 1917 | 8.22222 | 9.05556 | 3.33333 |
| 1918 | 10.2222 | 10.7222 | 5.66667 |
| 1919 | 10.7222 | 11.1667 | 5.77778 |
| 1920 | 9.55556 | 10.4444 | 5.38889 |
| 1921 | 11.9444 | 12.7778 | 7.55556 |
| 1922 | 10.8889 | 11.6667 | 6.22222 |
| 1923 | 10.1111 | 10.9444 | 5.72222 |
| 1924 | 8.94444 | 9.55556 | 4.16667 |
| 1925 | 10.0556 | 11 | 5.55556 |
| 1926 | 9.33333 | 10.2222 | 4.55556 |
| 1927 | 10.4444 | 11.1111 | 5.33333 |
| 1928 | 9.94444 | 10.7778 | 5.66667 |
| 1929 | 9.61111 | 9.94444 | 4.38889 |
| 1930 | 10.6667 | 11.4444 | 6.33333 |
| 1931 | 11.6111 | 12.7222 | 8.33333 |
| 1932 | 10.7222 | 11.1111 | 5.66667 |
| 1933 | 11.1111 | 12 | 6.22222 |
| 1934 | 10.5556 | 11.7778 | 6.33333 |
| 1935 | 10 | 10.7222 | 5.38889 |
| 1936 | 10.1667 | 11.0556 | 5 |
| 1937 | 9.94444 | 10.3333 | 5.22222 |
| 1938 | 11.2778 | 12.2778 | 6.66667 |
| 1939 | 11 | 11.7778 | 6.61111 |
| 1940 | 9.27778 | 10.2778 | 5.22222 |
| 1941 | 10.8333 | 11.8889 | 7 |
| 1942 | 10.3333 | 10.9444 | 6 |
| 1943 | 9.83333 | 10.5556 | 5.11111 |
| 1944 | 10.5556 | 11.2778 | 6.55556 |
| 1945 | 9.94444 | 10.3889 | 5.11111 |
| 1946 | 11.1111 | 12.0556 | 6.55556 |
| 1947 | 9.94444 | 10.7222 | 5.77778 |
| 1948 | 10.3333 | 10.9444 | 5.88889 |
| 1949 | 11.4444 | 11.5556 | 6.44444 |
| 1950 | 9.66667 | 9.88889 | 4.16667 |
| 1951 | 10 | 9.94444 | 4.33333 |
| 1952 | 10.8889 | 11.4444 | 6.16667 |
| 1953 | 11.2778 | 12.0556 | 6.72222 |
| 1954 | 10.8889 | 12.0556 | 6.22222 |
| 1955 | 10.7222 | 11.3333 | 6.05556 |
| 1956 | 10.3333 | 11.5 | 5.61111 |
| 1957 | 10.4444 | 11 | 5.66667 |
| 1958 | 9.11111 | 10 | 5.55556 |
| 1959 | 10.7222 | 11.0556 | 5.5 |
| 1960 | 9.55556 | 10.1667 | 5.55556 |
| 1961 | 10 | 10.6111 | 6.05556 |
| 1962 | 9.66667 | 10.5 | 5.33333 |
| 1963 | 9.16667 | 10.3889 | 5.55556 |
| 1964 | 10.5 | 11.3889 | 6.33333 |
| 1965 | 10.2222 | 11.1111 | 5.11111 |
| 1966 | 9.5 | 10.3333 | 5.27778 |
| 1967 | 9.72222 | 10.3333 | 4.94444 |
| 1968 | 9.83333 | 10.6111 | 6.11111 |
| 1969 | 9.66667 | 10.2222 | 5.38889 |
| 1970 | 10.3333 | 10.6667 | 5.61111 |
| 1971 | 10.3333 | 11.2222 | 5.55556 |
| 1972 | 9.66667 | 10.1667 | 4.44444 |
| 1973 | 11.2222 | 11.6667 | 6.83333 |
| 1974 | 10.2222 | 10.8889 | 5.66667 |
| 1975 | 10.6111 | 11.1111 | 5.88889 |
| 1976 | 9.38889 | 10.2222 | 5.22222 |
| 1977 | 10 | 10.8333 | 6.05556 |
| 1978 | 9.05556 | 9.77778 | 5 |
| 1979 | 9.38889 | 9.66667 | 4.66667 |
| 1980 | 9.61111 | 10.7222 | 5.66667 |
| 1981 | 9.77778 | 11 | 6.38889 |
| 1982 | 10.1667 | 10.3889 | 5.22222 |
| 1983 | 10.5556 | 11.1111 | 6.33333 |
| 1984 | 10.2222 | 11 | 6.22222 |
| 1985 | 10.2222 | 10.3333 | 5.22222 |
| 1986 | 10.8889 | 11.5556 | 6.27778 |
| 1987 | 11.2778 | 12.2778 | 7.94444 |
| 1988 | 10.2222 | 11.1667 | 6.33333 |
| 1989 | 9.66667 | 10.2222 | 5.05556 |
| 1990 | 11.5 | 12 | 6.94444 |
| 1991 | 11.7222 | 12.0556 | 6.5 |
| 1992 | 10.1667 | 11.0556 | 5.94444 |
| 1993 | 10.2222 | 10.5 | 5.38889 |
| 1994 | 10.2778 | 11.1111 | 6.11111 |
| 1995 | 10.1667 | 10.8889 | 6 |
| 1996 | 9.72222 | 10.0556 | 4.72222 |
| 1997 | 9.94444 | 10.5556 | 5.88889 |
| 1998 | 12.2778 | 12.8889 | 8.5 |
| 1999 | 11.2778 | 11.9444 | 7.44444 |
| 2000 | 10.4444 | 11.2222 | 6.61111 |
| 2001 | 11.3333 | 11.8889 | 7.38889 |
| 2002 | 11.3889 | 11.7778 | 7 |
| 2003 | 10.1111 | 10.9444 | 6.22222 |
| 2004 | 10.7222 | 11.4444 | 6.38889 |
| 2005 | 11 | 12.0556 | 7.27778 |
| 2006 | 11.5 | 12.2222 | 7.77778 |
| 2007 | 11.2778 | 12.0556 | 7.05556 |
| 2008 | 10.3333 | 10.5 | 5.44444 |
| 2009 | 10.2778 | 10.7778 | 5.77778 |
| 2010 | 10.9444 | 11.6111 | 7.5 |
| 2011 | 11.2778 | 11.7222 | 6.72222 |
| 2012 | 12.2222 | 13.2222 | 8.55556 |
| 2013 | 10.5 | 10.6111 | 5.44444 |
| 2014 | 9.55556 | 9.72222 | 4.55556 |
| 2015 | 10.8889 | 11.6111 | 7.11111 |
| 2016 | 12 | 12.6111 | 7.88889 |
| 2017 | 11.7778 | 12.4444 | 7.05556 |
| 2018 | 11.2222 | 11.3889 | 6.22222 |
| 2019 | 11.3333 | 11.1667 | 5.61111 |
| 2020 | 11.6667 | 11.8333 | 7.16667 |
| 2021 | 11.7778 | 12.0556 | 7.61111 |

|  | FL tmp | MA tmp | MO tmp |
| --- | --- | --- | --- |
| 1895 | 20.3889 | 7.72222 | 11.6111 |
| 1896 | 21.0556 | 7.77778 | 13 |
| 1897 | 21.3333 | 8 | 12.6111 |
| 1898 | 21.1667 | 8.44444 | 12.2778 |
| 1899 | 21.3333 | 8 | 12 |
| 1900 | 21.0556 | 8.33333 | 12.8889 |
| 1901 | 20 | 7.55556 | 12.7778 |
| 1902 | 21.0556 | 7.77778 | 12 |
| 1903 | 20.5556 | 7.72222 | 11.6667 |
| 1904 | 20.7222 | 6.27778 | 11.5 |
| 1905 | 21.1111 | 7.27778 | 11.5556 |
| 1906 | 20.8889 | 8.05556 | 12.0556 |
| 1907 | 21.3889 | 7 | 12.3333 |
| 1908 | 21.2778 | 8.27778 | 12.9444 |
| 1909 | 21.2222 | 7.88889 | 12.5556 |
| 1910 | 20.1111 | 8.05556 | 12.2778 |
| 1911 | 21.8333 | 8.11111 | 13.3889 |
| 1912 | 21.1111 | 7.66667 | 11.3333 |
| 1913 | 21.1111 | 8.88889 | 13.0556 |
| 1914 | 20.6111 | 7.33333 | 12.7778 |
| 1915 | 20.6111 | 8.27778 | 12.1667 |
| 1916 | 20.8889 | 7.38889 | 12.3889 |
| 1917 | 20.4444 | 6.5 | 11 |
| 1918 | 21 | 7.61111 | 12.6111 |
| 1919 | 21.3333 | 8.33333 | 12.6111 |
| 1920 | 20.5 | 7.55556 | 12.0556 |
| 1921 | 21.6667 | 9 | 14.2222 |
| 1922 | 21.6667 | 8.16667 | 13.1667 |
| 1923 | 21.1667 | 7.55556 | 12.6111 |
| 1924 | 20.7778 | 7.38889 | 11.3333 |
| 1925 | 21.3333 | 8.16667 | 12.7778 |
| 1926 | 20.7222 | 6.83333 | 12.1111 |
| 1927 | 21.7222 | 8.44444 | 12.7778 |
| 1928 | 20.6667 | 8.22222 | 12.4444 |
| 1929 | 21.5 | 8.22222 | 11.5556 |
| 1930 | 20.6667 | 8.77778 | 12.8889 |
| 1931 | 21.0556 | 9.27778 | 14 |
| 1932 | 21.6667 | 8.66667 | 12.7222 |
| 1933 | 21.6111 | 8.38889 | 13.6111 |
| 1934 | 21 | 7.77778 | 13.6667 |
| 1935 | 21 | 7.77778 | 12.3333 |
| 1936 | 21.1667 | 8.05556 | 13 |
| 1937 | 21 | 8.66667 | 11.8889 |
| 1938 | 21.2222 | 8.77778 | 14.0556 |
| 1939 | 21.4444 | 8.05556 | 13.5556 |
| 1940 | 20.1111 | 7.05556 | 11.6111 |
| 1941 | 21 | 8.55556 | 13.4444 |
| 1942 | 20.7778 | 8.44444 | 12.4444 |
| 1943 | 20.8333 | 7.83333 | 12.2222 |
| 1944 | 21.2222 | 8.38889 | 12.6111 |
| 1945 | 21.5 | 8.44444 | 12 |
| 1946 | 21.7778 | 8.77778 | 13.7222 |
| 1947 | 21.2778 | 8.38889 | 12.4444 |
| 1948 | 22 | 8.16667 | 12.2778 |
| 1949 | 22 | 9.83333 | 12.5556 |
| 1950 | 21.2778 | 8.38889 | 11.6111 |
| 1951 | 21.3333 | 8.88889 | 11.5 |
| 1952 | 21.2778 | 9.11111 | 12.8889 |
| 1953 | 21.5556 | 9.77778 | 13.6667 |
| 1954 | 21.1667 | 8.61111 | 13.8889 |
| 1955 | 21.2222 | 8.61111 | 12.7778 |
| 1956 | 21.0556 | 8 | 13.1667 |
| 1957 | 21.6667 | 9 | 12.5 |
| 1958 | 20.5556 | 7.61111 | 11.6667 |
| 1959 | 21.4444 | 8.83333 | 12.3889 |
| 1960 | 20.7222 | 8.27778 | 11.7222 |
| 1961 | 21.1111 | 8.33333 | 11.8333 |
| 1962 | 21 | 7.55556 | 12.2222 |
| 1963 | 20.6667 | 7.94444 | 12.5 |
| 1964 | 21.1111 | 8.22222 | 12.8333 |
| 1965 | 21.2222 | 7.94444 | 12.7778 |
| 1966 | 20.5556 | 8.27778 | 11.8889 |
| 1967 | 21.2222 | 7.77778 | 12.0556 |
| 1968 | 20.3333 | 8.05556 | 11.8889 |
| 1969 | 20.5556 | 8.38889 | 11.8889 |
| 1970 | 20.7778 | 8.16667 | 12.2778 |
| 1971 | 21.3333 | 8.33333 | 12.6667 |
| 1972 | 21.6111 | 7.72222 | 12.0556 |
| 1973 | 21.2778 | 9.44444 | 12.8889 |
| 1974 | 21.6111 | 8.38889 | 12.4444 |
| 1975 | 21.7778 | 8.88889 | 12.5556 |
| 1976 | 20.5556 | 8.11111 | 12 |
| 1977 | 20.7778 | 8.38889 | 12.6667 |
| 1978 | 20.9444 | 7.61111 | 11.4444 |
| 1979 | 21.0556 | 8.83333 | 11.1111 |
| 1980 | 21.0556 | 8.11111 | 12.7778 |
| 1981 | 20.7222 | 8.44444 | 12.7222 |
| 1982 | 21.9444 | 8.22222 | 11.9444 |
| 1983 | 20.7222 | 9.16667 | 12.2222 |
| 1984 | 21.1111 | 8.88889 | 12.4444 |
| 1985 | 21.5556 | 8.44444 | 11.6667 |
| 1986 | 21.9444 | 8.44444 | 13.2778 |
| 1987 | 21.2222 | 8.55556 | 13.5 |
| 1988 | 20.9444 | 8.44444 | 12.5556 |
| 1989 | 21.6667 | 8.11111 | 11.6667 |
| 1990 | 22.5 | 9.83333 | 13.4444 |
| 1991 | 22.1111 | 9.77778 | 13.3889 |
| 1992 | 21.2778 | 8 | 12.5 |
| 1993 | 21.2778 | 8.5 | 11.6667 |
| 1994 | 22 | 8.72222 | 12.6111 |
| 1995 | 21.5556 | 8.77778 | 12.3333 |
| 1996 | 21.0556 | 8.5 | 11.6111 |
| 1997 | 21.7222 | 8.55556 | 12.0556 |
| 1998 | 22.5 | 10.2222 | 14 |
| 1999 | 21.7778 | 9.88889 | 13.5556 |
| 2000 | 21.4444 | 8.61111 | 12.8333 |
| 2001 | 21.6111 | 9.5 | 13.2222 |
| 2002 | 21.8333 | 9.61111 | 12.9444 |
| 2003 | 21.6111 | 8.38889 | 12.5 |
| 2004 | 21.5556 | 8.66667 | 12.7778 |
| 2005 | 21.5556 | 9.11111 | 13.4444 |
| 2006 | 21.8889 | 10.0556 | 13.7778 |
| 2007 | 22.1111 | 9.05556 | 13.4444 |
| 2008 | 21.5556 | 9.22222 | 11.8889 |
| 2009 | 21.7778 | 8.72222 | 12.2222 |
| 2010 | 20.6667 | 10.2222 | 12.8889 |
| 2011 | 22.0556 | 9.83333 | 13.1111 |
| 2012 | 22.1667 | 10.7222 | 14.7222 |
| 2013 | 22.0556 | 9.33333 | 12 |
| 2014 | 21.4444 | 8.72222 | 11.6111 |
| 2015 | 23 | 9.16667 | 13.2222 |
| 2016 | 22.5 | 10.1667 | 14.1111 |
| 2017 | 22.6667 | 9.77778 | 13.9444 |
| 2018 | 22.3889 | 9.72222 | 12.8889 |
| 2019 | 22.8889 | 9.16667 | 12.7778 |
| 2020 | 22.8889 | 10.4444 | 13.1667 |
| 2021 | 22.3889 | 10.4444 | 13.5 |
