# Supplementary material for: Evolution and main causes of the rising legionellosis incidence in the United States
Source: PLOS Glob Public Health. 2026 Sep 9;6(9):e0007077. doi: 10.1371/journal.pgph.0007077 (PMC13557416; doi:10.1371/journal.pgph.0007077)
Supplement: Supplementary file 9 [file pgph.0007077.s011.docx]

PGPH-D-26-0848 reviewers’ comments and answers

**Reviewer #1:** The manuscript addresses an important and timely public health topic by exploring the relationship between environmental factors and Legionnaires' disease incidence across states. The inclusion of multiple environmental variables and state-level comparisons is a valuable aspect of the study.

Linear regression using monthly mean environmental variables, with model evaluation limited to coefficients, p-values, and R², does not adequately capture the temporal, ecological, and epidemiological structure of Legionella case data. As a result, the present framework seems insufficient to support strong conclusions regarding environmental drivers. The manuscript would be substantially strengthened by a more rigorous modeling strategy, clearer justification of variable selection, more comprehensive diagnostics, and a more cautious interpretation of the findings.

Answer 1.1: This author thanks the reviewer for the assessment and suggestions. Here are the explanations and solutions.

First, the study began with analyses of the annual data with Log-linear regressions. In these analyses, the regressions worked very well, with R square up to 0.96. This modeling approach has been used in our earlier study (ref. 11, PLOS One, 2021, Effects of climate changes and road exposure on the rapidly rising legionellosis incidence rates in the United States). Then an expert biostatistician verified the validity of this approach.

It is natural to continue this modeling strategy with the monthly data to replicate the findings. Indeed, the Log-linear regressions worked reasonably well, with R square up to 0.89, particularly in multiple regressions that improved fittings and showed interactions of the variables, such as the inhibitive effect of UVB. The variations came from the wide range of the monthly variables, such as the freezing temperature in January to 25 C in the summer, as shown in Fig. 6 panels, and from the inter-state variations.

Second, the seasonality of legionellosis from the effects of temperature and precipitation is well known. The solar effects, through UV radiation and sunshine hours, have been examined in detail in another earlier study from us (ref. 19, Appl Env Micro, 2019, Solar and climate effects explain the wide variation in legionellosis incidence rates in the United States). The study showed that the sunlight exerts permissive effect in the winter (as in warm Florida) and fall but suppressive effect in the summer. It was the first systematic and population level study to demonstrate the solar effects on legionellosis incidence across the US. The title of the article used a verb ‘explain’, as suggested by the journal editor when he accepted the article, to illustrate the strong data.

The present study introduced five monthly variables: temperature, precipitation, UVB, vehicle miles, and interactive vehicle miles x precipitation (road exposure). The study replicated the promotive effects of monthly temperature and precipitation, the inhibitive effect of UVB in the summer, and the permissive UVB effect in the fall in the multiple regressions (Table 5) and graphically (Fig. 5A and 6A).

The examination of monthly vehicle miles was the first time in the literature, to this author’s knowledge, following our use of annual vehicle miles in the 2021 PLOS study. The logic and impetus came from the need to demonstrate a specific timing correlation between the drops of monthly legionellosis incidence and vehicle miles in 2020 during COVID. The results were as expected. The pre-COVID regressions were used to set a baseline pattern for every variable in the 24 months of 2018 and 2019. Upon incorporation of the 24 months of 2020 and 2021, the patterns remained. The graphs in Fig. 6 showed the correlation well.

Finally, to explore the suitability of other models, Poisson regressions and negative binomial regressions of the monthly data are performed now by using SPSS. The results are nearly identical to the ones from multiple linear regressions. They are shown in Table S7 footnotes without separate tables. The reasons for the similar results are two-fold: the multiple linear regressions also used Log transformed incidence rates or cases, like the natural log scale in Poisson and negative binomial models, and all variables are in normal or near-normal distribution. For the second reason, Kolmogorov-Smirnov normality tests of the variables are performed and included in the Table S7 footnotes too.

With identical fittings, this author prefers primarily presenting the data from linear regressions to show the fitting R squares and the improvement from simple regressions to multiple regressions. Relevant sentences in M & M and Results are modified accordingly to reflect the addition of Poisson and negative binomial regressions.

Environmental exposures such as precipitation and sunlight are known to influence Legionella transmission with potential delays. However, the current analysis does not clearly describe whether such temporal dynamics were incorporated.
• It would be better to consider lagged exposure variables (e.g., 1–3 week lags).
• If not, please provide justification, as omission of lag effects may lead to biased or attenuated estimates.

Answer 1.2: Yes, the lagging period of 14 days for incubation has been considered in the manuscript, i.e., tracked lines 203-205 in M & M and in Table 5 and supplemental Table S7. For clarity, the adjustment of “14-day ahead” is now used in the text (tracked line 348) and titles of Tables 5 and S7, removing from the table footnotes.

Environmental predictors such as precipitation and sunlight are often correlated.
• Were diagnostics for multicollinearity (e.g., correlation matrices, variance inflation factors) performed?
• If so, please report these results and discuss how collinearity may influence coefficient estimates and interpretation.

Answer 1.3: Yes, Table 5 showed multicollinearity of temperature and UVB when they were analyzed individually with simple linear regression. With multiple linear regressions, their effects were shown better, as explained in the Table 5 footnotes and now also in text for clarity (tracked lines 353-354).

The effects of precipitation and sunlight were opposite, as shown by multiple regressions.

The analysis appears to focus on selected years (e.g., Table 10 & 11) and comparisons relative to annual means. This raises concerns about temporal representativeness and robustness.
• What was the rationale for selecting these specific years?
• Do these years represent extreme events, or were they chosen post hoc?
• Why was the full time series not analyzed systematically?

Answer 1.4: The analyses did begin with systematic assessments of the annual incidence rates and relationships with variables, as detailed in the first several sections, tables, and figures. Monthly data in all 21 years before COVID were also analyzed.

The representative years for state-wise analyses were chosen because of the remarkable changes, such as big rainfall surplus in 2003 as well as the first leap of incidence rate, drought in 2012, incidence peak in 2018, drop of motor vehicle miles due to COVID in 2020, etc. The reasons were given at the beginning of related sections. For example, the analysis of 2002-2003 began with a short paragraph in lines 408-418 for such an explanation.

The 6 tables on state-wise analyses in these years were all large and filled with data, almost dazzling. However, thanks to PLOS, the journal can accommodate them.

No change is made in this regard.

The analysis appears to rely on state-level summaries, which may conceal substantial within-state variation in both exposures and cases.
• How to address the ecological limitations of using aggregated state-level data?
• Could the observed associations be influenced by aggregation bias?
• This limitation better to be discussed more explicitly, particularly if conclusions are framed broadly.

Answer 1.5: This reviewer raises some valid points about geographic variations and their effects on legionellosis. This is exactly why this study analyzed several years of data at state level.

Certainly, within large states, such as Texas and Pennsylvania, there are considerable geographic and/or climate variations at region or county level. It would be ideal to analyze the data at county level for large states.

Our approach to year-to-year comparison for each state should balance a lot of variations within the state. The yearly changes in incidence and climate parameters in a state were also categorized as: significant increase (in green color), minor variation (in black color), and significant decrease (in red color), an attempt to absorb some ‘random’ variations.

Practically speaking, for this study with 50 states and jurisdictions for 23 years, with a population of 300 million and continental US territory of nearly 3 million square miles, it is hardly possible or necessary to obtain county level data for analysis. Even if it is possible, the data quality may be at stake.

Again, the state-wise analyses served two purposes: to see consistency of the effects of precipitation, temperature, and vehicle miles, and to show the variations among the states or multi-state regions.

The 23-year time series was also performed for New Jersey as an example to show the strong interaction between vehicle miles and precipitation (supplemental Table S6). New Jersey is a relatively small state in terms of size, hence less variation within the state. Our previous study (PLOS One 2011, ref. 11) analyzed 5 representative states with the 1999-2018 data for time series, including New Jersey. There is no need to repeat these states in this study.

With these explanations, this author feels that state-level data does not pose serious limitations for the study or warrant discussion. Rather, these analyses are some of the strong points of this study, especially on the consistency and interactions of vehicle miles driven and precipitation in 2020 and 2021 (Tables 10 and 11).

The manuscript would benefit from clarification regarding the handling of missing data. It would be better to indicate whether any missing observations were present in temperature, precipitation, motor vehicle travel, or Legionnaires' disease case data? It would be helpful to describe how these were addressed (e.g., complete case analysis, imputation, or interpolation). Additionally, providing some indication of the sensitivity of the results to the chosen approach for handling missing data would strengthen confidence in the findings.

Answer 1.6: This aspect is now supplemented in new Table S1 to show the corrections and methods for the US weekly cases from 2018 to 2021 and for the lack of data points in population, vehicle miles, precipitation, and temperature in West Virginia (1999-2002) and Oregon (1999-2001).

The correction of weekly US cases improved the weekly and monthly data to some extent but did not affect the annual data.

The corrections of the West Virginia and Oregon data affected each variable by 0-2%. With four years of data, 1999-2002, the corrections improved the regressions slightly more, as shown in Table 3 tracked. For example, in the simple regressions of the 23-year data, the R square for annual precipitation x vehicle miles was improved from 0.6564 to 0.6792 (3.5%).

Given the ecological (state-level) nature of the analysis, there is a risk that the observed association with vehicle travel may be confounded by other factors.
• Were adjustments made for key state-level covariates such as population density, urban infrastructure, or surveillance intensity?

Answer 1.7: This study did not consider population density or urban infrastructure as potential confounders. Again, in the state-wise analyses of 2019-2020 and 2020-2021 cycles during COVID (Tables 10 and 11), the effects of vehicle miles are very consistent, almost all red in 2019-2020 and all green in 2020-2021, with rare exceptions. The size and/or population of a state matter little. Therefore, there should be no major confounding factors to derail the results.

The present study has been based on our previous studies that examined the effects of population and urban driving to some extent. For example, the AEM study in 2019 (ref. 19) considered the population size of New York City in adjusting the temperature for the state of New York. The study also considered the population size and/or location in adjusting sunshine hours in many states. The PLOS One study in 2021 (ref. 11) separated the vehicle miles into urban, rural, and total for analyses. In both papers, these maneuvers did not alter the results much despite being helpful to the fittings.

**Reviewer #2:** The manuscript addresses an important public health question: why legionellosis incidence has increased in the United States and whether climate, precipitation, vehicle miles, UVB, and COVID-era mobility changes explain temporal patterns. The topic is suitable for PLOS Global Public Health, but the present version makes several causal claims that exceed the ecological/time-series evidence presented. PLOS reviewer criteria emphasize technical rigor, sufficient methodological detail, appropriate conclusions supported by data, intelligible reporting, research integrity, and data availability.

Major comments
1. Causal language is too strong for the study design.
The manuscript repeatedly states that warming temperature “has driven” rising legionellosis and that road exposure “likely constitutes the main exposure route” (Abstract lines 36–39; Discussion lines 651–655). These are plausible hypotheses, but the analysis is ecological and correlation-based. The authors should revise to “is associated with,” “is consistent with,” or “supports the hypothesis that.”

Answer 2.1: This point is addressed below for both comments 1 and 2.

2. The exposure pathway is inferred, not directly measured.
The proposed mechanism—soil-derived Legionella deposited on roads by precipitation and aerosolized by traffic—is biologically plausible and discussed extensively (lines 573–590). However, no environmental sampling, road aerosol microbiology, genomic linkage, or individual exposure data are provided. The authors should clearly separate mechanistic speculation from demonstrated epidemiologic associations.

Answers 2.1 and 2: This author thanks this reviewer for the comments on the cause-disease relationship. Yes, a correlation can be casual or causal. In case of a causal relationship, a correlation should exist in reflection of the underlying etiology or biologic process.

The present study demonstrates a strong correlation between the rising legionellosis incidence rates and road exposure (interactive motor vehicle miles x precipitation). This correlation, at the US population level, reflects the underlying microbiologic process. Two new references are cited now in the introduction to show ventilation intake of road aerosols and Legionella by motor vehicles (ref. 29 and 30, tracked lines 91 and 92). The 2024 article (ref. 30) measured aerosols in the truck cabin. The loop of road exposure is now complete: presence of soil bacteria, viable bacteria in rainwater puddles on road surfaces, the physical force of motor vehicle traffic to generate aerosols, and ventilation intake of road aerosols and bacteria by motor vehicles.

The role of warming temperature should also be clear. The bacteria proliferate under warm temperatures. The winters in the US have been warming for over 40 years. There have been more warms days (>20 C) in the past 30 years. The analyses of seasonality and evolution also show this point well.

Together, the underlying microbiology from numerous earlier and recent studies supports these strong statements.

3. Statistical methods need substantial strengthening.
The analyses rely heavily on Excel linear regressions, simple correlations, and multiple regressions (lines 180–190). For time-series infectious disease data, the manuscript should address autocorrelation, seasonality, lag structure, overdispersion, nonlinearity, multicollinearity, and model diagnostics more formally. Excel is not adequate for reproducible epidemiological modeling at this level.

Answer 2.3: The SPSS stats are now used for Poisson and negative binomial regressions for the monthly data. Normality tests for the data are also performed. The results are furnished in Table S7 footnotes. Related sentences are now inserted in M & M and results.

In our previous study (ref. 11, PLOS One, 2021, Effects of climate changes and road exposure on the rapidly rising legionellosis incidence rates in the United States), SPSS was also used to verify the validity of Excel results. It turned out Excel worked well, like this study. Excel is also simple and intuitive. Please also refer to the final point in Answer 1.1 and Answer 1.2.

4. Rationale for not using Poisson or negative binomial models is weak.
The authors state that incidence rates with decimals are unsuitable for Poisson regression and therefore use normal approximation and linear regression (lines 191–197). This is not sufficient. Counts could be modeled with population offsets using Poisson, quasi-Poisson, or negative binomial regression. Monthly and state-level counts are available, so appropriate count models should be considered.

Answer 2.4: Poisson regressions and negative binomial regressions are now performed. Please also refer to the final point in Answer 1.1.

5. Data correction procedures may introduce bias.
The manuscript reports manual corrections for CDC reporting delays and missing data, including “best estimates” for several states and years (lines 148–179). These corrections affect 13.2% of 2020 cases and 5.5% of 2021 cases (lines 172–177). The authors must provide a transparent supplementary file with original values, corrected values, correction rules, and sensitivity analyses using uncorrected data.

Answer 2.5: A new supplemental table is now furnished to show the corrections. Minor additions or modifications to the text are also shown as tracked. Please also refer to Answer 1.6 to the first reviewer on this issue.

6. Data availability statement is insufficient for PLOS.
The statement “All data are included in the manuscript or referenced” is too vague. PLOS requires data necessary to replicate findings to be publicly available without restriction, including individual data points underlying graphs and summary statistics. The authors should deposit cleaned datasets, code, and correction logs in a public repository.

Answer 2.6: Please see above Answer 2.5 and Answer 1.6.

7. Potential confounding is under-addressed.
The manuscript mentions aging population (lines 67–68, 571–572), but does not sufficiently adjust for changes in diagnostic testing, reporting practices, urinary antigen test use, healthcare-seeking behavior, comorbidity prevalence, smoking/COPD prevalence, air-conditioning/building water systems, surveillance intensity, or COVID-related healthcare access.

Answer 2.7: Some of these concerns have been addressed in our PLOS One study. For example, the diagnostic tests for legionellosis, such as media cultivation of bacteria, urinary antigen test, antibody test, and polymerase chain reaction test of the bacterial DNA were all developed in the 1980s and 1990s. As also mentioned in the present study (tracked lines 121-122), the legionellosis incidence has been stable in the 1980s and 1990s, around 1200 cases per year.

The state-wise year-to-year comparisons are also meant to seek consistency across states in view of the variations in population size, climate, and landscape. These analyses should also balance some potential confounders as well.

8. COVID-era interpretation requires more caution.
The reduction in 2020 incidence is attributed mainly to reduced traffic (lines 273–282, 539–554). However, COVID also altered healthcare access, pneumonia testing, hospitalization thresholds, reporting completeness, travel, building occupancy, mask use, and outbreak detection. These competing explanations should be formally discussed and, where possible, analyzed.

Answer 2.8: Three discussion paragraphs have been devoted to some of these aspects, such as social distancing, restriction on large conventions, outbreaks, and travel (tracked lines 573-581, 614-622, and 643-649).

The statement of “The reduction in 2020 incidence is attributed mainly to reduced traffic” is supported by three lines of data: the annual data in 2020 and 2021, along with robust multiple linear correlations, the monthly data in the 48 months of 2018 to 2021 and robust timing correlations during the early months of COVID, and the consistencies in the state-wise comparisons of 2019-2020 (Table 10) and 2020-2021 (Table 11).

Both severe COVID and Legionnaires’ disease present as pneumonia that may lead to hospital visits and/or admission. During COVID, hospitals remained open in the US, even during lockdowns in March and April prior to the legionellosis seasons. Thus, healthcare access was not severely compromised during COVID. Every patient then required COVID test. A diagnosis of pneumonia with a negative COVID test should prompt the search for other etiologies for the sake of effective treatment, a standard practice in the US. These points are now added to the discussion.

9. Global public health relevance needs clearer framing.
The study is entirely U.S.-based. That is acceptable if framed properly, but the manuscript should explicitly state what lessons are transferable to other countries and what is specific to the U.S. road, climate, surveillance, and diagnostic context.

Answer 2.9: Two new paragraphs are added now near the end of discussion, along with 5 new references. They are intended to show the exposure frequency and/or worldwide increase in legionellosis and likely relevance of this study.

10. Reporting guideline compliance is not stated.
This is an observational ecological/time-series study. The authors should include a STROBE checklist or comparable reporting checklist and ensure methods, variables, bias, statistical methods, sensitivity analyses, and limitations are reported transparently. PLOS expects adherence to appropriate reporting guidelines.

Answer 2.10: With the inclusion of a new supplemental table of corrected data, the data accessibility should be better or complete.

Minor comments
• Lines 25–39: The abstract overstates causal conclusions. Add design wording: “ecological analysis of publicly available surveillance and environmental data.”

Answer 2.12: The word ‘ecologic’ is now added in the abstract.

• Lines 83–85: “Dominant and long-sought exposure route” is too strong for the evidence. Revise as a hypothesis.

Answer 2.13: This hypothesis was first raised in our earlier study (ref. 19, Appl Env Micro, 2019, Solar and climate effects explain the wide variation in legionellosis incidence rates in the United States). It was further examined and substantiated in the 2021 PLOS One study. The main points from the two studies have been summarized in the introduction.

With the demonstration of road exposure loop from many earlier and recent studies (see Answer 2.1), this strong state should hold.

Again, road exposure is so common and embedded in our daily lives. Among adults in the US, who does not drive or ride a motor vehicle on a daily or weekly basis? It is almost like asking who does not drink coffee.

In the first case-control study on sporadic Legionnaires’ disease performed in 1977 by CDC, soil-related work, such as construction and excavation were significant risk factors. It is also well known now that travel, professional drivers, and outdoor workers all incur risks. What is the common ground for these risks? It’s soil and the bacteria hidden inside.

This author, a clinical microbiologist and pathologist, has been baffled that, in the 2003 rainfall studies (ref. 12 and 13), those authors did not even discuss soil bacteria. This is like those authors hitting a drum at the edge, not in the center for the best sound. Why does rainfall lead to more cases two weeks later, in just one incubation period? It’s because rainfall causes exposure. Yet, rain drops from the sky should not contain the bacteria, then where do the bacteria come from? Where do rain drops go? Soil. We need some common sense. In 2019, when this author first proposed the significance of road exposure in the US, based on the solar effects, it was not difficult to convince a news reporter. Here is the link: https://www.tmc.edu/news/2019/12/recent-legionnaires-disease-outbreaks-raise-concerns/

• Lines 113–127: Clarify how weekly cases were prorated into months and whether alternative allocation rules changed results.

Answer 2.14: This sentence is now modified into two sentences for clarity as follows.

The weekly cases were also converted to monthly cases according to the calendar. If a week spanned two months, average daily cases of the week were used for calendar integration (tracked lines 116-118).

• Lines 134–144: The UVB derivation needs more detail and validation, especially because 2020 data presentation changed and new 2018–2021 data were used.

Answer 2.15: The annual UVB data for 2020 to 2021 were difficult to obtain from the source (Colorado State Univ.) because they revised the data presentation. For example, the previous format used the unit of KJ/m^2 (1998-2019), but the new format used the unit of KJ/m^2 day/hr. (1998-2021). Why they changed the data listing method was unclear to this author.

Instead of annual data, this author opted to use monthly data from 2018 to 2021 in the new format to demonstrate the UVB effect. To improve understanding, a few sentences in this M & M paragraph are now modified slightly. The gradients of UVB intensity are large for the months, i.e., weak in the winter but strong in the summer, in reflection of the seasonality of sunlight. The results showed a permissive effect in the winter and fall but suppressive effect in the summer, like the previous findings from our 2019 AEM study.

This author has also tried to find other UV data sources, but without much success. For example, the EPA (US Environmental Protection Agency) published monthly UV maps in the US, which were used in the 2019 AEM study, but the maps did not specify a year. The NCI (US National Cancer Institute) has some national UV data, but without multiple months or years.

At any rate, the monthly UVB gradients demonstrate the seasonal effects well.

• Lines 180–190: Provide statistical software, model equations, assumptions, diagnostics, and reproducible code.
• Lines 320–331: The monthly regression interpretation is mechanistically interesting but should include uncertainty intervals and lag sensitivity.

Answer 2.16 and 17: Most of these are now shown in the new supplemental Table S7 footnotes.

• Lines 555–563: The discussion of social distancing and mask wearing is plausible but insufficiently supported analytically. Reframe as a limitation.

Answer 2.18: This discussion seems reasonable. The CDC NORS database is even quoted to show the drop of outbreaks because of the restrictions on social gathering and large conventions. As stated, social distancing breaks interpersonal transmission of the COVID virus, but not the exposure to environmental bacteria. The exposure paths are different.

• Lines 592–610: The argument against household/building water exposure is too categorical. Building water systems remain a recognized legionellosis source; the manuscript should avoid implying minor importance without direct comparative exposure data.

Answer 2.19: Two discussion paragraphs are devoted to the public water systems. The first paragraph began with the importance of removing the bacteria from public water systems, along with three references. The second paragraph discussed the use of public water vs road exposure. The immediate context of the two paragraphs should stay connected.

Yes, the argument against household/building water in the second paragraph is categorical, but also common sense. It is true that building water systems remain a recognized source for legionellosis, and we still have room to improve the systems. Yet, the prominent role of road exposure argues for the general safety of public water systems and “Together, it is unlikely that the use of public water contributes *much* to the overall or sporadic legionellosis cases”.

• Lines 641–650: The estimate that most sporadic cases are due to road exposure is speculative. This section should be clearly labeled as a hypothesis-generating calculation.

Answer 2.20: To support this paragraph, two new supplemental Tables S4 and S8 have now been constructed and included. Among all cases, the annual Log-linear model gives ~90% accuracy and the monthly Log-linear models give ~85% accuracy. It is thus reasonable to assume road exposure in 85% of sporadic cases or 80% of all cases.

**Summary**: This author is indebted to both reviewers for their patience, thorough review, and many constructive comments and suggestions. In this revision, 500 words are now added, 8 new references cited, 3 supplemental tables included, additional SPSS analyses performed, and nearly all comments addressed. The tracking shows them all.

This article is almost 3 times as long as regular articles in terms of text words and illustrations. It is a dissertation. This author intends to use this article to convincingly establish the roles of road exposure and warming temperature in legionellosis.

This year marks the 50 years anniversary of the recognition of legionellosis, unfortunately following a major outbreak. Much has been learned about the etiologic bacteria, from initial isolation and identification of a new genus with a single species to the current 65 species and multiple serogroups, to effective treatment, diagnosis, surveillance and prevention of outbreaks, water management, and so on.

Global warming brings a new dimension to the legionellosis issue, i.e., rapidly rising incidence rates in the past two decades. While outbreaks attract public attention, it is sporadic cases every day that cause most burden to society. The eventual realization of the dominant role of road exposure is also facilitated by an outbreak, the very unfortunate COVID pandemic.

End of comments and answers.

Thank you very much.
