# Supplementary material for: Evolution and main causes of the rising legionellosis incidence in the United States
Source: PLOS Glob Public Health. 2026 Sep 9;6(9):e0007077. doi: 10.1371/journal.pgph.0007077 (PMC13557416; doi:10.1371/journal.pgph.0007077)
Supplement: Peer review history

# Peer review: Decision Letter 0

**DOI:** 10.1371/journal.pgph.0007077.r001

**Authors:** Mohan Amarasiri

5 May 2026

PGPH-D-26-00848

Evolution and Main Causes of the Rising Legionellosis Incidence in the United States

PLOS Global Public Health

Dear Dr. Han,

Thank you for submitting your manuscript to PLOS Global Public Health. After careful consideration, we feel that it has merit but does not fully meet PLOS Global Public Health’s publication criteria as it currently stands. Therefore, we invite you to submit a revised version of the manuscript that addresses the points raised during the review process.

The reviewers have identified the potential of the study. However have raised concerns and provide suggestions including substantial revision of statistical analysis, reframing of conclusions to align with evidence, full transparency of data handling and reproducibility, and expanded discussion of confounding and limitations.

Please submit your revised manuscript by Jul 04 2026 11:59PM. If you will need more time than this to complete your revisions, please reply to this message or contact the journal office at globalpubhealth@plos.org. When you're ready to submit your revision, log on to https://www.editorialmanager.com/pgph/ and select the 'Submissions Needing Revision' folder to locate your manuscript file.

Please include the following items when submitting your revised manuscript:

- A letter that responds to each point raised by the editor and reviewer(s). You should upload this letter as a separate file labeled 'Response to Reviewers'.
- A marked-up copy of your manuscript that highlights changes made to the original version. You should upload this as a separate file labeled 'Revised Manuscript with Track Changes'.
- An unmarked version of your revised paper without tracked changes. You should upload this as a separate file labeled 'Manuscript'.

Guidelines for resubmitting your figure files are available below the reviewer comments at the end of this letter.

As the corresponding author, your ORCID iD is verified in the submission system and will appear in the published article. PLOS supports the use of ORCID, and we encourage all coauthors to register for an ORCID iD and use it as well. Please encourage your coauthors to verify their ORCID iD within the submission system before final acceptance, as unverified ORCID iDs will not appear in the published article. *Only*  the individual author can complete the verification step; PLOS staff *cannot*  verify ORCID iDs on behalf of authors.

We look forward to receiving your revised manuscript.

Kind regards,

Mohan Amarasiri

Academic Editor

PLOS Global Public Health

Journal Requirements:

1. Please ensure that the CRediT author contributions listed for every co-author are completed accurately and in full.

At this stage, the following Authors require contributions: [NAME]. Please ensure that the full contributions of each author are acknowledged in the "Add/Edit/Remove Authors" section of our submission form.

The list of CRediT author contributions may be found here: INSERT LINK

https://journals.plos.org/globalpublichealth/s/authorship#loc-author-contributions

2. Please provide separate figure files in .tif or .eps format.

For more information about figure files please see our guidelines:  LINK

https://journals.plos.org/globalpublichealth/s/figures

https://journals.plos.org/globalpublichealth/s/figures#loc-file-requirements

3. We notice that your supplementary tables are included in the manuscript file. Please remove them and upload them with the file type 'Supporting Information'. Please ensure that each Supporting Information file has a legend listed in the manuscript after the references list.

4. We note that your Data Availability Statement is currently as follows: "All data are included in the manuscript or referenced."

Please confirm at this time whether or not your submission contains all raw data required to replicate the results of your study. Authors must share the “minimal data set” for their submission. PLOS defines the minimal data set to consist of the data required to replicate all study findings reported in the article, as well as related metadata and methods (https://journals.plos.org/plosone/s/data-availability#loc-minimal-data-set-definition).

For example, authors should submit the following data:

- The values behind the means, standard deviations and other measures reported;

- The values used to build graphs;

- The points extracted from images for analysis.

Authors do not need to submit their entire data set if only a portion of the data was used in the reported study.

If your submission does not contain these data, please either upload them as Supporting Information files or deposit them to a stable, public repository and provide us with the relevant URLs, DOIs, or accession numbers. For a list of recommended repositories, please see https://journals.plos.org/plosone/s/recommended-repositories.

If there are ethical or legal restrictions on sharing a de-identified data set, please explain them in detail (e.g., data contain potentially sensitive information, data are owned by a third-party organization, etc.) and who has imposed them (e.g., an ethics committee). Please also provide contact information for a data access committee, ethics committee, or other institutional body to which data requests may be sent. If data are owned by a third party, please indicate how others may request data access.

If the reviewer comments include a recommendation to cite specific previously published works, please review and evaluate these publications to determine whether they are relevant and should be cited. There is no requirement to cite these works unless the editor has indicated otherwise.

Additional Editor Comments (if provided):

[Note: HTML markup is below. Please do not edit.]

Reviewers' comments:

Reviewer's Responses to Questions

**Comments to the Author**

1. Does this manuscript meet PLOS Global Public Health’s publication criteria?

Reviewer #1: Partly

Reviewer #2: Partly

**********

2. Has the statistical analysis been performed appropriately and rigorously?

Reviewer #1: Yes

Reviewer #2: No

**********

3. Have the authors made all data underlying the findings in their manuscript fully available (please refer to the Data Availability Statement at the start of the manuscript PDF file)?

The PLOS Data policy

Reviewer #1: Yes

Reviewer #2: No

**********

4. Is the manuscript presented in an intelligible fashion and written in standard English?

Reviewer #1: Yes

Reviewer #2: No

**********

Reviewer #1: The manuscript addresses an important and timely public health topic by exploring the relationship between environmental factors and Legionnaires' disease incidence across states. The inclusion of multiple environmental variables and state-level comparisons is a valuable aspect of the study.

Linear regression using monthly mean environmental variables, with model evaluation limited to coefficients, p-values, and R², does not adequately capture the temporal, ecological, and epidemiological structure of Legionella case data. As a result, the present framework seems insufficient to support strong conclusions regarding environmental drivers. The manuscript would be substantially strengthened by a more rigorous modeling strategy, clearer justification of variable selection, more comprehensive diagnostics, and a more cautious interpretation of the findings.

Environmental exposures such as precipitation and sunlight are known to influence Legionella transmission with potential delays. However, the current analysis does not clearly describe whether such temporal dynamics were incorporated.

• It would be better to consider lagged exposure variables (e.g., 1–3 week lags).

• If not, please provide justification, as omission of lag effects may lead to biased or attenuated estimates.

Environmental predictors such as precipitation and sunlight are often correlated.

• Were diagnostics for multicollinearity (e.g., correlation matrices, variance inflation factors) performed?

• If so, please report these results and discuss how collinearity may influence coefficient estimates and interpretation

The analysis appears to focus on selected years (e.g., Table 10 & 11) and comparisons relative to annual means. This raises concerns about temporal representativeness and robustness.

• What was the rationale for selecting these specific years?

• Do these years represent extreme events, or were they chosen post hoFF0000?

• Why was the full time series not analyzed systematically?

The analysis appears to rely on state-level summaries, which may conceal substantial within-state variation in both exposures and cases.

• How to address the ecological limitations of using aggregated state-level data?

• Could the observed associations be influenced by aggregation bias?

• This limitation better to be discussed more explicitly, particularly if conclusions are framed broadly.

The manuscript would benefit from clarification regarding the handling of missing data. It would be better to indicate whether any missing observations were present in temperature, precipitation, motor vehicle travel, or Legionnaires' disease case data? It would be helpful to describe how these were addressed (e.g., complete case analysis, imputation, or interpolation). Additionally, providing some indication of the sensitivity of the results to the chosen approach for handling missing data would strengthen confidence in the findings.

Given the ecological (state-level) nature of the analysis, there is a risk that the observed association with vehicle travel may be confounded by other factors.

• Were adjustments made for key state-level covariates such as population density, urban infrastructure, or surveillance intensity?

Reviewer #2: The manuscript addresses an important public health question: why legionellosis incidence has increased in the United States and whether climate, precipitation, vehicle miles, UVB, and COVID-era mobility changes explain temporal patterns. The topic is suitable for PLOS Global Public Health, but the present version makes several causal claims that exceed the ecological/time-series evidence presented. PLOS reviewer criteria emphasize technical rigor, sufficient methodological detail, appropriate conclusions supported by data, intelligible reporting, research integrity, and data availability.

Major comments

1. Causal language is too strong for the study design.

The manuscript repeatedly states that warming temperature “has driven” rising legionellosis and that road exposure “likely constitutes the main exposure route” (Abstract lines 36–39; Discussion lines 651–655). These are plausible hypotheses, but the analysis is ecological and correlation-based. The authors should revise to “is associated with,” “is consistent with,” or “supports the hypothesis that.”

2. The exposure pathway is inferred, not directly measured.

The proposed mechanism—soil-derived Legionella deposited on roads by precipitation and aerosolized by traffic—is biologically plausible and discussed extensively (lines 573–590). However, no environmental sampling, road aerosol microbiology, genomic linkage, or individual exposure data are provided. The authors should clearly separate mechanistic speculation from demonstrated epidemiologic associations.

3. Statistical methods need substantial strengthening.

The analyses rely heavily on Excel linear regressions, simple correlations, and multiple regressions (lines 180–190). For time-series infectious disease data, the manuscript should address autocorrelation, seasonality, lag structure, overdispersion, nonlinearity, multicollinearity, and model diagnostics more formally. Excel is not adequate for reproducible epidemiological modeling at this level.

4. Rationale for not using Poisson or negative binomial models is weak.

The authors state that incidence rates with decimals are unsuitable for Poisson regression and therefore use normal approximation and linear regression (lines 191–197). This is not sufficient. Counts could be modeled with population offsets using Poisson, quasi-Poisson, or negative binomial regression. Monthly and state-level counts are available, so appropriate count models should be considered.

5. Data correction procedures may introduce bias.

The manuscript reports manual corrections for CDC reporting delays and missing data, including “best estimates” for several states and years (lines 148–179). These corrections affect 13.2% of 2020 cases and 5.5% of 2021 cases (lines 172–177). The authors must provide a transparent supplementary file with original values, corrected values, correction rules, and sensitivity analyses using uncorrected data.

6. Data availability statement is insufficient for PLOS.

The statement “All data are included in the manuscript or referenced” is too vague. PLOS requires data necessary to replicate findings to be publicly available without restriction, including individual data points underlying graphs and summary statistics. The authors should deposit cleaned datasets, code, and correction logs in a public repository.

7. Potential confounding is under-addressed.

The manuscript mentions aging population (lines 67–68, 571–572), but does not sufficiently adjust for changes in diagnostic testing, reporting practices, urinary antigen test use, healthcare-seeking behavior, comorbidity prevalence, smoking/COPD prevalence, air-conditioning/building water systems, surveillance intensity, or COVID-related healthcare access.

8. COVID-era interpretation requires more caution.

The reduction in 2020 incidence is attributed mainly to reduced traffic (lines 273–282, 539–554). However, COVID also altered healthcare access, pneumonia testing, hospitalization thresholds, reporting completeness, travel, building occupancy, mask use, and outbreak detection. These competing explanations should be formally discussed and, where possible, analyzed.

9. Global public health relevance needs clearer framing.

The study is entirely U.S.-based. That is acceptable if framed properly, but the manuscript should explicitly state what lessons are transferable to other countries and what is specific to the U.S. road, climate, surveillance, and diagnostic context.

10. Reporting guideline compliance is not stated.

This is an observational ecological/time-series study. The authors should include a STROBE checklist or comparable reporting checklist and ensure methods, variables, bias, statistical methods, sensitivity analyses, and limitations are reported transparently. PLOS expects adherence to appropriate reporting guidelines.

Minor comments

• Lines 25–39: The abstract overstates causal conclusions. Add design wording: “ecological analysis of publicly available surveillance and environmental data.”

• Lines 83–85: “Dominant and long-sought exposure route” is too strong for the evidence. Revise as a hypothesis.

• Lines 113–127: Clarify how weekly cases were prorated into months and whether alternative allocation rules changed results.

• Lines 134–144: The UVB derivation needs more detail and validation, especially because 2020 data presentation changed and new 2018–2021 data were used.

• Lines 180–190: Provide statistical software, model equations, assumptions, diagnostics, and reproducible code.

• Lines 320–331: The monthly regression interpretation is mechanistically interesting but should include uncertainty intervals and lag sensitivity.

• Lines 555–563: The discussion of social distancing and mask wearing is plausible but insufficiently supported analytically. Reframe as a limitation.

• Lines 592–610: The argument against household/building water exposure is too categorical. Building water systems remain a recognized legionellosis source; the manuscript should avoid implying minor importance without direct comparative exposure data.

• Lines 641–650: The estimate that most sporadic cases are due to road exposure is speculative. This section should be clearly labeled as a hypothesis-generating calculation.

**********

what does this mean?). If published, this will include your full peer review and any attached files.

**Do you want your identity to be public for this peer review?**  If you choose “no”, your identity will remain anonymous but your review may still be made public.

For information about this choice, including consent withdrawal, please see our Privacy Policy

Reviewer #1: No

Reviewer #2: No

**********

[NOTE: If reviewer comments were submitted as an attachment file, they will be attached to this email and accessible via the submission site. Please log into your account, locate the manuscript record, and check for the action link "View Attachments". If this link does not appear, there are no attachment files.]

# Author comment: Author response to Decision Letter 1

**DOI:** 10.1371/journal.pgph.0007077.r002

18 Jun 2026

Attachment Submitted filename: PLoS GPH_rev_cmts_rebt.docx

# Peer review: Decision Letter 1

**DOI:** 10.1371/journal.pgph.0007077.r003

**Authors:** Mohan Amarasiri

24 Jul 2026

Evolution and Main Causes of the Rising Legionellosis Incidence in the United States

PGPH-D-26-00848R1

Dear Dr. Han,

We are pleased to inform you that your manuscript 'Evolution and Main Causes of the Rising Legionellosis Incidence in the United States' has been provisionally accepted for publication in PLOS Global Public Health.

Before your manuscript can be formally accepted you will need to complete some formatting changes, which you will receive in a follow up email. A member of our team will be in touch with a set of requests.

Please note that your manuscript will not be scheduled for publication until you have made the required changes, so a swift response is appreciated.

IMPORTANT: The editorial review process is now complete. PLOS will only permit corrections to spelling, formatting or significant scientific errors from this point onwards. Requests for major changes, or any which affect the scientific understanding of your work, will cause delays to the publication date of your manuscript.

If your institution or institutions have a press office, please notify them about your upcoming paper to help maximize its impact. If they'll be preparing press materials, please inform our press team as soon as possible -- no later than 48 hours after receiving the formal acceptance. Your manuscript will remain under strict press embargo until 2 pm Eastern Time on the date of publication. For more information, please contact globalpubhealth@plos.org.

Thank you again for supporting Open Access publishing; we are looking forward to publishing your work in PLOS Global Public Health.

Best regards,

Mohan Amarasiri

Academic Editor

PLOS Global Public Health

***********************************************************

Reviewer Comments (if any, and for reference):

Reviewer's Responses to Questions

**Comments to the Author**

Reviewer #2: All comments have been addressed

**********

publication criteria?

Reviewer #2: Yes

**********

3. Has the statistical analysis been performed appropriately and rigorously?

Reviewer #2: Yes

**********

4. Have the authors made all data underlying the findings in their manuscript fully available (please refer to the Data Availability Statement at the start of the manuscript PDF file)?

The PLOS Data policy

Reviewer #2: Yes

**********

5. Is the manuscript presented in an intelligible fashion and written in standard English?

Reviewer #2: Yes

**********

Reviewer #2: I have no additional comments and wish to convey my congratulations to the authors for their work.

**********

what does this mean?). If published, this will include your full peer review and any attached files.

**Do you want your identity to be public for this peer review?**  If you choose “no”, your identity will remain anonymous but your review may still be made public.

For information about this choice, including consent withdrawal, please see our Privacy Policy

Reviewer #2: **Yes:**  Chandika Damesh Gamage

**********
